# Glycopolymer-Functionalized Gold Nanoparticles for the Detection of Western Diamondback Rattlesnake (Crotalus atrox) Venom

**DOI:** 10.1021/acs.biomac.5c00125

**Published:** 2025-05-20

**Authors:** Mahdi Hezwani, Derecash Anokye, Douglas E. Soutar, Melissa Ligorio, Neil Prabhakar, Jack C. Oram, Alexander J. Cantor, Garrett D. Jackson, Roberto Terracciano, Marc Walker, Alexander N. Baker

**Affiliations:** † Department of Chemistry, University of Warwick, Gibbet Hill Road, CV4 7AL Coventry, U.K.; ‡ Department of Physics, University of Warwick, Gibbet Hill Road, CV4 7AL Coventry, U.K.

## Abstract

Every 5 minutes, 50 people are bitten by a snake worldwide; four will be permanently disabled and one will die. Most approaches to treating and diagnosing snake envenomation, a World Health Organization (WHO)-neglected tropical disease, rely on antibody-based solutions derived from animals or cell culture. Here, we present the first proof of concept for a glycopolymer-based ultraviolet–visible (UV–vis) assay to detect snake venom, specifically Western Diamondback Rattlesnake (Crotalus atrox) venom. This was achieved by synthesizing a library of glycan-terminated poly­(hydroxyethyl acrylamide) functionalized gold nanoparticles. The library was analyzed using UV–vis spectroscopy and biolayer interferometry, with galactose-terminating systems found to demonstrate specificity for C. atrox venom, versus model lectins and Naja naja venom in UV–vis assays. This corroborates glycan array data in the literature and highlights our glycopolymer systems’ potential as a diagnostic tool for snakebite, with the best particle system displaying a limit of detection of ∼20 μg·mL^–1^.

## Graphical abstract

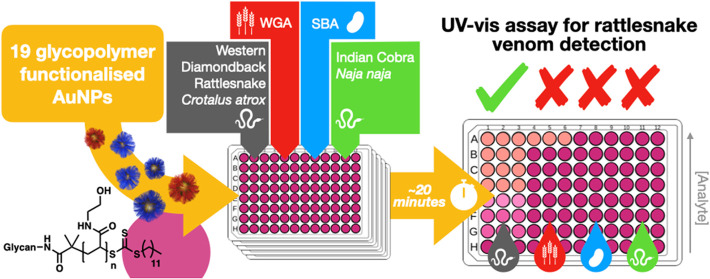

## Introduction

Every 5 minutes, 50 people are bitten by a snake worldwide; four will be permanently disabled and one will die.^1,2^ Snake envenomation is a neglected tropical disease (NTD) that requires urgent attention. The current treatment for snake envenomation utilizes antibody-based antivenoms. Monovalent antivenom (which targets a single venom) treatment is best practice; however, using a monovalent antivenom requires the identification of snake species, as a wrong prescription will be less effective and may have serious consequences.^3,4^ While a polyvalent antivenom can be used, it has adverse patient outcomes and greater risk of side effects.^4^ Identifying envenomation and the snake species responsible for the bite injury is, therefore, vital to improving patient outcomes within the first few hours following envenomation.^5,6^ Current research has focused on antibody-based solutions, in both diagnostics and treatments. However, few robust, low-cost, and commercially available diagnostics are available for snake envenomation.^5^

Observational, qualitative approaches for diagnosis can lead to poor patient outcomes and the injudicious prescribing of inappropriate pharmaceuticals. In snake envenomation, observational approaches rely on bite configuration, knowledge of snake species in the vicinity, talking to witnesses, and clinical manifestations. However, many toxins in snake venoms exhibit similar physiochemical and pharmacological properties and, therefore, similar clinical responses. Furthermore, envenomation from the same snake species but from a different geographical location has been shown to have variations in the venom composition.^7^ Even when the snake is present for identification, there is a chance of misidentification, which can lead to complications in administering antivenoms.^8^ Therefore, rapid, low-cost empirical diagnostics are needed, especially for snakebite victims who are time-critical patients often treated by traditional healers.^9^ There is a pressing need for this technology, with the WHO estimating that snake envenomation (bites) causes ∼100,000 deaths a year and approximately three times as many amputations and permanent disabilities. This is believed to be an underestimate due to a lack of robust reporting.^10–12^

Glycoconjugates (carbohydrates/glycans covalently linked to other chemical species such as proteins, peptides, lipids, etc.) are fundamental to the normal functioning of organisms,^13^ while their inhibition or manipulation can be detrimental,^14,15^ or even fatal.^16–18^ They perform a vast range of roles, including cell signaling,^19^ hormonal action,^20^ cancer progression,^21^ embryonic development,^22^ correct protein folding/structure,^23,24^ and mediating immune responses/infection.^25,26^ Glycans can also be chemically synthesized, so there is no need to raise antibodies, and the sheer range of tools to alter glycan presentation makes them appealing targets.^27–31^ Consequently, there is a significant opportunity to target glycans to differentiate glycoforms, or their lectins (“glycan-readers”), for diagnosis.^32–35^

Lectins are a broad family of glycan-binding proteins that are neither enzymes, transporters, or antibodies. They often have highly conserved carbohydrate-binding domains.^36,37^ Examples of lectins include the Shiga toxin,^38^ cholera toxin,^39^ and ricin.^40^ While also being found in some snake venoms.^41^ Notably, the use of lectins for staining histology samples has been established for decades to identify diseased tissue based on glycosylation.^36^ In a histological study, the binding of glycans to snake venoms was first reported in Bothrops atrox (fer-de-lance, a pit viper) venom.^42^ While other snake venom lectins have been studied, for example, Crotalus atrox (western diamondback rattlesnake) venom (one of the few commercially available venoms with glycan array data),^43^ many Viperidae venoms lack in-depth glycan studies despite constituting up to 10% of venom components in the Viperidae family of over 200 snake species.^44^ The incorporation of glycans into assays for snakebite has not been widely explored.

Plasmonic gold nanoparticles (and other metals)^45^ are important in colorimetric aggregation assays, a valuable diagnostic technique. Mirkin et al. showed that gold nanoparticles functionalized with complementary DNA strands aggregate producing a color shift from red (dispersed) to blue (aggregated).^46^ While Georgiou et al. demonstrated the use of glycan-functionalized nanoparticles for the detection of SARS-COV-2 spike protein, and Richards et al. demonstrated their use for detecting influenza hemagglutinins.^47,48^ This occurs due to the coupling of surface plasmon resonance (SPR) bands, which are measurable by ultraviolet–visible (UV–vis) spectroscopy and often observable by the eye.

## Experimental Section

### Materials

All chemicals were used as supplied, unless otherwise stated. *N-*Hydroxyethyl acrylamide (97%), 4,4′-azobis­(4-cyanovaleric acid) (ACVA, 98%), 4-dimethylaminopyridine (DMAP, >98%), mesitylene (reagent grade), triethylamine (>99%), sodium citrate tribasic dihydrate (>99%), gold­(III) chloride trihydrate (99.9%), ammonia (reagent grade), ammonium carbonate (reagent grade), potassium phosphate tribasic (≥98%, reagent grade), potassium hexafluorophosphate (99.5%), deuterium oxide (D_2_O, 99.9%), deuterated chloroform (CDCl_3_, 99.8%), diethyl ether (≥99.8%, ACS reagent grade), methanol (≥99.8%, ACS reagent grade), toluene (≥99.7%,), HEPES, carbon disulfide (≥99.8%), acetone (≥99%), 1-dodecanethiol (≥98%), lactose (reagent grade), snake venom from C. atrox (Western Diamondback Rattlesnake), potassium trifluoroacetate (98%), super-DHB (≥99%), and pentafluorophenol (≥99%, reagent plus) were purchased from Sigma-Aldrich. Dimethylformamide (DMF) (>99%) and 2-bromo-2-methylpropionic acid (98%) were purchased from Acros Organics. Galactosamine HCl, glucosamine HCl, mannosamine HCl, 1-amino-1-deoxy-*N*-acetyl-galactosamine, and 1-ethyl-3-(3-(dimethylamino)­propyl)­carbodiimide hydrochloride (EDCI, >98%) were purchased from Carbosynth. High-performance liquid chromatography (HPLC)-grade acetonitrile (≥99.8%), glucose (lab-reagent grade), hexane fraction from petrol (lab-reagent grade), DCM (99% lab reagent grade), sodium hydrogen carbonate (≥99%), ethyl acetate (≥99.7%, analytical reagent grade), sodium chloride (≥99.5%), calcium chloride, 40–60 petroleum ether (lab reagent grade), hydrochloric acid (∼37%, analytical grade), glacial acetic acid (analytical grade), and magnesium sulfate (reagent grade) were purchased from Thermo Fisher Scientific. Soybean agglutinin (SBA) and wheat germ agglutinin (WGA) were purchased from Vector Laboratories. Naja naja (Indian cobra) venom was purchased from Latoxan. Trifluoroacetic acid 99.9% was purchased from ABCR. Ultrapure water used for buffers was of Milli-Q grade 18.2 MΩ resistance.

### Methods

#### Representative Synthesis of Poly­(hydroxyethyl acrylamide)

PFP-DMP (367 mg, 0.86 mmol), which was synthesized in an earlier step (see the Supporting Information), was dissolved in a 50:50 mixture of toluene:methanol (22 mL) before adding the monomer 2-hydroxyethyl-acrylamide (2.05 g, 17.8 mmol) and 4,4-azobis (4-cyanovaleric acid) (40 mg, 0.26 mmol) under an atmosphere of nitrogen for 30 min. The mixture was then heated for 2 h at 70 °C before polymerization was terminated by immersing in a bath of liquid nitrogen. The solvent was then removed under reduced pressure. The polymer was then dissolved with minimal methanol before precipitation with diethyl ether and centrifuged at 13,000 rpm (18,900*g*) for 10 min. The supernatant was decanted, and this process was repeated a further two times before drying the product at low pressure for 16 h to afford a light yellow solid.

#### Representative Glycan-Functionalization of pHEA_42_ Using 1-Deoxy-1-amino-galactose

Poly­(*N*-hydroxyethyl acrylamide) (PHEA) (200 mg, 0.039 mmol) was dissolved in DMF (20 mL) along with the glycan (1-amino-1-deoxy-galactose as representative) (0.078 mmol) and triethylamine (TEA) (0.05 M). The reaction was subsequently stirred for 16 h at 50 °C before the solvent was removed under pressure. The crude product was then dissolved in a minimal amount of methanol before precipitation with cold diethyl ether. The mixture was centrifuged for 2 min at 13,000 rpm (18,900*g*), and the supernatant was decanted off. The product was then dissolved again with the minimal amount of methanol before removing the solvent under reduced pressure to give an orange/brown crystalline solid. The loss of the fluorine signal in ^19^F NMR was used to indicate that the reaction had gone to completion.

#### 16 nm Gold Nanoparticle Functionalization

5 mg of each glycopolymer was agitated overnight with 2.5 mL of 16 nm AuNPs at 1Abs at UV_MAX_. The solution was then centrifuged at 12,500 rpm (9800*g*) for 30 min before the pellet was resuspended in 2 mL of water. This washing was repeated a further two times before all of the nanoparticles were concentrated to OD 2 by dilution of the pellet with 50 μL of water at a time and measurement of the UV_MAX_.

#### 30 nm and 40 nm Gold Nanoparticle Functionalization

5 mg of each glycopolymer was agitated overnight with 2.5 mL of the AuNPs at 1Abs at UV_MAX_. The solution was then centrifuged at 8000 rpm (4200*g*) for 30 min before the pellet was resuspended in 2 mL of water. This washing was repeated a further two times before all of the nanoparticles were concentrated to OD 2 by dilution of the pellet with 50 μL of water at a time and measurement of the UV_MAX_.

#### 4 × HEPES Buffer Preparation

0.952 g of HEPES (40 mmol·dm^–3^), 3.51 g of sodium chloride (0.6 mmol·dm^–3^), 0.0044 g of calcium chloride (0.4 mmol·dm^–3^), and 0.53 mg of manganese chloride (0.042 mmol·dm^–3^) were dissolved in 100 mL of distilled water and stirred for 30 min to yield a 4 × concentrated buffer.

#### Aggregation Assay Protocol with Varying Lectin Concentrations and Fixed AuNP OD

An aggregation assay was prepared in a 96-well plate by the addition of 12.5 μL of functionalized gold nanoparticles. Then, a serial dilution of 50 μL of 2 mg·mL^–1^ lectin (SBA, WGA, *C. atrox* venom), diluting with Milli-Q water, to attain 25 μL aliquots of concentrations 8, 6,4, 2, 1, 0.5, 0.25, 0.125, 0.063, and 0.031 mg·mL^–1^ was added. Finally, 12.5 μL of the 4 × HEPES buffer was added to give a total volume in the well of 50 μL. This was then analyzed by UV–vis in a range of 400–700 nm after incubation at RTP (∼20 °C) for 20 min.

## Results and Discussion

Herein, we report the design and synthesis of an initial proof of concept for a glycopolymer-functionalized gold nanoparticle-based UV–vis assay for the detection of C. atrox venom. Our system consists of gold nanoparticles functionalized with glycans on a polymer tether. The polymeric tether, poly­(*N*-hydroxyethyl acrylamide) (pHEA), was synthesized by thermally initiated reversible addition–fragmentation chain transfer (RAFT) polymerization, using 2-(dodecylthiocarbonothioylthio)-2-methylpropionic acid pentafluorophenyl ester (PFP-DMP) as the RAFT agent, following methodologies previously described by Micallef et al.^49^ This produced polymers with predictable chain lengths and low dispersity. The RAFT agent and polymers were characterized by SEC, mass spectrometry, Fourier transform infrared (FTIR) spectroscopy, and ^1^H, ^13^C, and ^19^F NMR analyses (Table 1). A RAFT agent with an activated ester as the R group was chosen to allow for facile installation of an amino-glycan at the α-chain end of polymers. Furthermore, the trithiocarbonate ω-chain end has known affinity for gold (aurophilic), necessary for immobilization of the polymer onto the gold nanoparticle surface (Figure S142).^50^

**Table 1:** Synthesized Polymers

polymer	[CTA]:[M]^a^	conversion, %	Mn SEC (g mol^–1^)^b^	DP SEC^b^	DP NMR^c^	*Đ* ^b^
pHEA_52_	1:50	99	6500	52	48	1.35
pHEA_42_	1:25	98	5400	42	25	1.20
pHEA_25_	1:10	93	3400	25	11	1.11

a Molar ratio of chain transfer agent (CTA) to monomer (M).

b Determined by SEC in DMF with 5 mM NH_4_BF_4_ eluent, calibrated with poly­(methyl methacrylate) standards at 50 °C at flow rate of 1.0 mL·min^–1^.

c Determined from ^1^H NMR end-group analysis. Polymers are referred to by their DP SEC in this paper.

Rattlesnake venom lectin (RSVL) in C. atrox venom has known affinity toward *N*-acetyl-galactosamine, galactose, and lactose (a disaccharide with a terminal galactose) residues.^43^ Soybean agglutinin (SBA) from Glycine max and wheat germ agglutinin (WGA) from Triticum vulgaris were chosen as the control lectins. SBA has known affinity to α/β-*N*-acetyl-galactosamine and, to a lesser extent, galactose residues.^51^ Glucose has known affinity to WGA, whereas mannose has affinity to a range of lectins including the mannose-binding lectin (MBL) and the C-type lectin found in the venom of Oxyuranus scutellatus (Australian coastal taipan).^52–54^

Six aminoglycans were therefore substituted for the activated ester at the α-terminus of the polymer. 2-Deoxy-2-amino-glucose (glucosamine) (Glc-2), 2-deoxy-2-amino-mannose (mannosamine) (Man-2), and 2-deoxy-2-amino-galactose (galactosamine) (Gal-2) were purchased from commercial suppliers, while 1-amino-1-deoxy-lactose (Lac-1), 1-amino-1-deoxy-*N*-acetyl-galactosamine (GalNAc-1), and 1-amino-1-deoxy-galactose (Gal-1) were synthesized (see the Supporting Information). Two synthetic methods were employed to achieve amine functionalization at the anomeric position of lactose, galactose, and *N*-acetyl-galactosamine. The first was a two-step method using 2-azido-1-3-dimethylimidazolinium hexafluorophosphate (ADMP) to substitute the anomeric hydroxyl for an azide before using a Pd/C catalyst to reduce the azide to an amine.^35^ The second utilized ammonium bicarbonate and ammonia to substitute the anomeric hydroxyl directly to an amine on both galactose and lactose.^55,56^ Products from both methods were analyzed by mass spectrometry, NMR, and FTIR to observe the loss of hydroxyl and gain of amine groups. Comparing the two synthetic methods, the literature suggested that using ADMP in the former synthetic method results in a controlled set of amino-glycan anomers.^57^ This is important as lectins found in certain snake venoms, such as Crotalus ruber, displayed β-galactoside anomeric specificity over the α-anomer.^58^ Additionally, a study by Young et al. has shown that C. atrox has displayed specificity toward terminal α-Gal and α-GalNAc; however, there is currently little evidence if this trend is consistent with other glycans.^43^

For the first round of testing, the six aminoglycans were added to pHEA_42_ before immobilization onto gold nanoparticles synthesized by a seeded growth mechanism (16 nm) or purchased (40 nm).^59^ This produced a library of 12 glycosylated nanoparticles, which were characterized by dynamic light scattering (DLS, Figures S6–S45), transmission electron microscopy (TEM, Figure S2), UV–vis spectroscopy (UV–vis, Figures S3–S5), and X-ray photoelectron spectroscopy (XPS, Figures S116–S131, Tables S2 and S3) (Figure 1).

**Figure 1:**
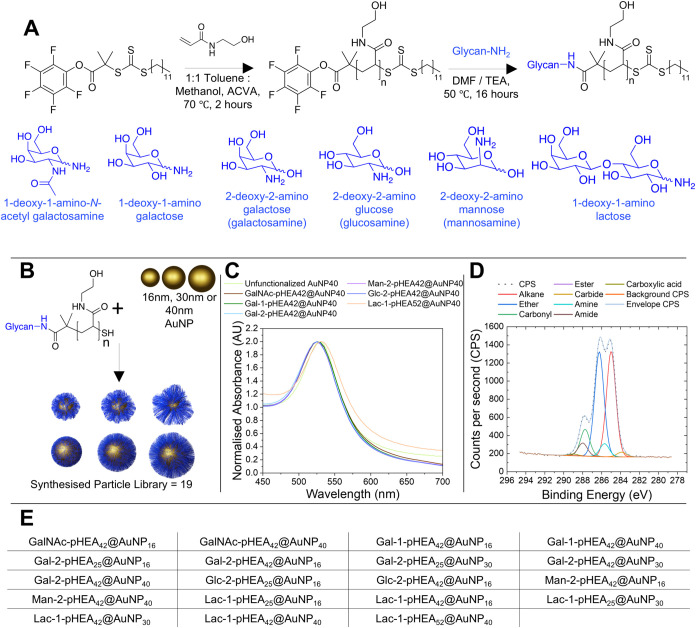
Synthesis of gold nanoparticle library with glycan-terminated polymer tethers and analysis. (A) Polymerization of *N*-hydroxyethyl acrylamide synthesis by RAFT, followed by displacement of the PFP ester with aminoglycans, (B) gold nanoparticle functionalization with glycopolymer, (C) representative UV–vis spectra of (40 nm) nanoparticle systems, (D) C 1*s* X-ray photoelectron spectrum of Gal-2-pHEA_42_@AuNP_40_, and (E) written representation of all synthesized nanoparticles.

Elemental analysis was conducted on dried particles using XPS to determine their surface composition, which confirmed polymer on the gold surface due to the presence of an N 1*s* peak; nitrogen is not present in unfunctionalized (citrate-stabilized) AuNP samples or background. Furthermore, the presence of amide (C­(O)­NC) and amine (C­(O)­N*C*) peaks in the C 1*s* (Figure 1D), and in the N 1*s* scans (amine and amides have similar/overlapping binding energies so were not distinguishable), showed the presence of pHEA. This alongside shift in the UV_MAX_, indicative of changes to the AuNP surface composition, confirmed polymer addition to the gold surface.

In the Lac-1-pHEA_42_@AuNP_40_ system, partial or full aggregation of glycosylated nanoparticles was observed by eye (a noticeable change from deep red to blue/purple or colorless). Aggregation was confirmed by UV–vis and DLS prior to the plasmonic assay testing, indicating a colloidally unstable system, which was hence unsuitable for this study and removed from all further testing. In this case, the polymer chain may not have been able to provide colloidal stability for the larger molecular weight lactose disaccharide and the larger 40 nm particle. The literature has discussed this phenomenon before with similar systems and is further supported as Lac-1-pHEA_42_@AuNP_16_ was stable.^49^

To determine the stability of each permutation of AuNP and pHEA, a UV–vis assay was undertaken in triplicate using a fixed OD_MAX(SPR)_ of 0.5 of each AuNP system and varying concentration of SBA, WGA (1, 0.5, 0.25, 0.125, 0.0625, 0.03125, 0.0156 mg·mL^–1^) and C. atrox venom (8, 6, 4, 2, 1, 0.5, 0.25, 0.125, 0.0625, 0.03125, and 0.0156 mg·mL^–1^) to determine the sensitivity and specificity of each system. Absorbance was measured following incubation at room temperature (∼20 °C) for 20 min (Figures S46–S115). The C. atrox venom, while a real-world target, is known to contain a variety of proteins, including RSVL, a C-type lectin in a relatively low quantity (1–2%), whereas the SBA and WGA are pure lectins.^43,60^ Hence, a higher concentration of the venom was used. All absorbances were normalized at OD_450_ to allow for direct comparison.

After running the plasmonic assays, partial/full nonspecific aggregation was observed for several systems which displayed colloidal instability, including GalNAc-pHEA_42_@AuNP_40_ and Gal-1-pHEA_42_@AuNP_40_, so were excluded from further studies as to not provide false-positives. Strong binding is indicated by variations seen at Abs_700_ and/or SPR bands coupling with a shift/drop at UV_MAX_ in the UV–vis spectra. To quantify this, the change at Abs_700_ versus a 0 mg·mL^–1^ analyte control was determined. This was observed in one or both of the control lectins with various systems; Glc-2-pHEA_42_@AuNP_40_ (Figures 3H and S87–S90) bound to WGA and SBA, and Gal-2-pHEA_42_@AuNP_16_ bound to SBA (Figures 3E and S61–S64), and Man-2-pHEA_42_@AuNP_16_ bound to SBA (Figures 3I and S90–94), with previous literature supporting these results.^52,61–68^ Since these systems showed no binding to the C. atrox venom, these were removed from all future testing. Furthermore, Glc-2-pHEA_42_@AuNP_16_ (Figures 2K–2N, 3G, and S82–S86), Gal-1-pHEA_42_@AuNP_16_ (Figures 3C and S53–56) and GalNAc-1-pHEA_42_@AuNP_16_ (Figures 3A and S46–S49) displayed little to no affinity toward any of the lectins, so were also excluded from future testing, while Man-2-pHEA_42_@AuNP_40_ (Figures 3J and S95–S98) aggregated with C. atrox venom, along with both WGA and SBA, indicating nonspecific binding.

**Figure 2:**
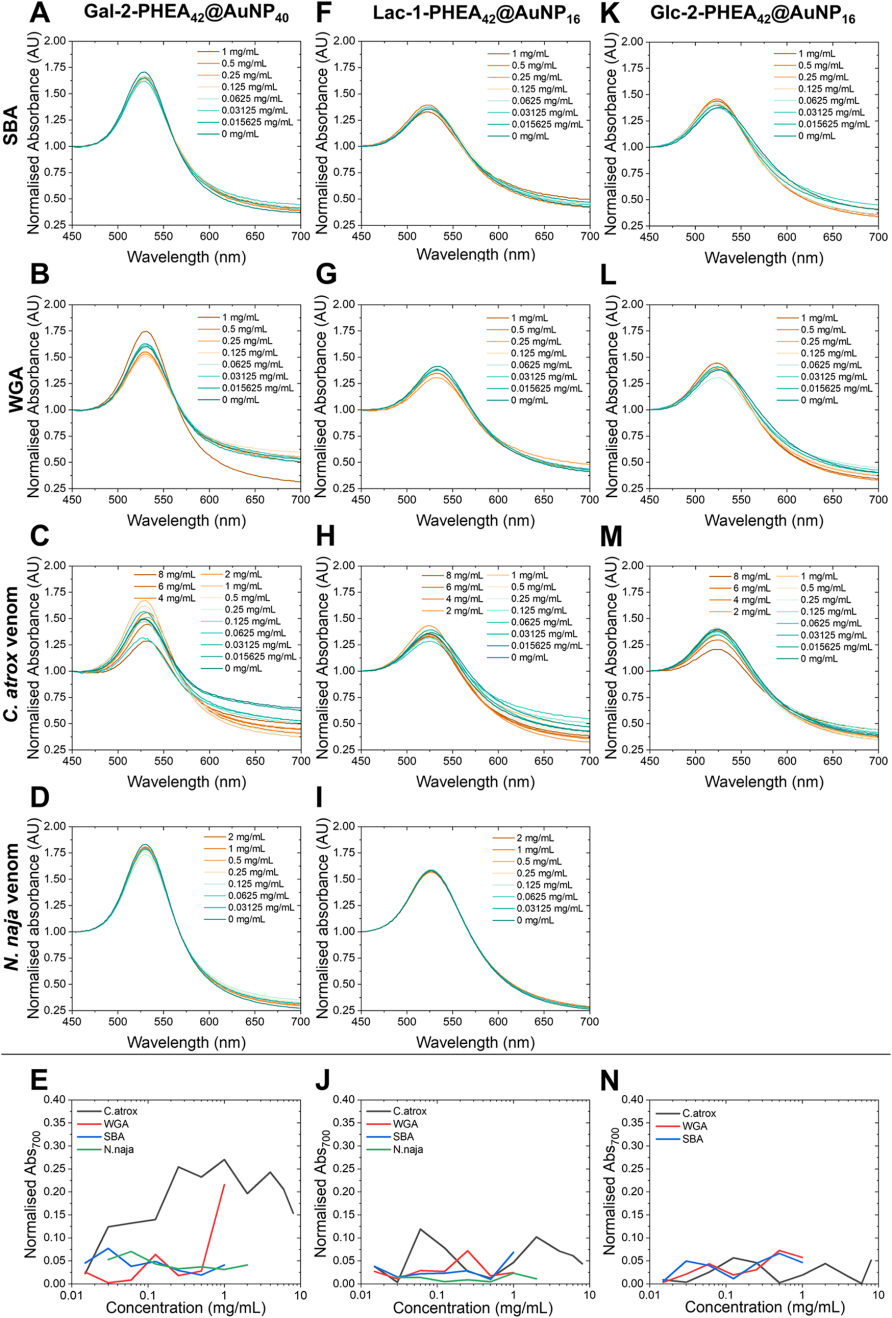
UV–vis absorbance spectra of three particle systems (Gal-2-PHEA_42_@AuNP_40_ versus SBA (A), WGA (B), C. atrox venom (C) and N. naja venom (D); Lac-1-PHEA_42_@AuNP_16_ versus SBA (F), WGA (G), and C. atrox venom (H) and N. naja venom (I); and Glc-2-PHEA_42_@AuNP_16_ versus SBA (K), WGA (L) and C. atrox venom (M)) versus varying concentrations of analyte. Normalized absorbance at 700 nm for the three particle systems (Gal-2-PHEA_42_@AuNP_40_ (E), Lac-1-PHEA_42_@AuNP_16_ (J), and Glc-2-PHEA_42_@AuNP_16_ (N)) versus SBA, WGA, C. atrox venom, and N. naja venom at varying concentrations.

**Figure 3:**
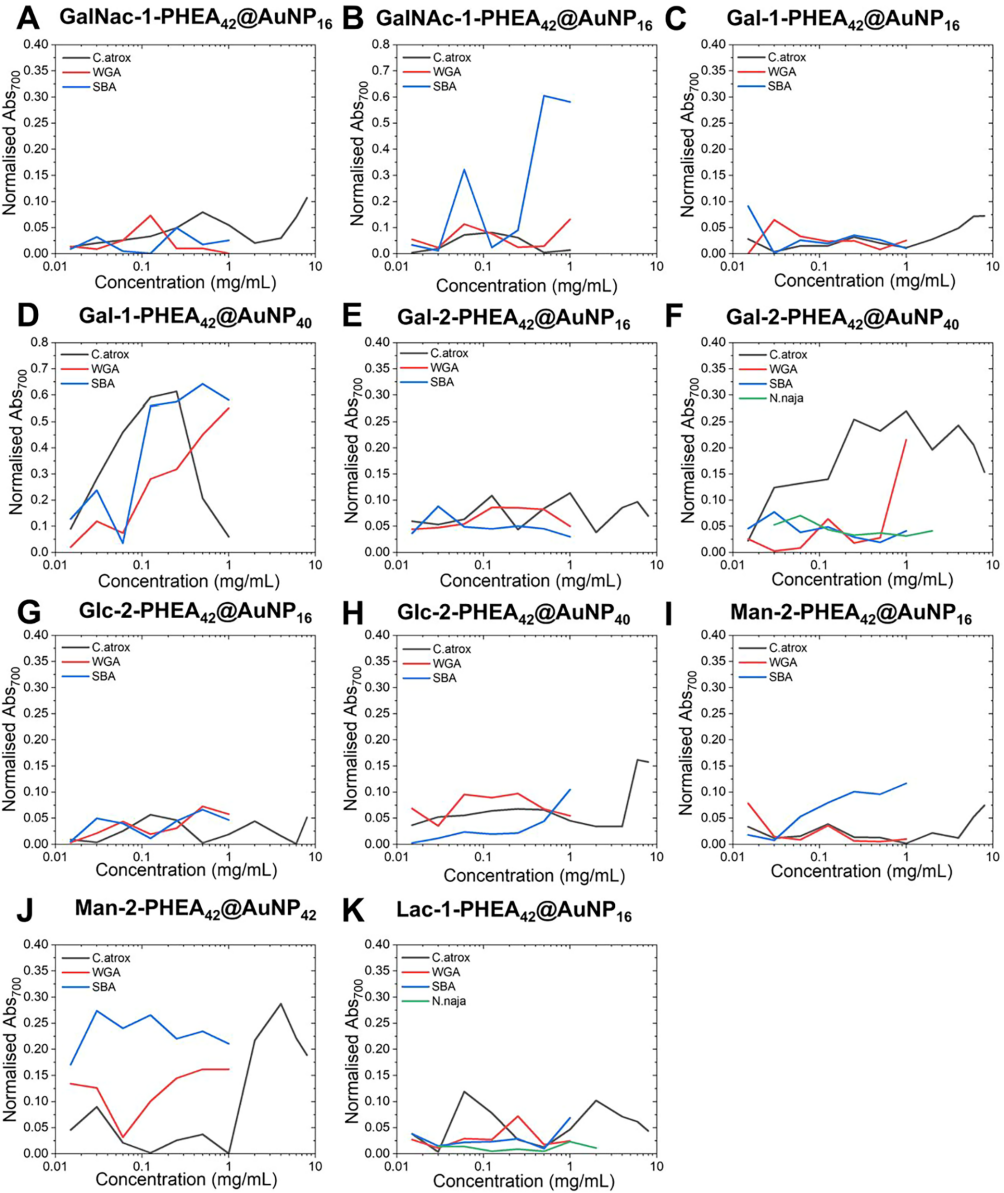
Normalized absorbance at 700 nm for round 1 particles versus SBA, WGA, C. atrox venom, and N. naja venom at varying concentrations. (A) GalNAc-1-PHEA_42_@AuNP_16_, (B) GalNAc-1-PHEA_42_@AuNP_40_, (C) Gal-1-PHEA_42_@AuNP_16_, (D) Gal-1-PHEA_42_@AuNP_40_, (E) Gal-2-PHEA_42_@AuNP_16_, (F) Gal-2-PHEA_42_@AuNP_40_, (G) Glc-2-PHEA_42_@AuNP_16_, (H) Glc-2-PHEA_42_@AuNP_40_, (I) Man-2-PHEA_42_@AuNP_16_, (J) Man-2-PHEA_42_@AuNP_40_, and (K) Lac-1-PHEA_42_@AuNP_16_.

From the initial round of testing (round 1), two systems displayed promise: Lac-1-pHEA_42_@AuNP_16_ (Figures 2F–2J, 3K, and S103–S106) and Gal-2-pHEA_42_@AuNP_40_ (Figures 2A–2E, 3F, and S78–S82). Both displayed binding toward the C. atrox venom, with Lac-1-pHEA_42_@AuNP_16_ displaying some affinity at low venom concentrations alongside affinity toward both SBA and WGA, while Gal-2-pHEA_42_@AuNP_40_ displayed little affinity toward SBA and some affinity toward WGA. The affinity for both systems toward C. atrox was expected as Gal residues have been reported to be specific for RSVL.^43,69^ These systems were therefore studied further, tuning the polymer length and AuNP size.

Gal-2-pHEA_42_@AuNP_40_ demonstrated an approximate limit of detection (LoD) for C. atrox versus the WGA and SBA controls at 15 and 20 μg·mL^–1^, respectively (Figures 2E and 3F). This is the point at which C. atrox binding cannot be discerned above the binding of WGA and SBA. Comparing this LoD to conventional diagnostic tools to detect snake venoms shows promise. Lión et al. developed a latex agglutination test to detect Crotalus spp. venoms with a LoD of 167 μg·mL^–1^, with an assay duration of 10 min, which is comparable to the aims of this study.^70^ However, other antibody-based immunoassays, such as ELISA, have LoDs from μg·mL^–1^ to ng·mL^–1^ with assay durations of minutes to overnight.^5^ Further improvement of the LoD is needed, however with pharmacokinetic studies suggesting a clinically relevant venom range of 1 - 1000 ng·mL^–1^ up to 50 h post viper-bite.^71^ While our proof-of-concept assay currently falls above this range, further refinement of the polymer, glycan, nanoparticle, buffer, etc., can be used to further improve the LoD.^49^

It is possible that there is an unexpected lack of binding of certain glycans (especially the terminal Gal residues) to C. atrox and SBA may be due to the size of the AuNP, tether chain length, or steric bulk near the glycan itself preventing access to the lectin binding site. Previous literature has reported that the size of AuNP is crucial to generating a robust signal under certain conditions, with a study by Micallef et al. demonstrating that 16 nm, 30 nm, and, to a lesser extent, 40 nm AuNPs were often too small to signal aggregation, notwithstanding that binding may still occur.^49,72,73^ Polymer tether lengths are also well known to impact aggregation outcomes; thus, a shorter polymer was utilized along with different-sized, commercially available AuNPs to produce a further library of systems with the Lac-1 and Gal-2 glycans.^34,49,72^ This included the inclusion of two new polymers, pHEA_25_ and pHEA_52_, and AuNP_30_. This synthetic flexibility is a key benefit of a glycopolymer system versus an antibody system.

The second round of testing was more varied and resulted in the following. Lac-1-pHEA_25_@AuNP_30_ demonstrated no affinity to SBA but displayed strong binding to WGA and a weak affinity to C. atrox at higher concentrations (Figures 4E and S108–S111). Lac-pHEA_25_@AuNP_16_ displayed little affinity to SBA but strong affinity to both WGA and C. atrox (Figures 4D and S99–S102). Gal-2-pHEA_25_@AuNP_30_ demonstrated a strong affinity to both WGA and C. atrox with little binding exhibited to SBA (Figures 4B and S70–S73). Gal-2-pHEA_25_@AuNP_16_ demonstrated moderate affinity to SBA and C. atrox (Figures 4A and S62–S65). Finally, Gal-2-pHEA_42_@AuNP_30_ displayed a moderate affinity to WGA, C. atrox, and SBA (Figures 4C and S74–S77). This may offer support that the architecture of SBA requires a larger AuNP to provide a robust signal as noted by Micallef et al.^49^ It is also notable that presentation of galactosamine on the polymer via an amide linkage closely mimics GalNAc, a glycan with affinity for WGA.^74^

**Figure 4:**
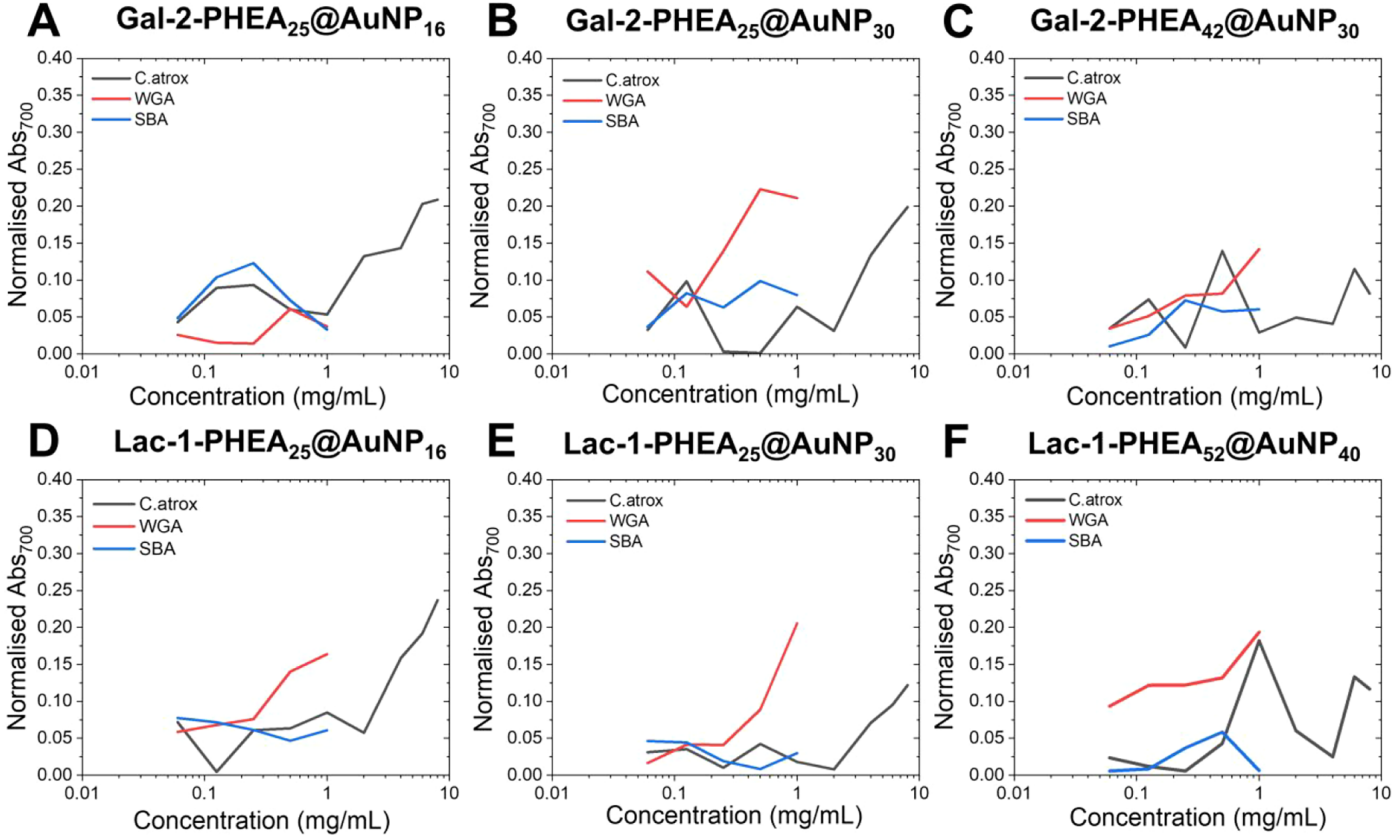
Normalized absorbance at 700 nm for round 2 and 3 particles versus SBA, WGA and C. atrox venom at varying concentrations. (A) Gal-2-PHEA_15_@AuNP_16_, (B) Gal-2-PHEA_25_@AuNP_30_, (C) Gal-2-PHEA_42_@AuNP_30_, (D) Lac-1-PHEA_25_@AuNP_16_, (E) Lac-1-PHEA_25_@AuNP_30_, and (K) Lac-1-PHEA_52_@AuNP_40_.

Partial or full aggregation was again observed by eye, this time with Lac-1-pHEA_42_@AuNP_30_, prior to the plasmonic assay testing, indicating an unstable system, likely due to similar reasons as Lac-1-pHEA_42_@AuNP_40_. To explore this further, a final system (round 3), Lac-1-pHEA_52_@AuNP_40_ was prepared (Figures 4F and S112–S115), to study the effect of a longer polymer tether on the system. The literature suggests that a longer polymer tether should provide increased colloidal stability to the system which it appears to have done.^75^ This system did not aggregate immediately and stabilizes the 40 nm AuNP, which the shorter polymer tethers were unable to do. Lac-1-pHEA_52_@AuNP_40_ also displayed a strong affinity to WGA and a moderate affinity to C. atrox, with no affinity to SBA. It was unexpected that a shorter polymer (Lac-1-pHEA_25_@AuNP_30_) allowed the glycosylated nanoparticle system to remain dispersed and did not aggregate like its longer counterpart (Lac-1-pHEA_42_@AuNP_30_). The literature suggests that longer polymers may provide more support to sustain the larger nanoparticles as opposed to shorter polymers.^34,49,72,76^ Our data supports the literature in this regard as, comparing Lac-1-pHEA_42_@AuNP_40_ and Lac-1-pHEA_52_@AuNP_40_, the former aggregated while the latter remained dispersed. This may indicate that the issue with pHEA_42_ may be due to grafting density and how the polymer chains are ‘grafted’ onto the AuNP.

To further explore the binding of the nanoparticle library, biolayer interferometry (BLI) studies were carried out using Lac-1-pHEA_42_AuNP_16_, Gal-2-pHEA_42_AuNP_40_, Man-2-pHEA_42_AuNP_16_, and Glc-2-pHEA_42_AuNP_16_ versus C. atrox venom and N. naja venom (Figures 5A, 5B and S131–S140). N. naja (Indian cobra) is a snake of the Elapidae family; its venom is well characterized and commercially available, notably it does not contain lectins compared to Viperidae venoms such as C. atrox.^44^ BLI is an optical biosensing technique that enables the label-free analysis of biomolecular interactions. Multiple protocols were explored, including immobilization of the nanoparticles on the probe and competition assays. While differences were observed in the association and dissociation profiles of C. atrox and N. naja venoms with the particle systems tested, there were no significant differences between particles with different sugars. Because the venom is a mixture of many components, the binding profile may be dominated by nonspecific interactions of proteins other than the lectin of interest. This is overcome in the gold nanoparticle systems by tuning the monomer used, polymer length, and gold size to avoid aggregation triggered by nonspecific protein binding.^49,73^

**Figure 5:**
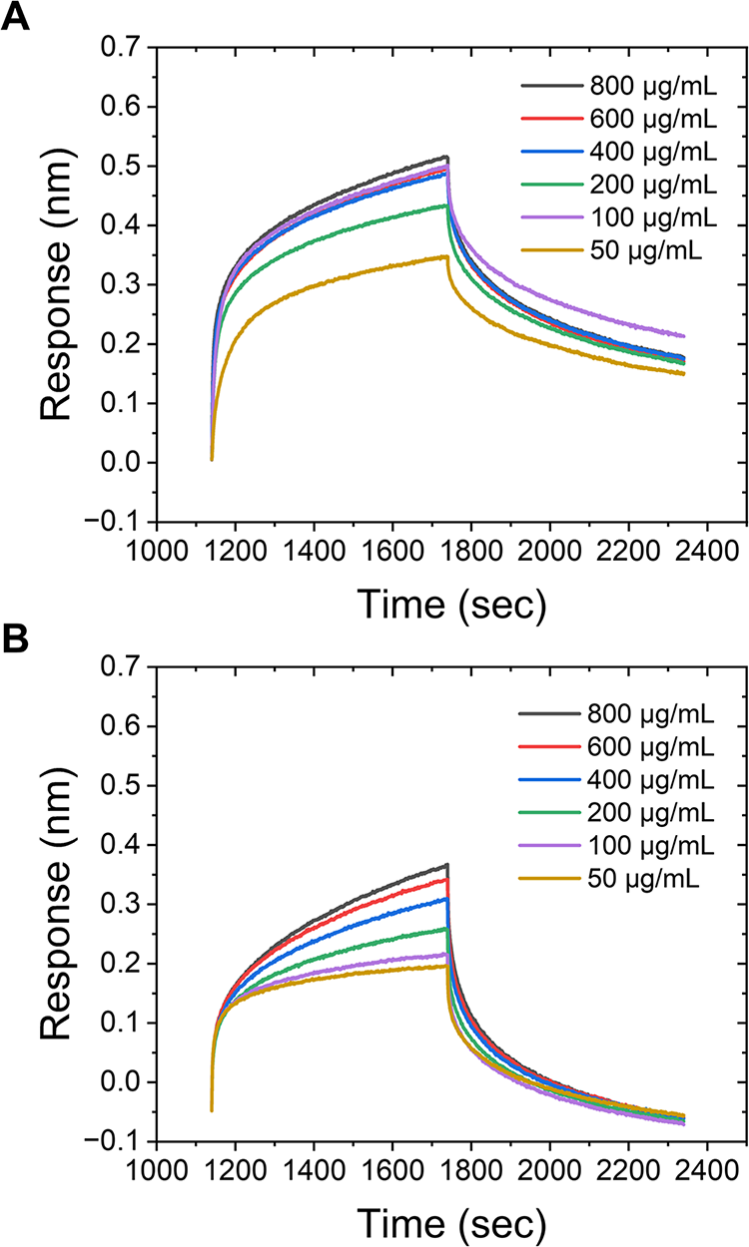
BLI analysis of Gal-2-pHEA_42_@AuNP_40_ aminopropylsilane sensors versus varying concentrations of analyte: (A) C. atrox venom and (B) N. naja venom.

Considering the above, the optimal UV–vis systems, Gal-2-pHEA_42_@AuNP_40_ (Figure 2D, 2E) and Lac-1-pHEA_42_@AuNP_16_ (Figure 2I, 2J), were tested against N. naja venom in the UV–vis assay. UV–vis assays were performed with N. naja venom did not show any significant changes as the concentration of venom increased, again indicating the selectivity of the UV–vis assay for C. atrox venom. This is a key advantage of our UV–vis assay approach that can differentiate N. naja venom from C. atrox venom despite the multiple components in venoms that impact BLI.

## Conclusions

In conclusion, a library of 19 glycosylated nanoparticles were synthesized with a range of varying polymer tether lengths, glycans, and AuNP sizes, of which two were successful at rapidly detecting C. atrox snake venom specifically using UV–vis assays against lectin controls, WGA and SBA, and a control Elapidae venom (N. naja). The effect of varying polymer chain length was also studied, with pHEA_52_ polymer being more effective than pHEA_42_ in stabilizing larger AuNPs, indicating the importance of careful control of both experimental and chemical parameters to ensure sensitive and specific aggregation.

The optimal systems for C. atrox venom detection were Gal-2-pHEA_42_@AuNP_40_ and, to a lesser extent, Lac-1-pHEA_42_@AuNP_16_. Therefore, this study supports the literature that Gal-terminating glycans show an affinity to C. atrox venom, although purification of RSVL will need to be undertaken to confirm binding to the C-type RSVL.43 Additionally, comparing the LoD of the Gal-2-pHEA_42_@AuNP_40_ assay to the previous literature validates the proof of concept, and its future feasibility as a diagnostic tool for C. atrox envenomation followed a further refinement of the glycan. Therefore, this study demonstrates that glycans can be used to sense snake venom in a prototype UV–vis assay using glycopolymer-functionalized gold nanoparticles.

## Supplementary Material



## Notes

Experimental details; characterization; UV–vis spectra; biolayer interferometry; XPS; and MALDI-MS (PDF)
