# Supplementary material for: Glycopolymer-Functionalized Gold Nanoparticles for the Detection of Western Diamondback Rattlesnake (Crotalus atrox) Venom
Source: Biomacromolecules. 2025 May 20;26(6):3514–24. doi: 10.1021/acs.biomac.5c00125 (PMC12152936; doi:10.1021/acs.biomac.5c00125)
Supplement: Supplementary file 1 [file bm5c00125_si_001.pdf]

Supplementary Information

Glycopolymer Functionalised Gold Nanoparticles for the Detection of Western Diamondback Rattlesnake (*C. atrox*) Venom

*Mahdi Hezwani,[a] Derecash Anokye,[a] Douglas E. Soutar,[a] Melissa Ligorio,[a] Neil Prabhakar,[a] Jack C. Oram,[a] Alexander Cantor,[a] Garrett D. Jackson,[a] Roberto Terracciano,[a] Marc Walker[b] and Alexander N. Baker[a]**

[a] Department of Chemistry, University of Warwick, Gibbet Hill Road, CV4 7AL, Coventry, UK

[b] Department of Physics, University of Warwick, Gibbet Hill Road, CV4 7AL, Coventry, UK

*<u>*Corresponding Author*</u>: <u>alexander.baker@warwick.ac.uk</u>

Contents

Contents..2

Physical and Analytical Methods..5

NMR Spectroscopy..5

Mass Spectrometry..5

FTIR Spectroscopy..5

Size Exclusion Chromatography..5

X-ray Photoelectron Spectroscopy (XPS)..6

Dynamic Light Scattering..6

UV-vis Spectroscopy..7

Transmission Electron Microscopy..7

Biolayer Interferometry..7

MALDI-MS..8

Materials..9

Synthetic Methods..10

Synthesis of 2-(dodecylthiocarbanothionylthio)-2-methyl propionic acid (DMP)....................10

Synthesis of pentafluorophenyl-2-dodecylthiocarbonothioylthio)-2-methylpropanoate (PFP-DMP)..11

Representative synthesis of poly(hydroxy ethyl acrylamide)....................................12

Synthesis of 2-Chloro-1,3-Dimethylimidazolidinium Hexafluorophosphate....................13

Synthesis of 1-deoxy-1-azido-β-D-N-acetyl-galactosamine....................................15

Synthesis of 1-deoxy-β-D-1-amino-N-acetyl-galactosamine 16

Synthesis of 1-deoxy-1-amino-galactose 17

Citrate-Stabilised 16 nm Gold Nanoparticle Synthesis 19

Additional Data and Figures 21

DLS, UV-vis and TEM Particle Characterisation 22

UV-vis Aggregation Assay Data 45

GalNAc-1-pHEA$_{42}$*@AuNP*$_{16}$ 45

GalNAc-1-pHEA$_{42}$*@AuNP*$_{40}$ 47

Gal-1-pHEA$_{42}$*@AuNP*$_{16}$ 49

Gal-1-pHEA$_{42}$*@AuNP*$_{40}$ 51

Gal-2-pHEA$_{25}$*@AuNP*$_{16}$ 53

Gal-2-pHEA$_{42}$@AuNP$_{16}$ 55

Gal-2-pHEA$_{25}$@AuNP$_{30}$ 57

Gal-2-pHEA$_{42}$*@AuNP*$_{30}$ 59

Gal-2-pHEA$_{42}$*@AuNP*$_{40}$ 61

Glc-2-pHEA$_{42}$*@AuNP*$_{16}$ 64

Glc-2-pHEA$_{42}$*@AuNP*$_{40}$ 66

Man-2-pHEA$_{42}$*@AuNP*$_{16}$ 68

Man-2-pHEA$_{42}$*@AuNP*$_{40}$ 70

Lac-1-pHEA$_{25}$*@AuNP*$_{16}$ 72

Lac-1-pHEA$_{42}$*@AuNP*$_{16}$ 74

Lac-1-pHEA$_{25}$@AuNP$_{30}$ 77

Lac-1-pHEA$_{52}$@AuNP$_{40}$ 79

Biolayer Interferometry Data 99

MALDI-MS 104

References 105

Physical and Analytical Methods

NMR Spectroscopy

^{1}H-NMR, ^{13}C-NMR and ^{19}F-NMR spectra were recorded at 300 MHz or 400 MHz on a Bruker Avance III HD 300 or Avance III HD 400 spectrometer respectively, with chloroform-*d* ($CDCl_3$) or deuterium oxide (D_2O) as the solvent at room temperature. Chemical shifts of protons are reported as δ in parts per million (ppm) and are relative to either $CDCl_3$ (7.26) or D_2O (4.79).

Mass Spectrometry

Electrospray ionisation mass spectrometry (ESI-MS) was performed using an Agilent 6130B single quadrupole mass spectrometer. M/Z values are reported in Daltons.

FTIR Spectroscopy

Fourier Transform-Infrared (FT-IR) spectroscopy measurements were carried out using an Agilent Cary 630 FT-IR spectrometer, in the range of 650 to 4000 cm^{-1}.

Size Exclusion Chromatography

Size exclusion chromatography (SEC) analysis was performed on an Agilent Infinity II MDS instrument equipped with differential refractive index (DRI), viscometry (VS), dual angle light scatter (LS) and variable wavelength UV detectors. The system was equipped with 2 x PLgel Mixed D columns (300 x 7.5 mm) and a PLgel 5 μm guard column. The mobile phase used was DMF (HPLC grade) containing 5 mM NH_4BF_4 at 50 ℃ at flow rate of 1.0 $mL.min^{-1}$. Poly(methyl methacrylate) (PMMA) standards (Agilent EasyVials) were used for calibration between 955,000 – 550 $g.mol^{-1}$. Analyte samples were filtered through a nylon membrane with 0.22 μm pore size before injection. Number average molecular weights (M_n), weight average

molecular weights (M_w) and dispersities ($Đ_M = M_w/M_n$) were determined by conventional calibration using Agilent GPC/SEC software.

X-ray Photoelectron Spectroscopy (XPS)

The samples were attached to electrically-conductive carbon tape, mounted on to a sample bar and loaded into a Kratos Axis Ultra DLD spectrometer which possesses a base pressure below 1 x 10^{-10} mbar. XPS measurements were performed in the main analysis chamber, with the sample being illuminated using a monochromated Al Kα x-ray source. The measurements were conducted at room temperature and at a take-off angle of 90° with respect to the surface parallel. The core level spectra were recorded using a pass energy of 20 eV (resolution approx. 0.4 eV), from an analysis area of 300 μm x 700 μm. The spectrometer work function and binding energy scale of the spectrometer were calibrated using the Fermi edge and $3d_{5/2}$ peak recorded from a polycrystalline Ag sample prior to the commencement of the experiments. In order to prevent surface charging the surface was flooded with a beam of low energy electrons throughout the experiment and this necessitated recalibration of the binding energy scale. To achieve this, the C-C/C-H component of the C 1*s* spectrum was referenced to 285.0 eV. The data was analysed in the CasaXPS package, using Shirley backgrounds and mixed Gaussian-Lorentzian (Voigt) lineshapes. For compositional analysis, the analyser transmission function has been determined using clean metallic foils to determine the detection efficiency across the full binding energy range.

Dynamic Light Scattering

Hydrodynamic diameters (D_h) and size distributions of particles were determined by dynamic light scattering (DLS) using a Malvern Zetasizer Nano ZS with a 4 mW He-Ne 633 nm laser module operating at 25 °C. Measurements were carried out at an angle of 173° (back scattering), and results were analysed using Malvern DTS 7.03 software. All determinations were repeated

5 times with at least 10 measurements recorded for each run. D_h values were calculated using the Stokes-Einstein equation where particles are assumed to be spherical.

UV-vis Spectroscopy

Absorbance measurements were recorded on an Agilent Cary 60 UV-Vis Spectrophotometer and on a BioTek Epoch microplate reader.

Transmission Electron Microscopy

Dry-state stained TEM imaging was performed on a JEOL JEM-2100Plus microscope operating at an acceleration voltage of 200 kV. All dry-state samples were diluted with deionized water and then deposited onto formvar-coated copper grids.

Biolayer Interferometry

BLI was performed using a ForteBio Octet Red96 (ForteBio, USA) with assays conducted in black 96-well half-area plates. Experiments were carried out at 30 °C with agitation at 1000 rpm. Aminopropylsilane (APS) biosensor tips (ForteBio, USA) were hydrated in milliQ H_2O for at least 10 minutes prior to use.

A stable baseline was established in HEPES buffer containing 0.15 M NaCl, 5 mM $CaCl_2$, and 10 mM HEPES (pH 7.4) for 1 minute. The biosensors were then functionalized by loading functionalized gold nanoparticles (AuNPs) at an optical density (OD) of 0.2 in milliQ H_2O for 5 minutes, followed by quenching with 10 mg/mL bovine serum albumin (BSA). A 1-minute equilibration (x3) in 10 mM HEPES buffer was performed to remove unbound nanoparticles and protein and establish a stable baseline.

Following AuNP immobilization, the binding association with C. atrox or N. naja venom was performed in the same HEPES buffer for 10 minutes, followed by a 10-minute dissociation step in the same buffer.

For the competition assay, the same conditions were applied, using a venom concentration of 0.4 mg/mL during the association step, with AuNP dilutions based on their optical densities (ODs): 0.2, 0.15, 0.1, 0.05, 0.025, and 0.01.

MALDI-MS

MALDI-MS was performed using a Bruker Autoflex Speed mass spectrometer with an MTP 384 ground steel target plate. Solutions of PHEA samples at approximately 4 mg/ml in water were prepared. A 1% TFA solution in water was prepared, then KTFA was dissolved in this solution at 4 mg/ml. Then a saturated solution of super-DHB was prepared in the KTFA/TFA solution. 0.5 μl super-DHB / KTFA/ TFA solution and 0.5 μl PHEA solution were mixed on the plate and dried under an extractor hood at room temperature.

Materials

All chemicals were used as supplied unless otherwise stated. *N*-Hydroxyethyl acrylamide (97%), 4,4'-azobis(4-cyanovaleric acid) (ACVA, 98%), 4-dimethylaminopyridine (DMAP, > 98%), mesitylene (reagent grade), triethylamine (> 99%), sodium citrate tribasic dihydrate (> 99%), gold(III) chloride trihydrate (99.9%), ammonia (reagent grade), ammonium carbonate (reagent grade), potassium phosphate tri basic (≥ 98%, reagent grade), potassium hexafluorophosphate (99.5%), deuterium oxide (D_2O, 99.9%), deuterated chloroform ($CDCl_3$, 99.8%), diethyl ether (≥ 99.8%, ACS reagent grade), methanol (≥ 99.8%, ACS reagent grade), toluene (≥ 99.7%,), HEPES, carbon disulphide (≥ 99.8%), acetone (≥ 99%), 1-dodecane thiol (≥ 98%), lactose (reagent grade), Snake venom from *Crotalus atrox* (Western Diamondback Rattlesnake), potassium trifluoroacetate (98%), super-DHB (≥ 99%,) and pentafluorophenol (≥ 99%, reagent plus) were purchased from Sigma-Aldrich. DMF (> 99%), 2-bromo-2-methyl propionic acid (98%) were purchased from Acros Organics. Galactosamine HCl, glucosamine HCl, mannosamine HCl, 1-amino-1-deoxy-*N*-acetyl-galactosamine and 1-Ethyl-3-(3-dimethylaminopropyl)carbodiimide hydrochloride (EDCI, > 98%) were purchased from Carbosynth. HPLC grade acetonitrile (≥ 99.8%), glucose (lab-reagent grade), hexane fraction from petrol (lab reagent grade), DCM (99% lab reagent grade), sodium hydrogen carbonate (≥ 99%), ethyl acetate (≥ 99.7%, analytical reagent grade), sodium chloride (≥ 99.5%), calcium chloride, 40-60 petroleum ether (lab reagent grade), hydrochloric acid (~37%, analytical grade), glacial acetic acid (analytical grade) and magnesium sulphate (reagent grade) were purchased from Thermo Fisher Scientific. Soybean agglutinin (SBA) and wheat germ agglutinin (WGA) were purchased from Vector Laboratories. *Naja naja* (Indian cobra) venom was purchased from Latoxan. Trifluoroacetic acid 99.9% was purchased from ABCR. Ultra-pure water used for buffers was MilliQ grade 18.2 MΩ resistance.

Synthetic Methods

Synthesis of 2-(dodecylthiocarbanothionylthio)-2-methyl propionic acid (DMP)

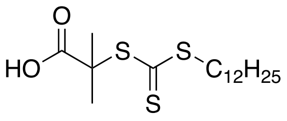

This was synthesised according to a previously published procedure.[1] 2.00 g (9.88 mmol) of 1-dodecane thiol was added dropwise to stirring 2.10 g (9.89 mmol) of K_3PO_4 in 30mL of acetone at RTP. The mixture was left to stir for 25 minutes to form a white suspension. 2.05 g (26.93 mmol) of carbon disulfide was then added and left for 10 minutes forming a yellow solution. 1.5 g (8.98 mmol) of 2-bromo-2-methyl-propionic acid was then added and the solution was left to stir for 16 hours. The solvent was removed under vacuum before the crude product was dissolved in 100 mL of 1M HCl and extracted with DCM (2 × 100 mL). The organic layer was washed with 200 mL water and 200 mL brine. The organic layer was dried with $MgSO_4$ and filtered under gravity. The solvent was then removed from the filtrate under vacuum. The crude product was purified using a silica column (60 - 80 PET : DCM : glacial acetic acid 75 : 24 : 1) and recrystalised in n-hexane to give a yellow solid (76%). δ_H (300 MHz, $CDCl_3$) 3.28 (2H, t, *J* 7.5, $SC\mathbf{H}_2CH_2$), 1.80 - 1.45 (8H, m, $C(CH_3)_2$ and $SCH_2C\mathbf{H}_2$), 1.45 - 1.2 (18H, m, $(C\mathbf{H}_2)_9CH_3$), 0.87 (3H, t, *J* 6.0, CH_3). δ_C (400 MHz, $CDCl_3$) 221.0 (1C, SC(S)S), 178.3 (1C, C(O)), 55.7 (1C, $\mathit{C}(CH_3)_2$), 37.7 (1C, SCH_2), 32.1 - 28.0 (9C, $SCH_2(\mathit{C}H_2)_9$), 25.4 (2C, $C(\mathit{C}H_3)_2$), 22.8 (1C, $\mathit{C}H_2CH_3$), 14.3 (1C, $CH_2\mathit{C}H_3$). m/z calculated as 364.16; found for ESI $[M+H]^+$ 365.3 and $[M+Na]^+$ 387.3. FTIR (cm^{-1}) – 2956, 2916.6 & 2850 (methyl and methylene), 1702 (ester C=O), 1459, 1437 & 1413 (methyl and methylene), 1280 ($C(CH_3)_2$), 1064 (S-C(S)-S).

Synthesis of pentafluorophenyl-2-dodecylthiocarbonothioylthio)-2-methylpropanoate (PFP-DMP)

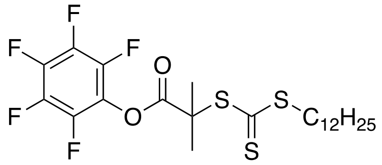

This was synthesised according to a previously published procedure.[1] 4.06 g (11.13 mmol) of DMP, 3.65 g (19.04 mmol) of EDC and 2.30 g (18.82 mmol) of DMAP were dissolved in 160 mL of DCM and degassed for 30 minutes. 7.28 g (39.55 mmol) of pentafluorophenol was added in 20 mL of DCM and the mixture stirred for 18 hours at RTP. The organic layer was washed with 3 M HCl (200 mL), 1 M $NaHCO_3$ (200 mL) and 0.5 M NaCl (200 mL). The organic layer was dried with $MgSO_4$ and filtered under gravity. The solvent was then removed from the filtrate under vacuum. The crude product was recrystallised in ethyl acetate (or hexane) overnight at -8°C and dried to give yellow crystals (90.9%). δ_H (300 MHz, $CDCl_3$) 3.31 (2H, t, *J* 7.5, SC**H**$_2$CH$_2$), 1.86 (6H, s, C(CH$_3$)$_2$), 1.69 (2H, qn, *J* 7.5, SCH$_2$C**H**$_2$), 1.48 - 1.16 (18H, m, (C**H**$_2$)$_9$CH$_3$), 0.94 - 0.82 (3H, m, CH$_3$). δ_C (75 MHz, $CDCl_3$) 220.1 (1C, SC(S)S), 169.7 (1C, C(O)), 143.1 (2C, meta C), 139.8 (1C, ipso C), 139.6 (1C, para C), 136.3 (2C, Ortho C), 55.5 (1C, *C*(CH$_3$)$_2$), 37.3 (1C, SCH$_2$), 32.0 - 22.8 (10C, SCH$_2$(*C*H$_2$)$_{10}$), 25.4 (2C, C(*C*H$_3$)$_2$), 14.1 (1C, CH$_2$*C*H$_3$). δ_F (300 MHz, $CDCl_3$) -151.44 - -151.61 (2F, m, OCC$_2$*H*$_2$C$_2$H$_2$CH), -148.50 (1F, t, *J* 21.5, OCC$_2$H$_2$C$_2$H$_2$C*H*), -162.23 - -162.47 (2F, m, OCC$_2$H$_2$C$_2$*H*$_2$CH). m/z calculated as 530.14; found for ESI $[M+Na]^+$ 553.3 and $[M+CH_3CN+Na]^+$ 593.5. FTIR (cm^{-1}) – 2956, 2917 & 2850 (methyl and methylene), 1702 (ester C=O), 1519 (aromatic C=C or C-F), 1460, 1437 & 1413 (methyl and methylene), 1280 (C(CH$_3$)$_2$), 1068 (S-C(S)-S).

Representative synthesis of poly(hydroxy ethyl acrylamide)

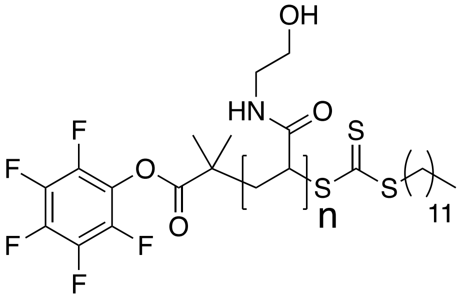

PFP-DMP (367 mg, 0.86 mmol), which was synthesised in an earlier step, was dissolved in a 50 : 50 mixture of toluene : methanol (22 mL) before adding the monomer 2-hydroxyethyl-acrylamide (2.05 g, 17.8 mmol) and 4,4-azobis (4-cyanovaleric acid) (40 mg, 0.26 mmol) under an atmosphere of nitrogen for 30 minutes. The mixture was then heated for two hours at 70 ℃ before polymerisation was terminated by immersing in a bath of liquid nitrogen. The solvent was then removed under reduced pressure. The polymer was then dissolved with minimal methanol before precipitation with diethyl ether and centrifuged at 13,000 rpm (18900 × g) for 10 minutes. The supernatant was decanted, and this process was repeated a further two times before drying the product at low pressure for 16 hours to afford at light yellow solid.

$pHEA_{52}$ - δ_H (400 MHz, D_2O) 8.44-7.87 (20H, s, N**H**), 3.76 - 3.57 (90H, s, NHC$\mathbf{H}_2$), 3.44 - 3.00 (80H, m, CH_2OH and methanol), 2.48 – 1.46, (144H, m, C**H**CONH and C$\mathbf{H}_2$CHCONH), 1.46 – 1.24 (30H, C(C$\mathbf{H}_3$)$_2$ SCH_2(C$\mathbf{H}_2$)$_{10}$) and 0.81 (3H, s, $(CH_2)_{10}$C$\mathbf{H}_3$).

$pHEA_{42}$ - δ_H (400 MHz, D_2O) 8.44-7.87 (10H, s, N**H**), 3.98 – 3.57 (48H, s, NHC$\mathbf{H}_2$), 3.56 – 3.00 (51H, s, C$\mathbf{H}_2$OH), 2.47 – 1.46, (70H, m, C**H**CONH and C$\mathbf{H}_2$CHCONH), 1.46 – 1.24 (27H, C(C$\mathbf{H}_3$)$_2$ SCH_2(C$\mathbf{H}_2$)$_{10}$) and 0.81 (3H, s, $(CH_2)_{10}$C$\mathbf{H}_3$). FTIR: 3286.5 (Broad, OH), 3099.7, 2930.1 (C(O)NH and NH), 1648.2, 1580.3 (C(O)NH).

pHEA$_{25}$ - δ_H (400 MHz, D_2O) 8.44-7.87 (5H, s, N**H**), 3.98 – 3.57 (21H, s, NHC$\mathbf{H}_2$), 3.56 – 3.00 (m, C$\mathbf{H}_2$OH and methanol), 2.50 – 1.0 (50H, m, C**H**CONH, C$\mathbf{H}_2$CHCONH and SCH$_2$(C$\mathbf{H}_2$)$_{10}$), 0.81 (3H, s, (CH$_2$)$_{10}$C$\mathbf{H}_3$).

Synthesis of 2-Chloro-1,3-Dimethylimidazolidinium Hexafluorophosphate

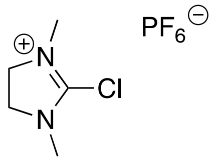

2-Chloro-1,3-dimethylimidazolidinium chloride (5.03 g, 29.8 mmol) was dissolved in anhydrous acetonitrile (100 mL) and stirred under an inert atmosphere before sodium hexafluorphosphate (4.99 g, 29.7 mmol) was added to the mixture and further stirred for 30 minutes. Upon completion, the solution was suction filtered through Celite and washed with acetonitrile before the solvent was removed under reduced pressure. This solid was then dissolved with the minimal amount of acetonitrile then precipitated with diethyl ether to form the product, a white solid, which was collected via suction filtration (6.56 g, 79 %).

δ_H (300MHz, DMSO) 3.25 (6H, s, 2x NC$\mathbf{H_3}$), 2.65 (4H, s, 2x NC$\mathbf{H_2}$).

Synthesis of 2-Azido-1-3-Dimethylimidazolinium Hexafluorophosphate (ADMP)

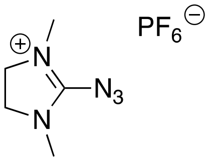

2-Chloro-1,3-dimethylimidizolidinium hexafluorophosphate (4.99 g, 17.9 mmol) was prepared in an earlier step and dissolved in anhydrous acetonitrile (60 mL) and stirred at 0°C under N2 atmosphere before adding sodium azide (1.67 g, 25.7 mmol) to the reaction mixture for 3 hours. The mixture was then filtered via suction filtration through Celite and washed with acetonitrile before the solvent was removed under reduced pressure. The resulting solid was dissolved in the minimum amount of acetonitrile before adding diethyl ether until precipitation. The precipitate was then dried under vacuum for 16 hours to obtain a crude product which recrystallised from acetone: toluene (1:1) to afford a white solid (4.00 g, 78 %).

δ_H (400 MHz, CD_3CN) 3.87 (4H, s, 2x NC$\mathbf{H_2}$), 3.08 (6H, s, 2x NC$\mathbf{H_3}$).

Synthesis of 1-deoxy-1-azido-β-D-N-acetyl-galactosamine

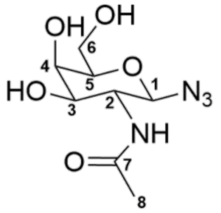

N-Acetyl-Galactosamine (100 mg, 0.292 mmol) was dissolved in 4:1 Deuterium Oxide: Acetonitrile (2ml) under nitrogen before being cooled to 0°C and subsequently adding triethylamine (205µL, 1.46 mmol) and stirring for a further 10 minutes maintaining temperature. ADMP (238 mg, 0.88 mmol) was then added to the reaction and stirred for a further 3 hours at 0°C before being left at 4°C for 16 hours. Upon completion, water was added (15mL) for dilution before being washed three times with dichloromethane (3 x 20mL) with the aqueous phase passed through a column of Amberlite® IR120 (which was previously treated with 1M NaOH). Finally, upon lyophilisation a white solid was obtained which was used directly in the next reaction.

δ_H (400 MHz, D_2O) 4.69 (1H, d, *J* 9.3, C-1H), 4.00 (1H, s, C-4H), 3.95 (1H, t, *J* 10.1, C-2H), 3.88 - 3.79 (4H, m, C-3H, C-5H and C-6H), 2.08 (3H, s, C-8H). δ_C (75 MHz, D_2O) 175.0 (C-7), 89.1 (C-1), 77.2 (C-5), 70.8 (C-3), 67.7 (C-4), 60.9 (C-6), 51.7 (C-2), 22.2 (C-8). m/z calculated at 246.21; found for ESI $[M+Na]^+$ 269.21 and $[M-H]^-$ 245.3. FTIR (cm^{-1}) –3180 (hydroxyl), 2150 (Azide), 1770, (C=O) 1100, (C-C).

Synthesis of 1-deoxy-β-D-1-amino-N-acetyl-galactosamine

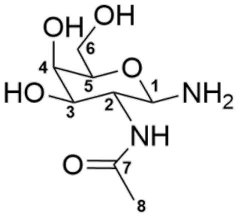

1-deoxy-1-azido-*β*-D-N-acetyl-galactosamine (137 mg, 0.555 mmol) was dissolved in methanol (5mL) under nitrogen along with $Pd(OH)_2/C$ (20 wt%, 39 mg, 0.055 mmol). Hydrazine hydrate (50 %, 100 µ*L*, 1.39 mmol) was then directly injected into the mixture before refluxed at 60°C for 16 hours. The reaction mixture was then cooled, filtered and the solvent was then removed under reduced pressure to give the product as a white solid. δ_H (400 MHz, D_2O) 4.66 (1H, d, *J* 8.2, C-1H), 4.12 (1H, d, *J* 5.6, C-4H), 3.91 - 3.89 (m, C-2H), 3.78 - 3.75 (3H, m, C-5H and C-6H), 3.72 - 3.68 (1H, m, C-3H), 2.06 (3H, s, C-8H). δ_C (75 MHz, D_2O) 174.7 (C-7), 95.4 (C-1), 75.2 (C-3), 71.1 (C-5), 70.5 (C-4), 67.8 (C-2), 61.2 (C-6), 21.9 (C-8). m/z calculated at 220.21; found for ESI $[M+Na]^+$ 243.1 and $[M-H]^-$ 219.12. FTIR (cm-1) – 3380 & 3345 (amine), 3080 (hydroxyl), 1770, (C=O) 1100, (C-C).

Synthesis of 1-deoxy-1-amino-galactose

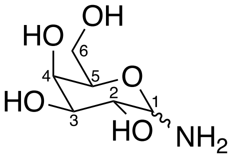

This was synthesised according to previously published procedures.[2,3] 0.36 g (2 mmol) of galactose and 0.158 g (2 mmol) ammonium hydrogen carbonate were added to a 10 mL solution of 16 mol.dm^{-3} ammonia solution. The mixture was heated for 16 hours at 42 ℃. The liquid was removed under vacuum and resuspended in 5mL of water. The solution was lyophilised to give a cream or off-white crystalline solid, that did not smell of ammonia. (94.6%) (α:β 0.71:0.38). δ_H (300 MHz, $CDCl_3$) 4.24 (0.38H, d, *J* 8.81, C-1H$^{\alpha}$), 4.03 (0.71H, d, *J* 8.75, C-1H$^{\beta}$), 3.93 (1H, d, *J* 3.00, C-3H or C-4H), 3.90-3.45 (8H, m, C-3H or C-4H, C-6H_2, C-5H and hydroxyls), 3.39 (1H, t, *J* 9.0, C-2H). δ_C (75 MHz, $CDCl_3$) 87.6 (C-1H$^{\alpha}$), 85.5 (C-1H$^{\beta}$), 76.0 (C-5), 73.4 (C-3 or C-4), 72.0 (C-2), 68.96 (C-3 or C-4), 61.1 (C-6). m/z calculated as 179.171; found for ESI $[2M+Na]^+$ 381.3 and $[M-H^+]^-$ 178.1. FTIR (cm^{-1}) – 3650 - 2500 (hydroxyl), 2924 & 2878 (alkane), 1646 & 1584 (amine), 1465 & 1420 (hydroxyl).

Synthesis of 1-deoxy-β-D-1-amino-lactose

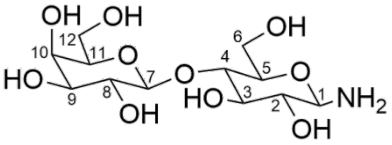

Lactose (674 mg, 2 mmol), ammonium bicarbonate (158 mg, 2 mmol) and ammonia solution (10 mL, 16 mol dm^{-3}) were combined and heated for 16 hours at 42°C. The solvent was removed under reduced pressure before being resuspended with 5 mL of water and lyophilised to give a white crystalline solid. ESI $[M+Na]^+$ 364.1 and $[M-H]^-$ 340.12 δ_H (400 MHz, D_2O) 4.77 (1H, s(br), C-1H), 4.46 (1H, d, *J* 7.7, C-7H), 4.01 - 3.93 (2H, m, C-4H and C-12H), 3.86 - 3.71 (5H, m, C-6H, C-10H, C-11H and C-12H), 3.69 - 3.63 (3H, m, C-3H, C-4H and C-9H),

3.57 - 3.53 (2H, m, C-2H and C-8H). δ_C (75 MHz, D_2O) 102.9 (C-7), 89.9 (C-1), 78.6 (C-3), 77.7 (C-11), 76.7 (C-9), 75.7 (C-10), 75.3 (C-5), 72.5 (C-2), 70.9 (C-8), 68.5 (C-4), 61.0 (C-6), 60.2 (C-12).

Representative Glycan functionalisation of pHEA$_{42}$ using 1-deoxy-1-amino-galactose

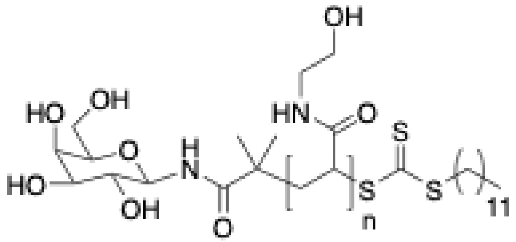

PHEA (200 mg, 0.039 mmol) was dissolved in DMF (20 mL) along with the glycan (1-amino-1-deoxy-galactose as representative) (0.078 mmol) and TEA (0.05 M). The reaction was subsequently stirred for 16 hours at 50℃ before the solvent was removed under pressure. The crude product was then dissolved in the minimal amount of methanol before precipitation with cold with diethyl ether. The mixture was centrifuged for two minutes at 13000 (18900 ×g) and the supernatant decanted off. The product was then dissolved again with the minimal amount of methanol before removing the solvent under reduced pressure to give an orange/ brown crystalline solid. The loss of the fluorine signal in ^{19}F NMR was used to indicate the reaction had gone to completion.

δ_H (300 MHz, D_2O) 8.41 - 7.79 (25H, m, N*H*), 4.03 - 3.55 (~90*H*, m, NHCH_2 & glycan protons), 3.57 - 2.95 (~82*H*, m, CH_2OH & SCH_2 & glycan protons), 2.30 - 1.85 (50*H*, m, CH_2C*H*C(O), C(CH_3)$_2$ & glycan protons), 1.85 - 0.92 (110H, m, CH_2C*H*C(O) & $CH_2CH_2CH_2CH_2CH_2CH_2CH_2CH_2CH_2CH_3$ & glycan protons), 0.81 - 0.71 (5*H*, m, CH_2CH_3).

Citrate-Stabilised 16 nm Gold Nanoparticle Synthesis

This was synthesized according to a previously published procedure. [4] To 500 mL of water was added 0.163 g (0.414 mmol) of gold(III) chloride trihydrate, the mixture was heated to reflux and 14.6 mL of water containing 0.429 g (1.46 mmol) of sodium citrate tribasic dihydrate was added. The reaction was allowed to reflux for 30 minutes before cooling to room temperature over 3 hours. The solution was centrifuged at 13 krpm (18900 ×g) for 30 minutes and the pellet resuspended in 40 mL of water to give an absorbance at 520 nm of ~1 Abs.

16 nm Gold Nanoparticle Functionalisation

5 mg of each glycopolymer was agitated overnight with 2.5 mL of 16nm AuNPs at 1Abs at UV_{MAX}. The solution was then centrifuged at 12,500 rpm (9800 ×g) for thirty minutes before the pellet was resuspended in 2mL of water. This washing was repeated a further two times before all the nanoparticles were concentrated to OD 2 by dilution of the pellet with 50µL of water at a time and measuring the UV_{MAX}.

30 nm & 40 nm Gold Nanoparticle Functionalisation

5 mg of each glycopolymer was agitated overnight with 2.5 mL of the AuNPs at 1Abs at UV_{MAX}. The solution was then centrifuged at 8,000 rpm (4200 ×g) for thirty minutes before the pellet was resuspended in 2 mL of water. This washing was repeated a further two times before before all the nanoparticles were concentrated to OD 2 by dilution of the pellet with 50 µL of water at a time and measuring the UV_{MAX}.

4× HEPES Buffer Preparation

0.952 g of HEPES (40 mmol.dm^{-3}), 3.51 g of sodium chloride (0.6 mmol.dm^{-3}) and 0.0044 g of calcium chloride (0.4 mmol.dm^{-3}) and 0.53 mg of manganese chloride (0.042 mmol.dm^{-3}) were dissolved in 100 mL of distilled water and stirred for thirty minutes to yield a 4× concentrated buffer.

Aggregation Assay Protocol with varying Lectin Concentrations and Fixed AuNP OD

An aggregation assay was prepared in a 96 well plate by addition of 12.5 μL of functionalised gold nanoparticles. Then, a serial dilution of 50 μL of 2 mg.mL^{-1} lectin (SBA, WGA, *C. atrox* venom), diluting with milli-Q water, to attain 25 μL aliquots of concentrations 8, 6,4, 2, 1, 0.5, 0.25, 0.125, 0.063 and 0.031 mg.mL^{-1} was added. Finally, 12.5 μL of the HEPES buffer was added to give a total volume in the well of 50 μL. This was then analysed by UV-Vis in a range of 400 – 700 nm after incubation at RTP (~20 °C) for 20 minutes.

$$|Normalised\ Abs_{700}| = Abs_{700}(x\ mg.ml^{-1}) - Abs_{700}(0\ mg.ml^{-1})$$

Equation 1 – Equation for determining normalised Abs_{700} from UV-vis data

Additional Data and Figures

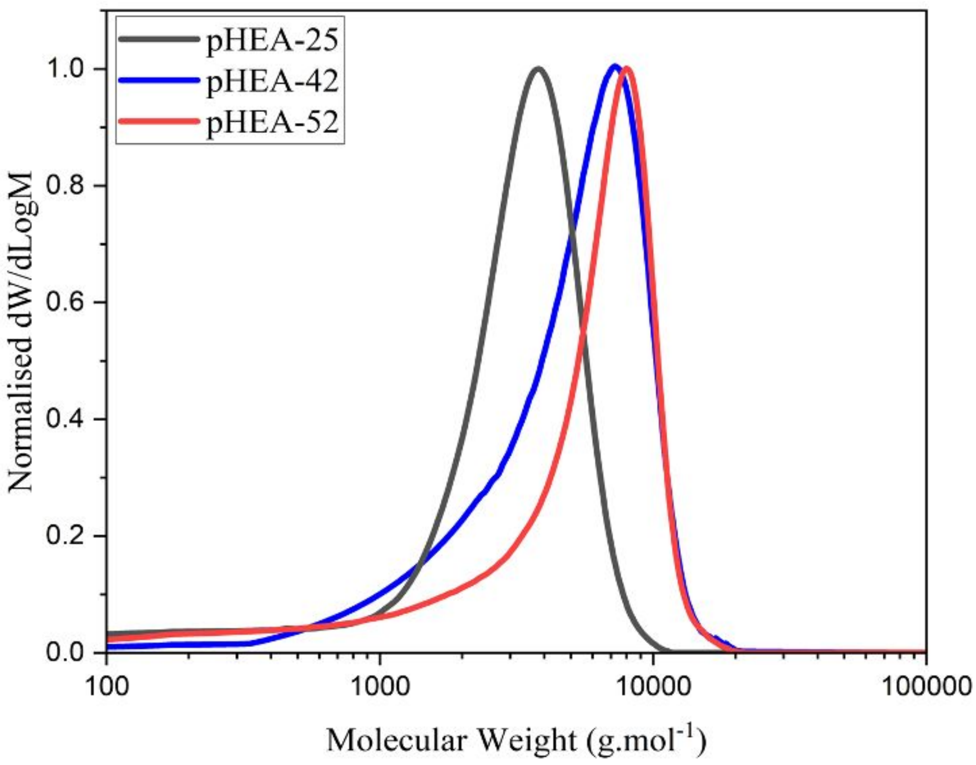

Figure S1 – Normalised size exclusion chromatography RI molecular weight distributions of telechelic PHEA obtained in DMF versus PMMA standards.

DLS, UV-vis and TEM Particle Characterisation

Table S1 – Nanoparticles Synthesised and Characterisation

**Particle**	**λ_{SPR}[a] (nm)**	**DLS[b] (nm)**
$AuNP_{16}$	517	16.2 ±0.1
$AuNP_{30}$	525	29.7 ±0.5
$AuNP_{40}$	530	40.2 ±0.1
GalNAc-1-$pHEA_{42}$@$AuNP_{16}$	520	27.2 ±1.1
GalNAc-1-$pHEA_{42}$@$AuNP_{40}$	531	38.2 ±1.1
Gal-1-$pHEA_{42}$@$AuNP_{16}$	520	24.2 ±1.1
Gal-1-$pHEA_{42}$@$AuNP_{40}$	532	41.2 ±1.3
Gal-2-$pHEA_{25}$@$AuNP_{16}$	525	27.7 ±1.8
Gal-2-$pHEA_{42}$@$AuNP_{16}$	520	28.2 ±0.6
Gal-2-$pHEA_{25}$@$AuNP_{30}$	526	31.1 ±0.1
Gal-2-$pHEA_{42}$@$AuNP_{30}$	528	32.2 ±1.1
Gal-2-$pHEA_{42}$@$AuNP_{40}$	531	42.3 ±1.4
Glc-2-$pHEA_{42}$@$AuNP_{16}$	519	26.3 ±1.3
Glc-2-$pHEA_{42}$@$AuNP_{40}$	533	40.2 ±0.3
Man-2-$pHEA_{42}$@$AuNP_{16}$	519	25.0 ±0.5
Man-2-$pHEA_{42}$@$AuNP_{40}$	532	38.9 ±0.6
Lac-1-$pHEA_{25}$@$AuNP_{16}$	525	25.2 ±0.3
Lac-1-$pHEA_{42}$@$AuNP_{16}$	520	24.2 ±0.9
Lac-1-$pHEA_{25}$@$AuNP_{30}$	527	30.1 ±1.1
Lac-1-$pHEA_{42}$@$AuNP_{30}$	Unstable	Unstable
Lac-1-$pHEA_{42}$@$AuNP_{40}$	Unstable	Unstable
Lac-1-$pHEA_{52}$@$AuNP_{40}$	532	38.2 ±0.4

[a]Maximum absorption wavelength from the surface plasmon resonance band of the particles and characteristic ratio. [b]Diameter from dynamic light scattering ± standard error from three measurements.

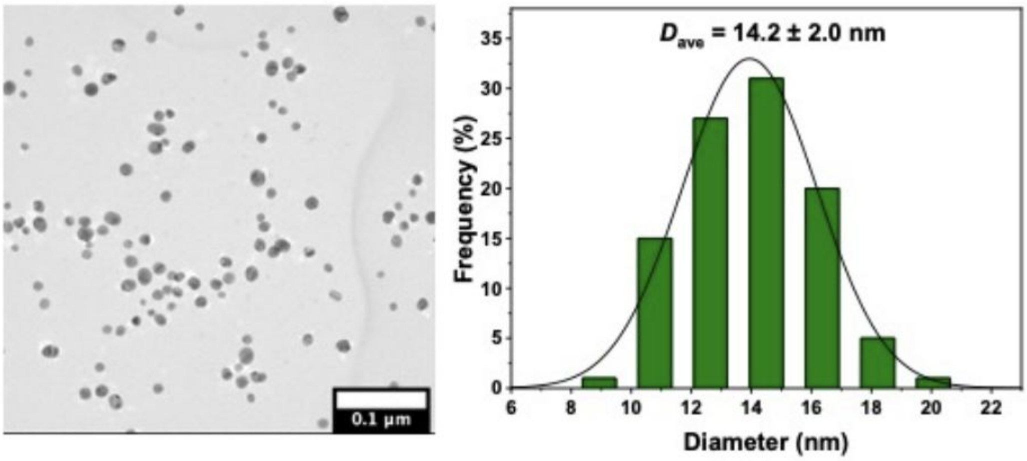

Figure S2 – TEM images (Left) and histograms (right) of citrate stabilized 16nm AuNPs.

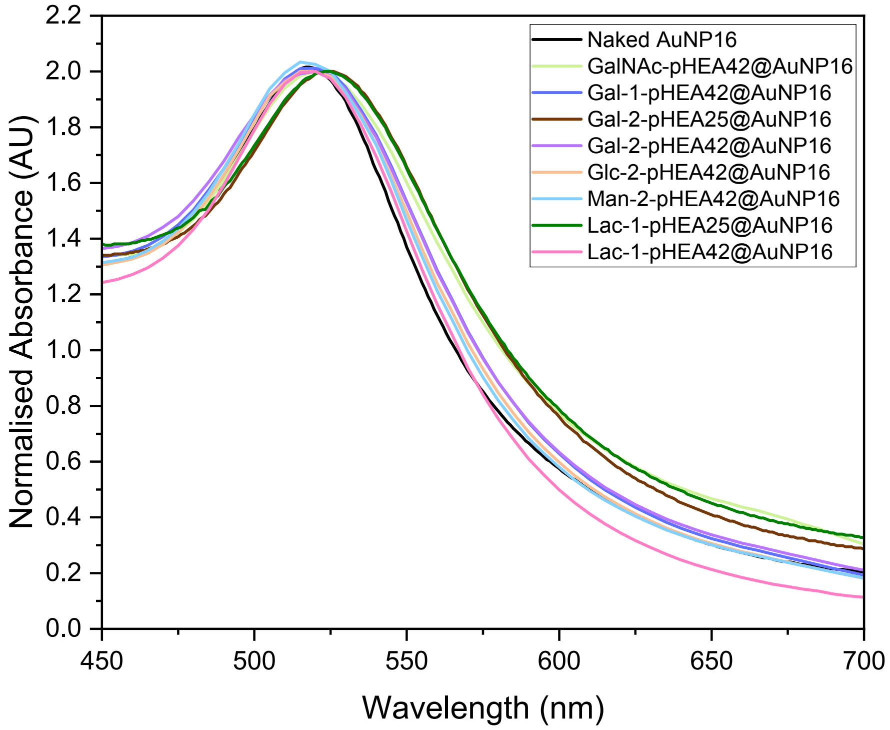

Figure S3 – UV-vis spectra of 16 nm AuNPs

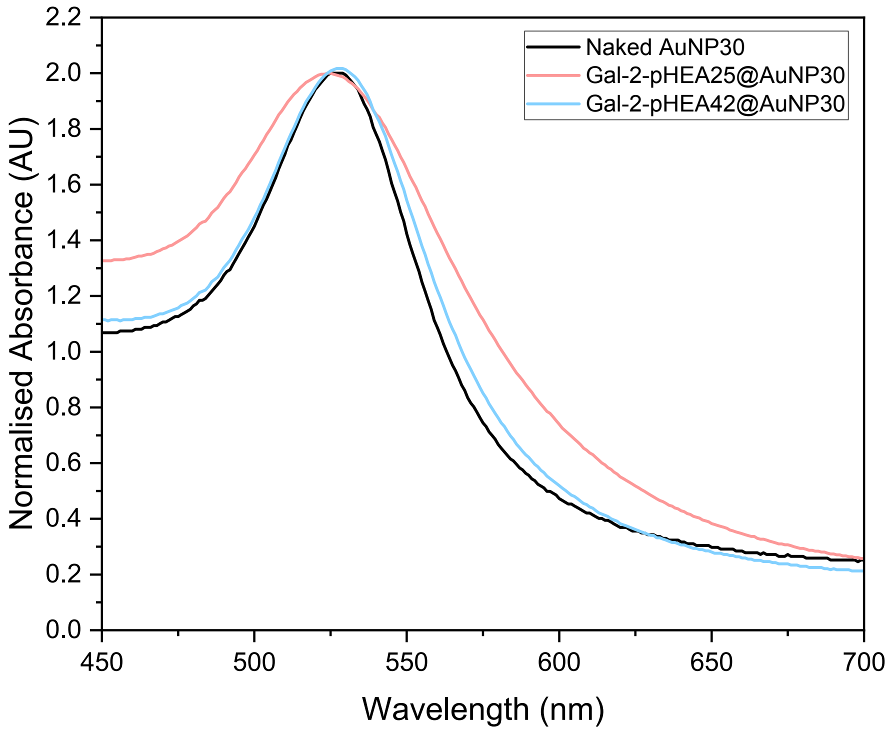

Figure S4 – UV-vis spectra of 30 nm AuNPs

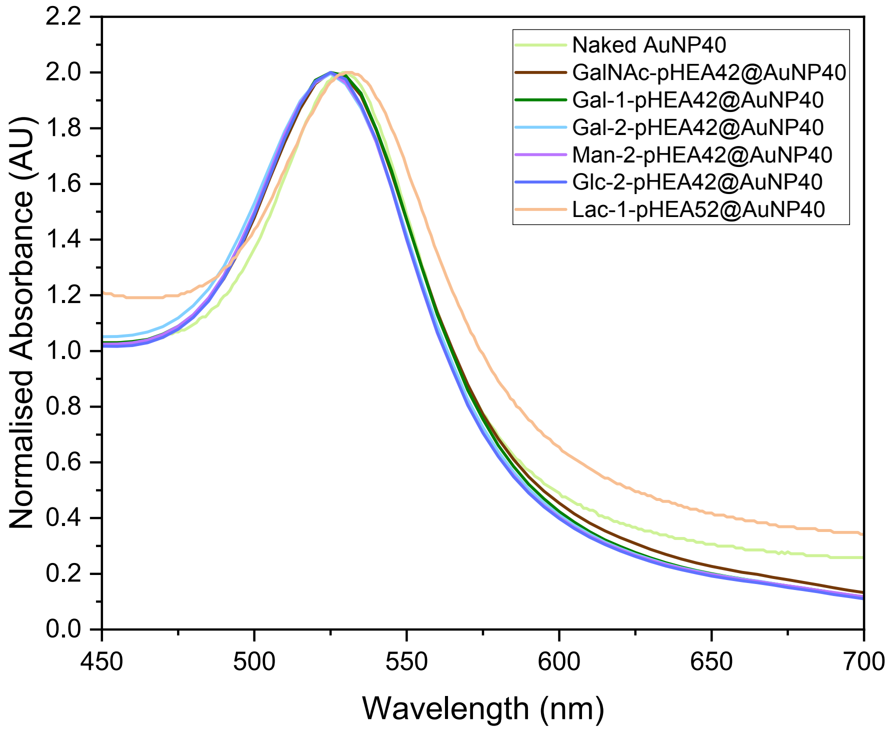

Figure S5 – UV-vis spectra of 40 nm AuNPs

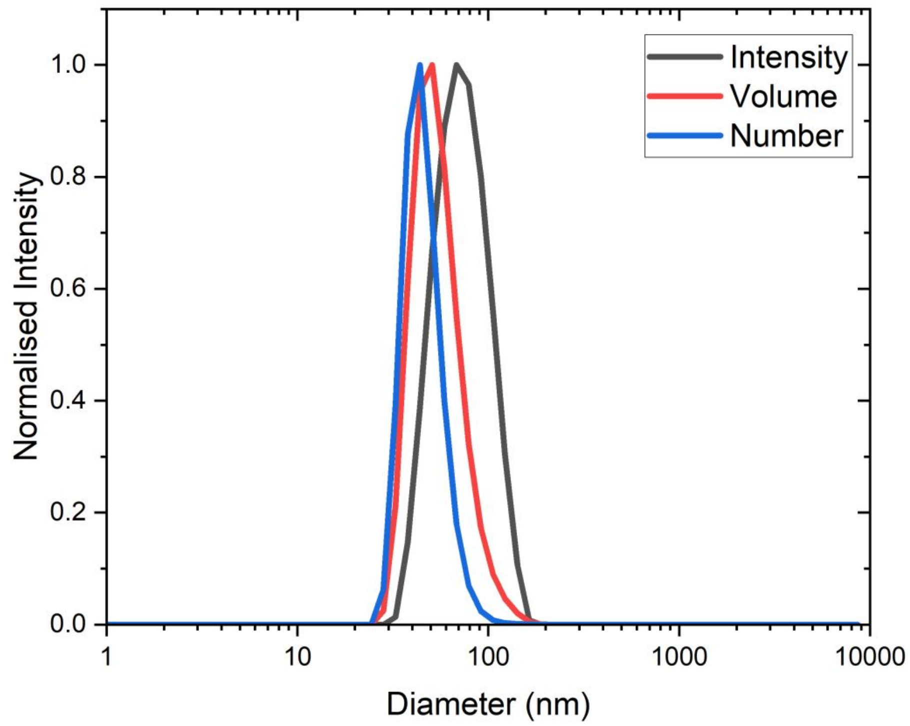

Figure S6 – DLS intensity, volume, and number distribution for unfunctionalized (citrate-stabilised) $AuNP_{16}$

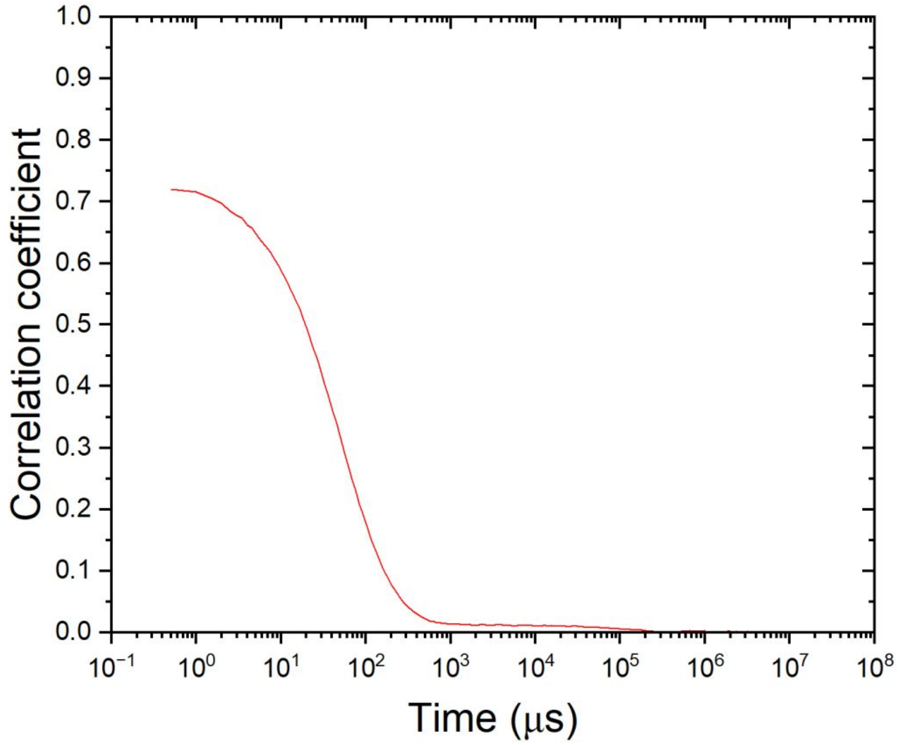

Figure S7 – DLS Correlogram for unfunctionalized (citrate-stabilised) $AuNP_{16}$

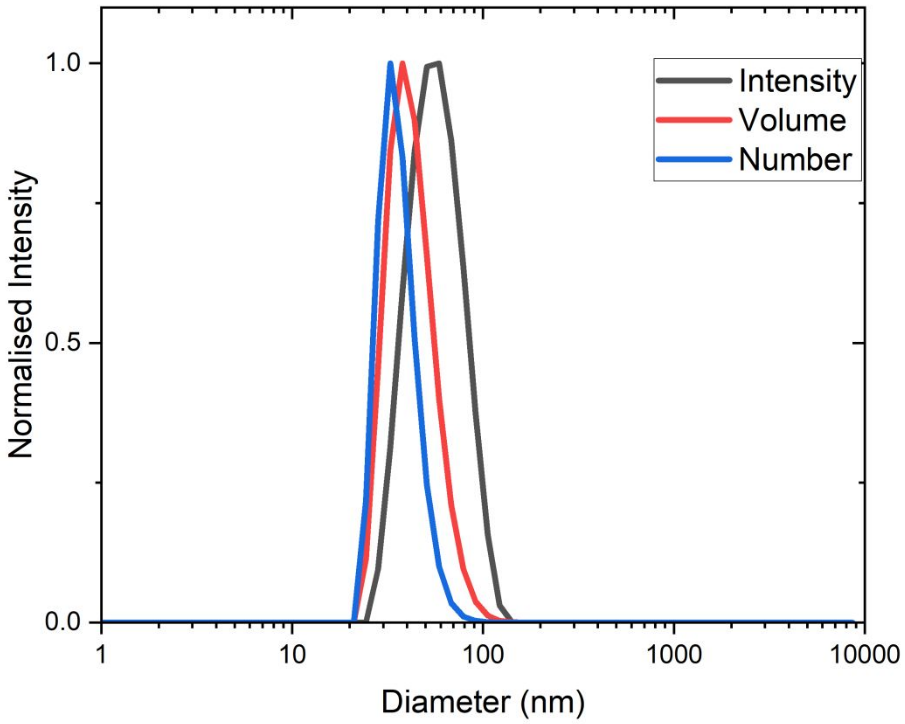

Figure S8 – DLS intensity, volume, and number distribution for unfunctionalized (citrate stabilised) $AuNP_{30}$

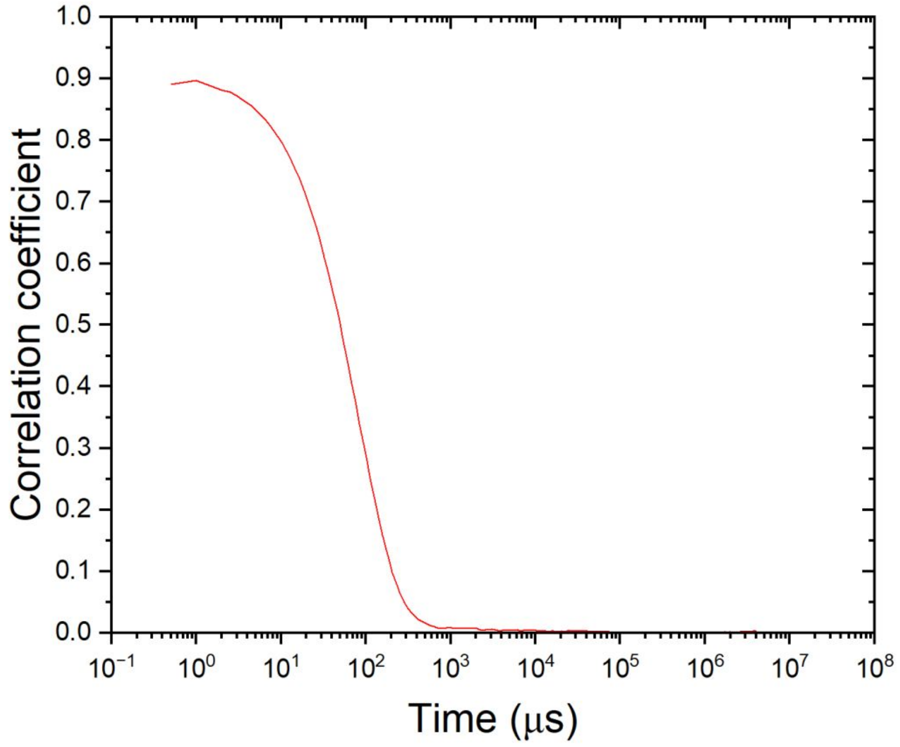

Figure S9 – DLS Correlogram for unfunctionalized (citrate-stabilised) $AuNP_{30}$

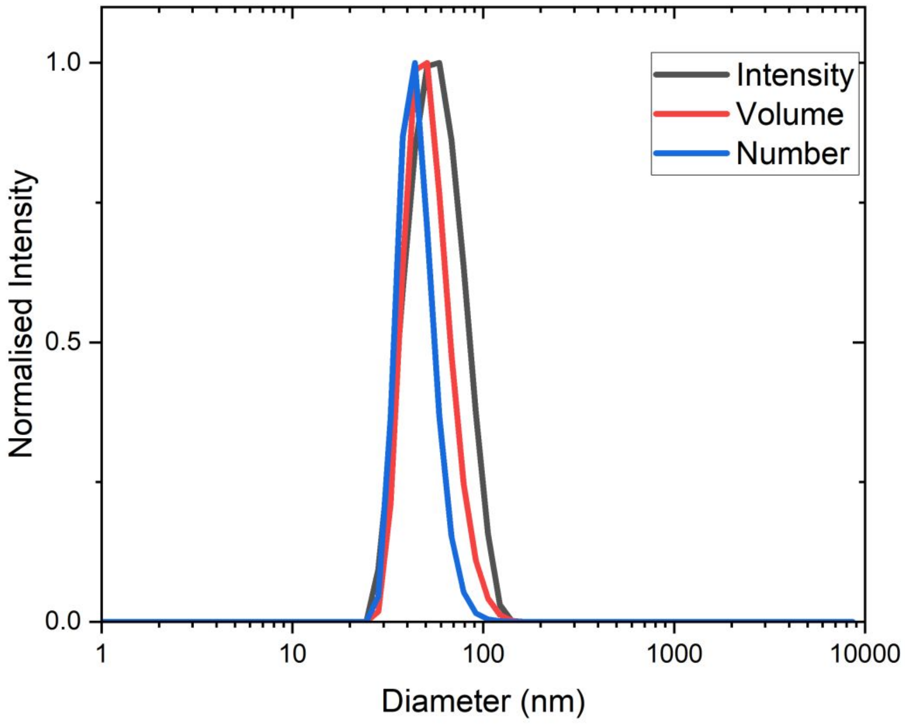

Figure S10 – DLS intensity, volume, and number distribution for unfunctionalized (citrate-stabilised) $AuNP_{40}$

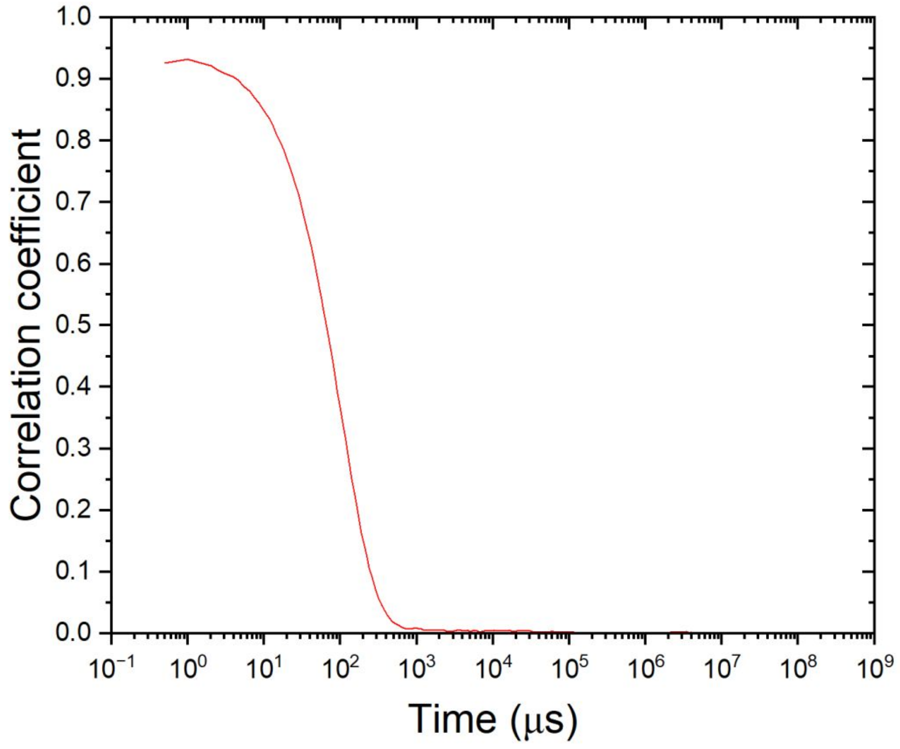

Figure S11 – DLS Correlogram for unfunctionalized (citrate-stabilised) $AuNP_{40}$

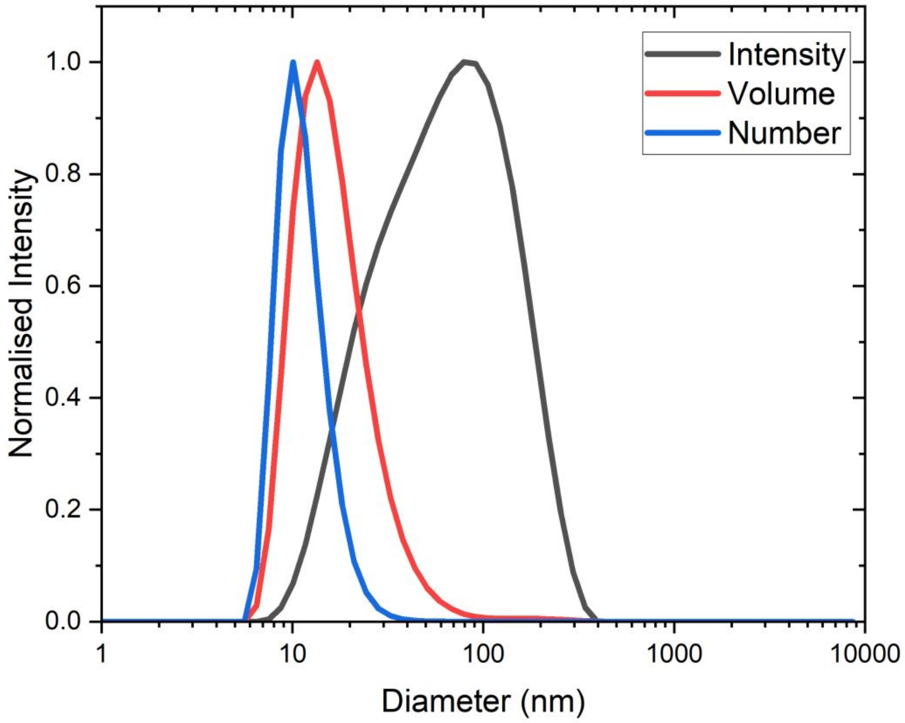

Figure S12 – DLS intensity, volume, and number distribution for GalNAc-1-pHEA$_{42}$@AuNP$_{16}$

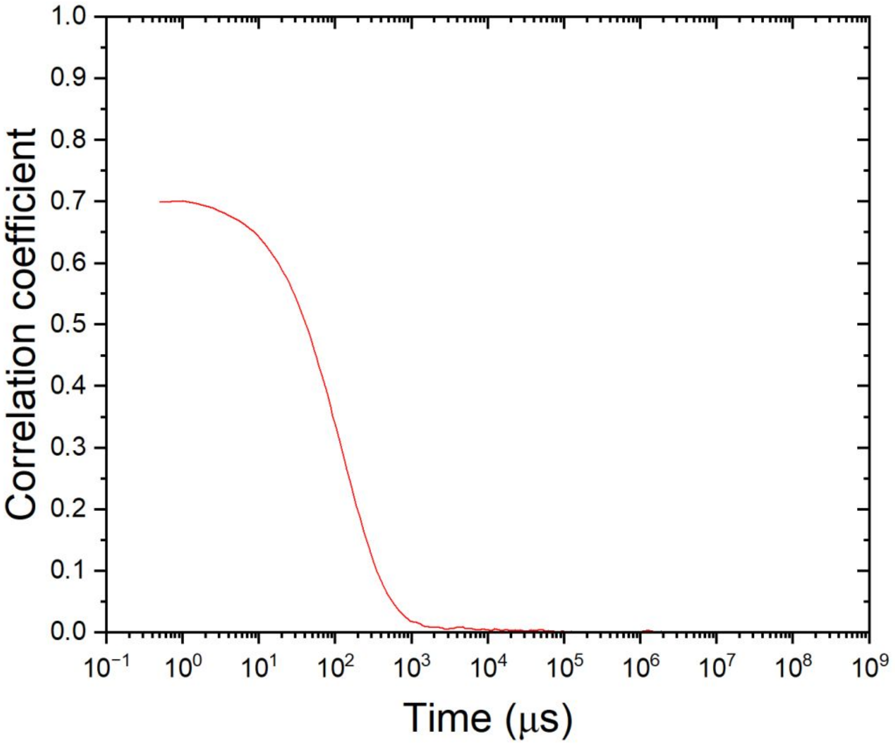

Figure S13 – DLS Correlogram for GalNAc-1-pHEA$_{42}$@AuNP$_{16}$

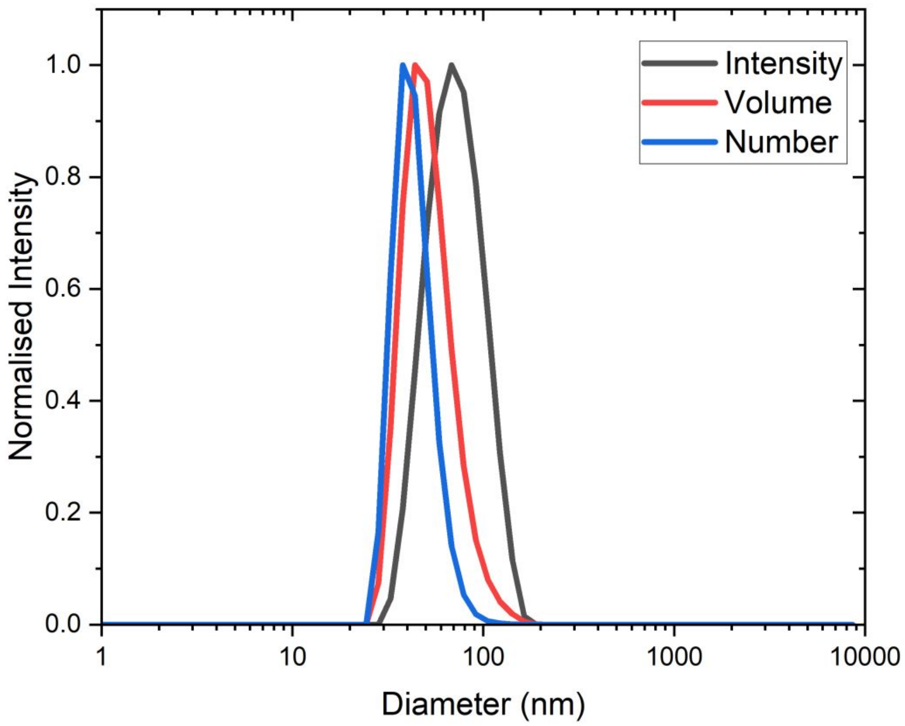

Figure S14 – DLS intensity, volume, and number distribution for GalNAc-1-pHEA$_{42}$@AuNP$_{40}$

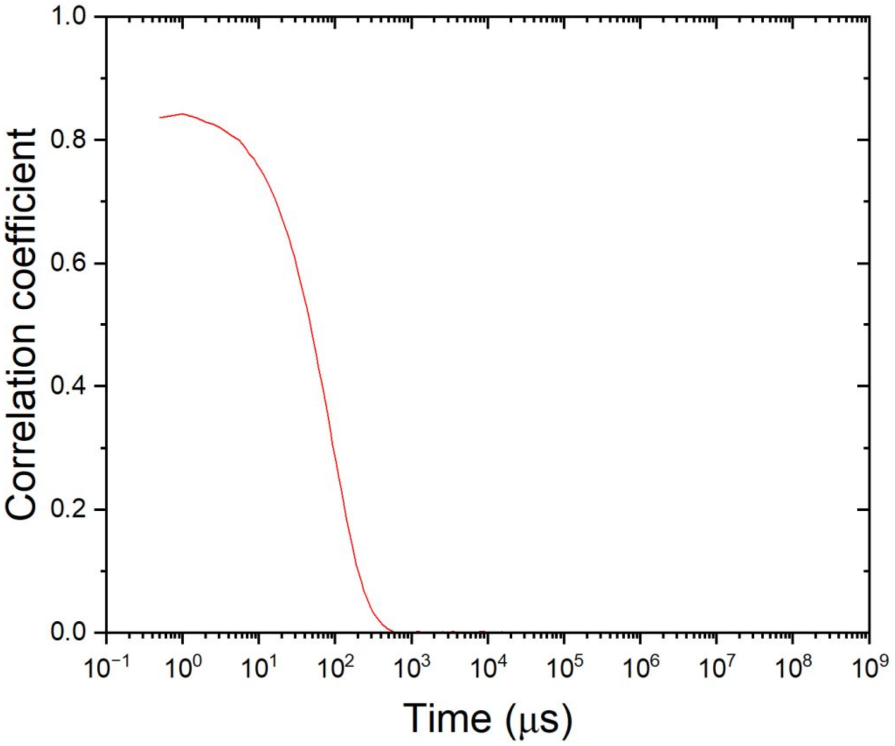

Figure S15 – DLS Correlogram for GalNAc-1-pHEA$_{42}$@AuNP$_{40}$

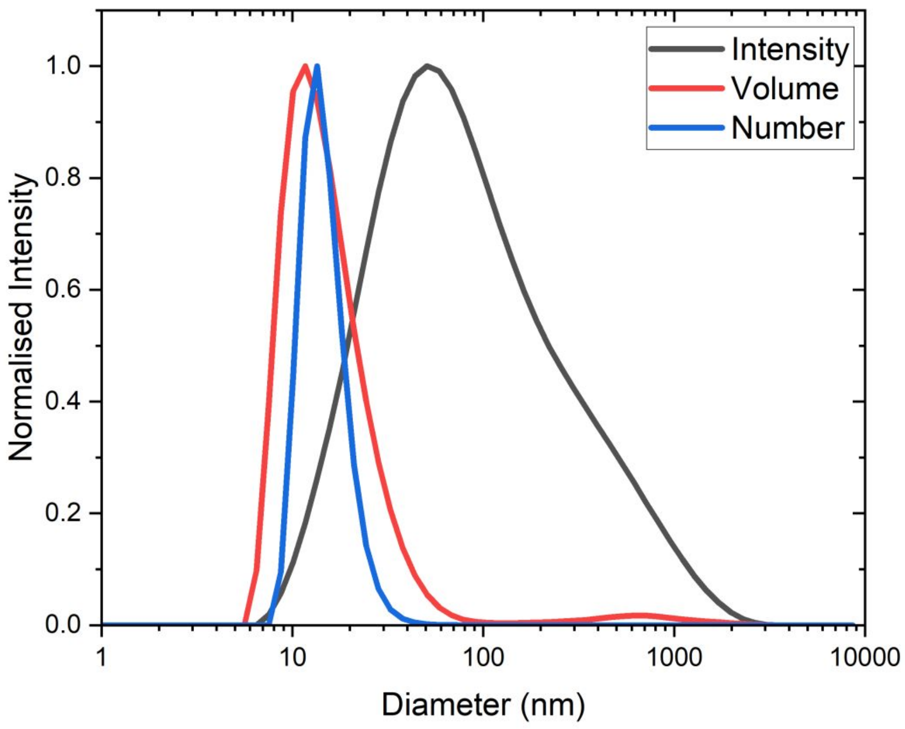

Figure S16 – DLS intensity, volume, and number distribution for Gal-1-$pHEA_{42}$@$AuNP_{16}$

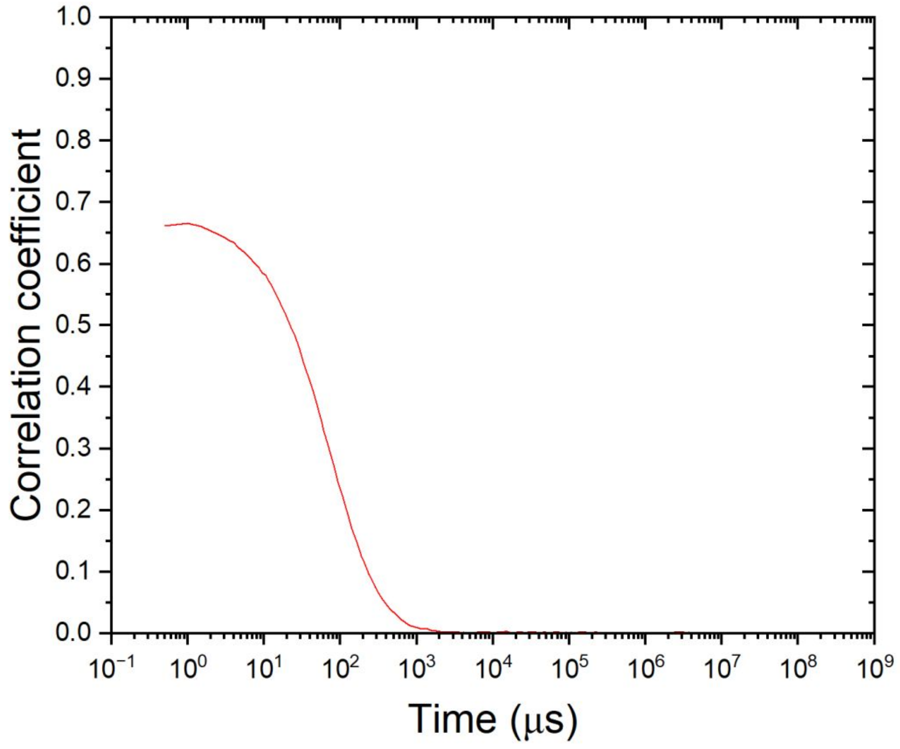

Figure S17 – DLS Correlogram for Gal-1-$pHEA_{42}$@$AuNP_{16}$

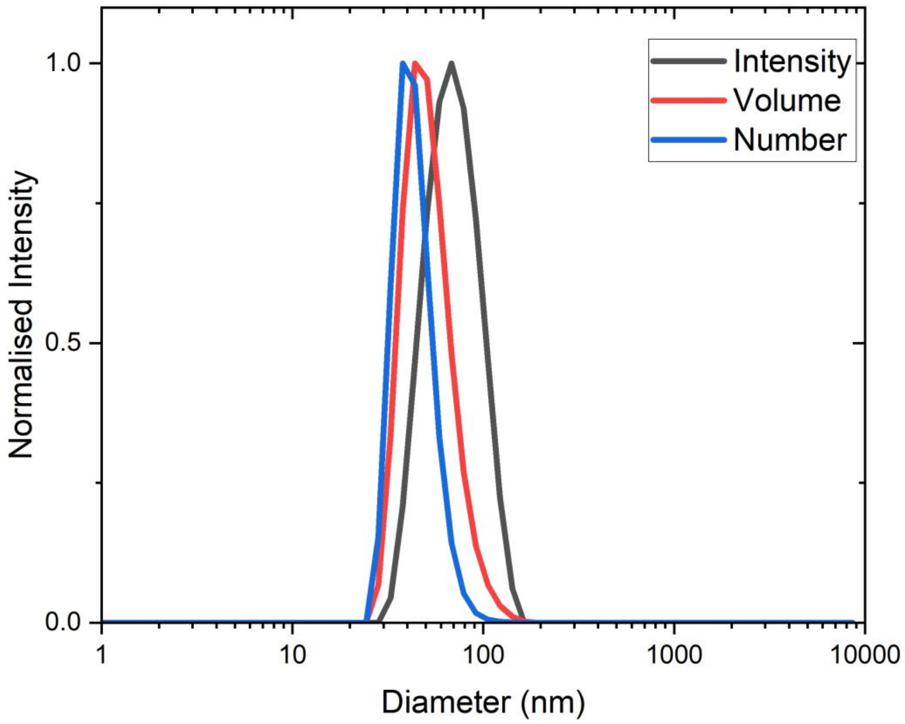

Figure S18 – DLS intensity, volume, and number distribution for Gal-1-pHEA$_{42}$@AuNP$_{40}$

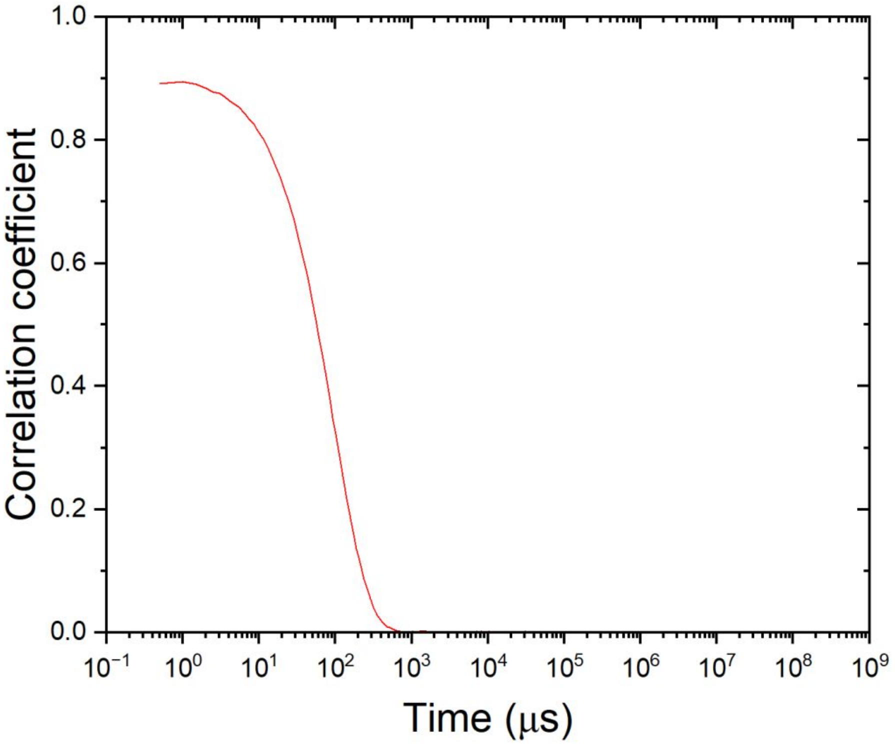

Figure S19 – DLS Correlogram for Gal-1-pHEA$_{42}$@AuNP$_{40}$

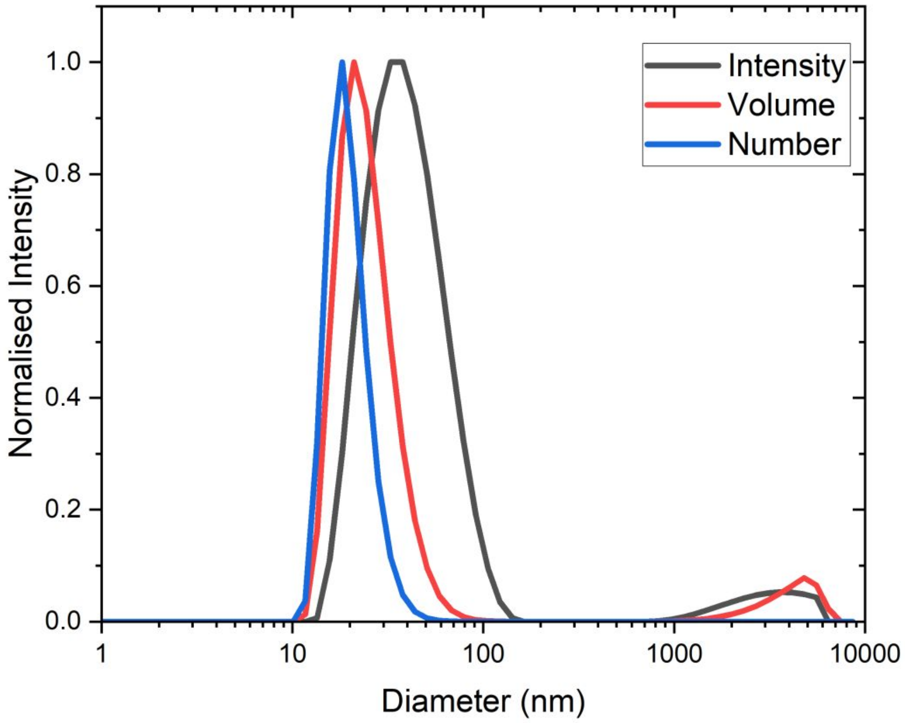

Figure S20 – DLS intensity, volume, and number distribution for Gal-2-pHEA$_{25}$@AuNP$_{16}$

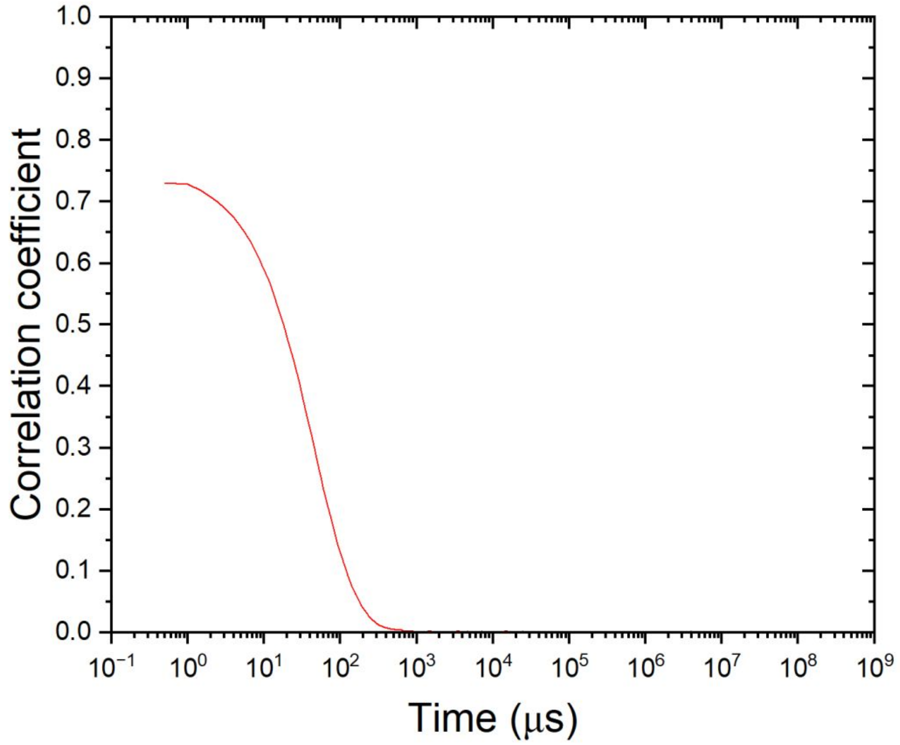

Figure S21 – DLS Correlogram for Gal-2-pHEA$_{25}$@AuNP$_{16}$

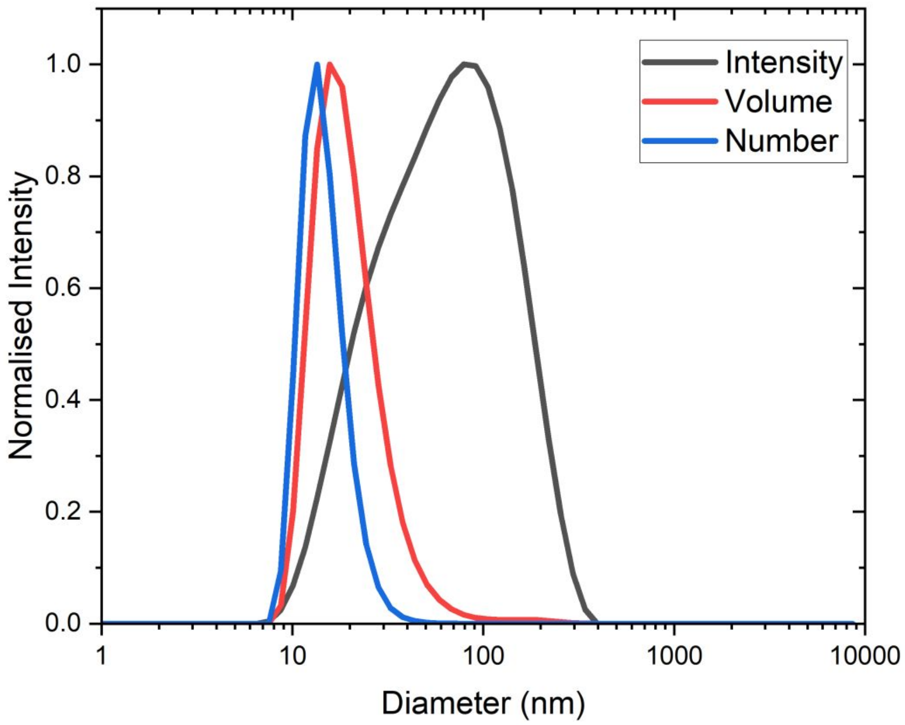

Figure S22 – DLS intensity, volume, and number distribution for Gal-2-$pHEA_{42}$@$AuNP_{16}$

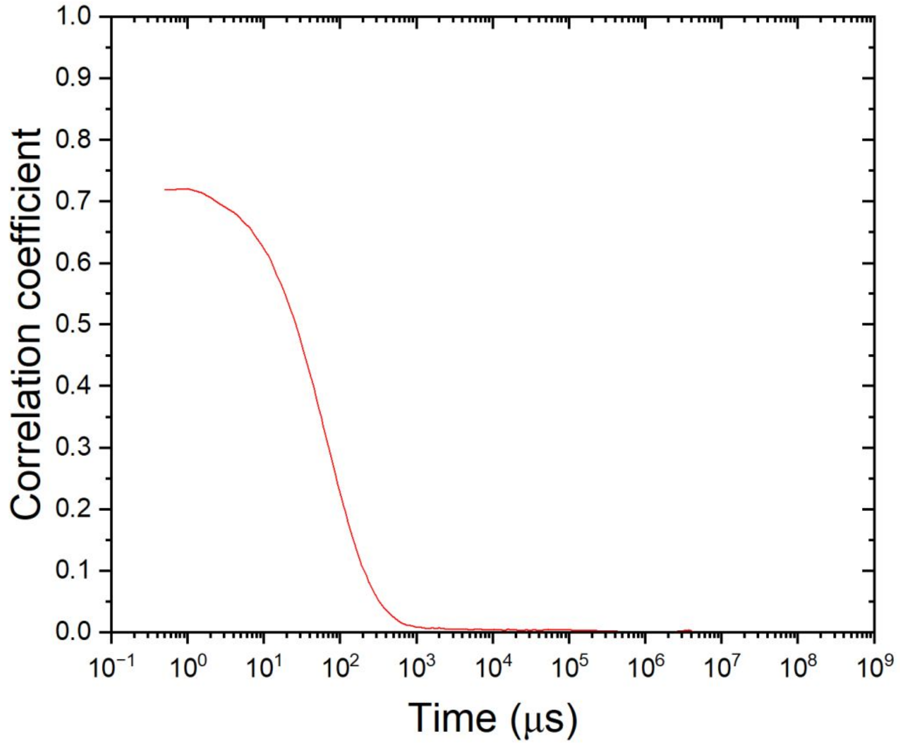

Figure S23 – DLS Correlogram for Gal-2-$pHEA_{42}$@$AuNP_{16}$

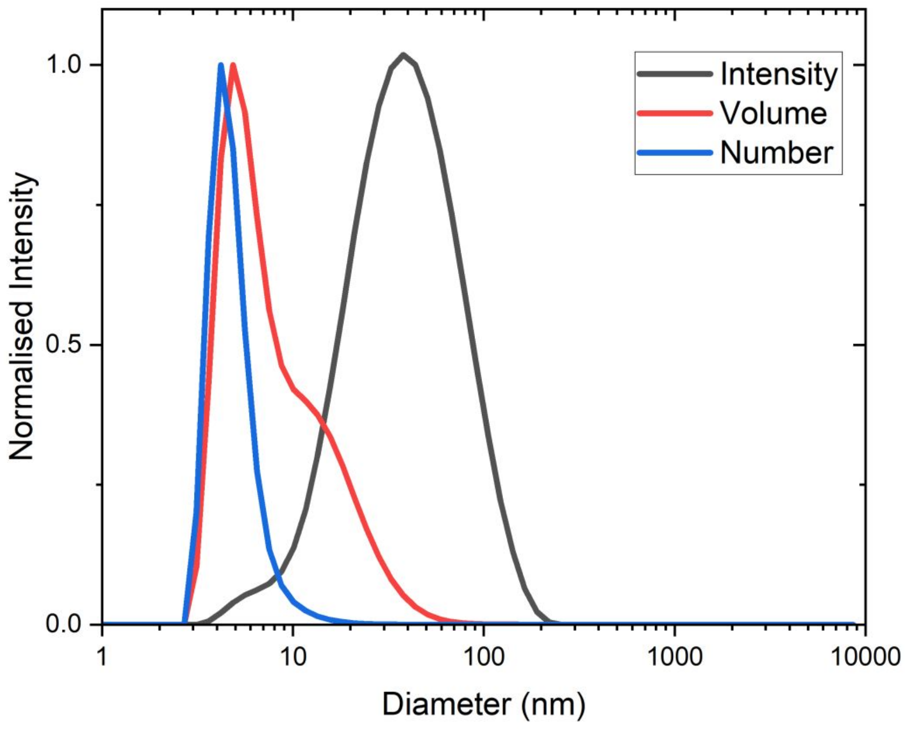

Figure S24 – DLS intensity, volume, and number distribution for Gal-2-pHEA$_{25}$@AuNP$_{30}$

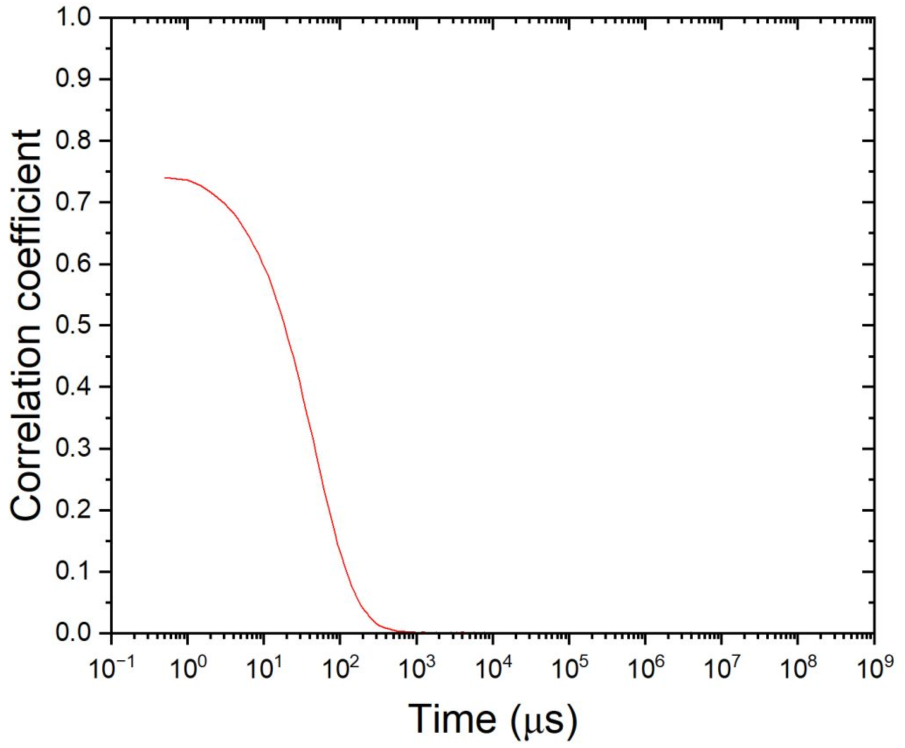

Figure S25 – DLS Correlogram for Gal-2-pHEA$_{25}$@AuNP$_{30}$

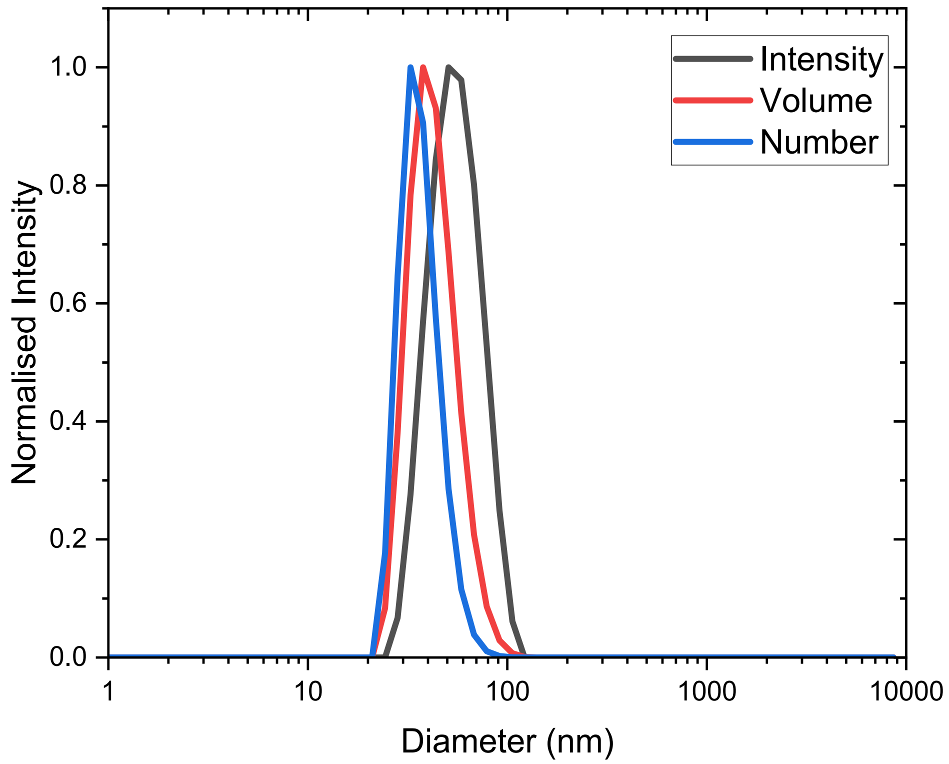

Figure S26 – DLS intensity, volume, and number distribution for Gal-2-pHEA$_{42}$@AuNP$_{30}$

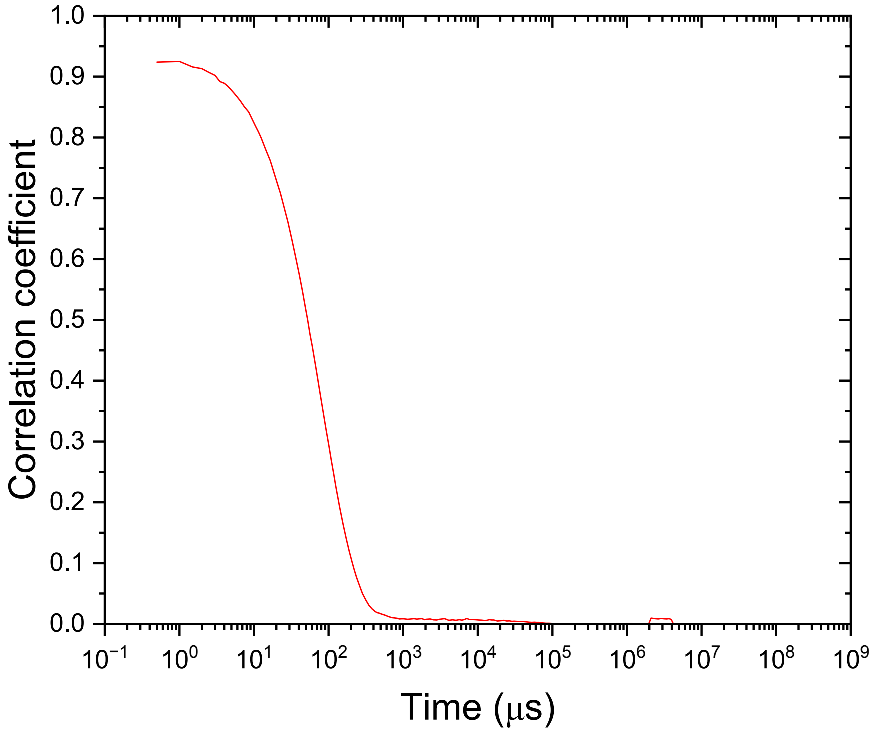

Figure S27 – DLS Correlogram for Gal-2-pHEA$_{42}$@AuNP$_{30}$

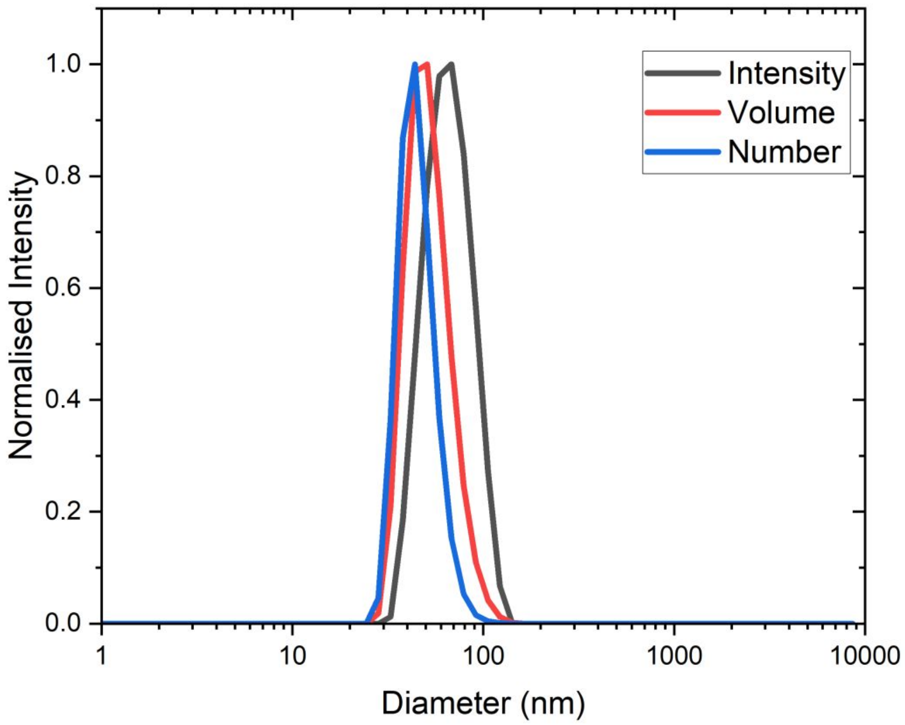

Figure S28 – DLS intensity, volume, and number distribution for Gal-2-pHEA$_{42}$@AuNP$_{40}$

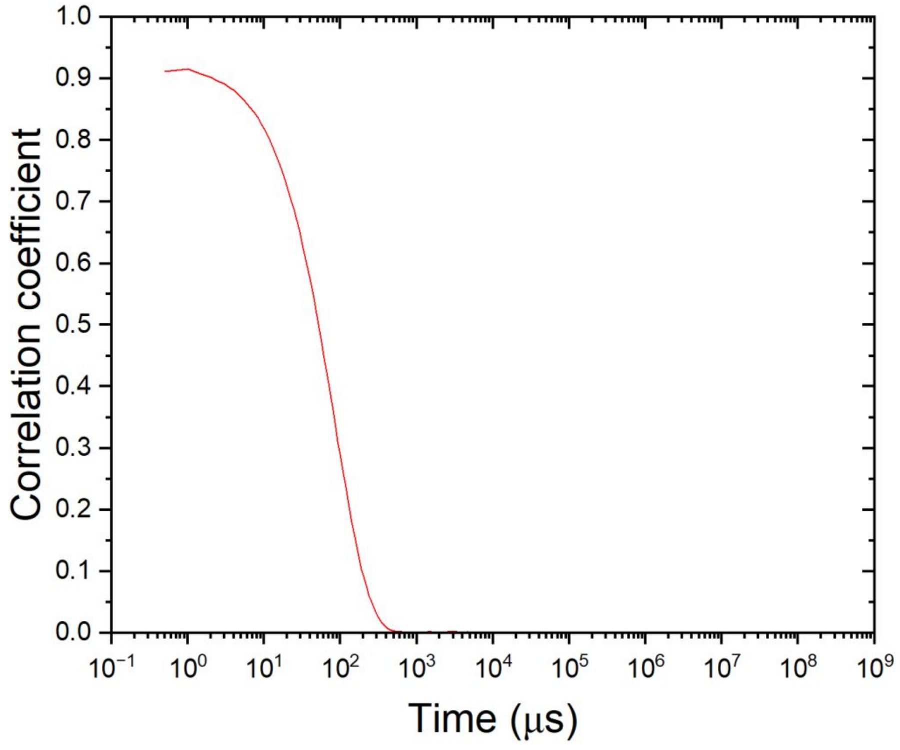

Figure S29 – DLS Correlogram for Gal-2-pHEA$_{42}$@AuNP$_{40}$

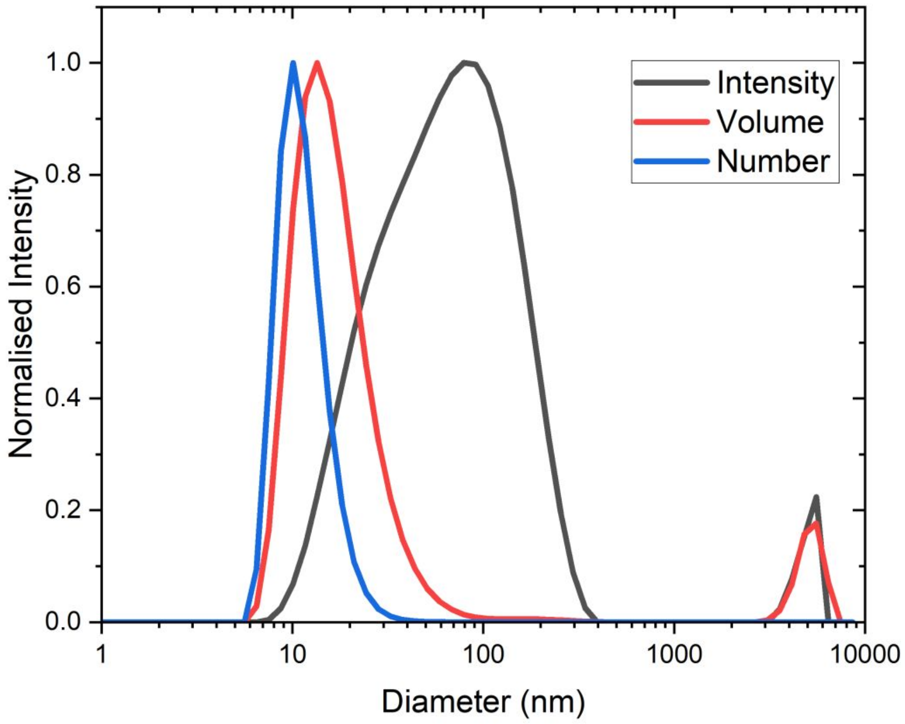

Figure S30 – DLS intensity, volume, and number distribution for Glc-2-pHEA$_{42}$@AuNP$_{16}$

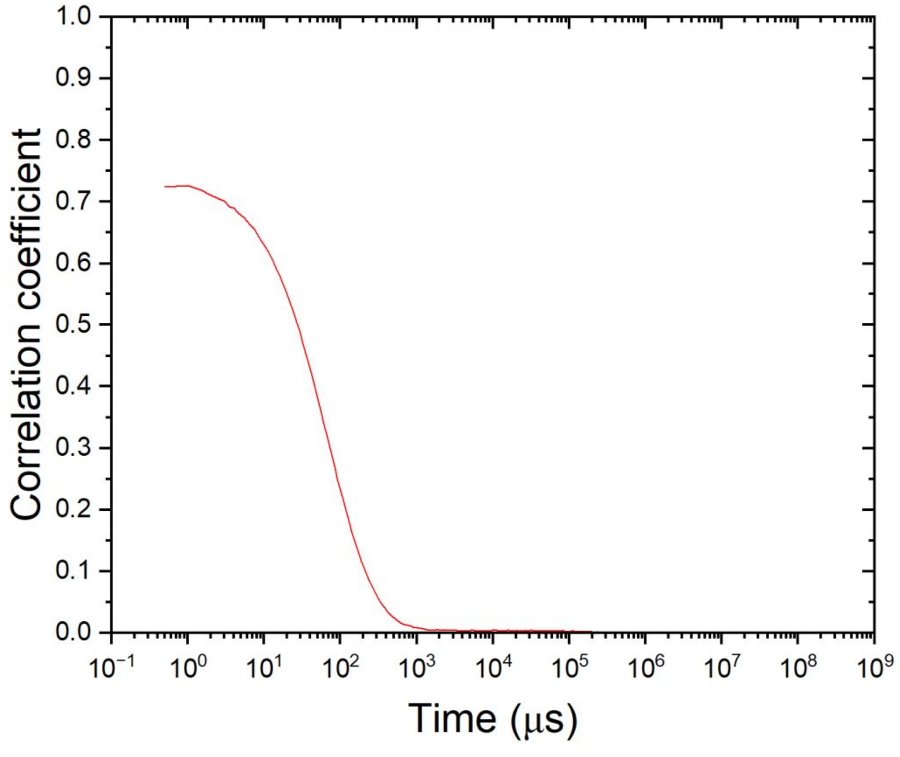

Figure S31 – DLS Correlogram for Glc-2-pHEA$_{42}$@AuNP$_{16}$

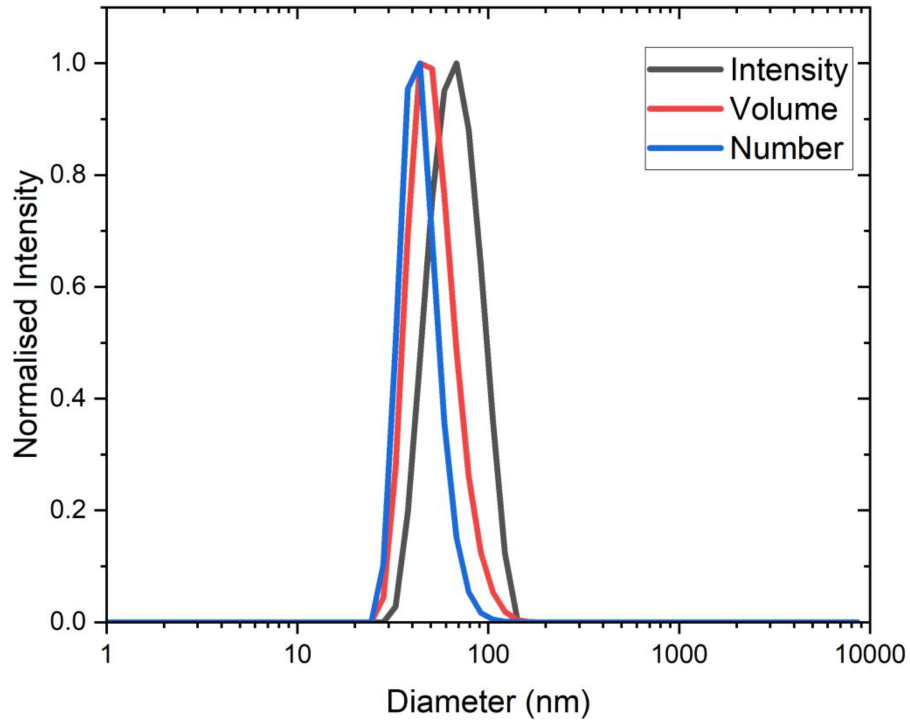

Figure S32 – DLS intensity, volume, and number distribution for Glc-2-pHEA$_{42}$@AuNP$_{40}$

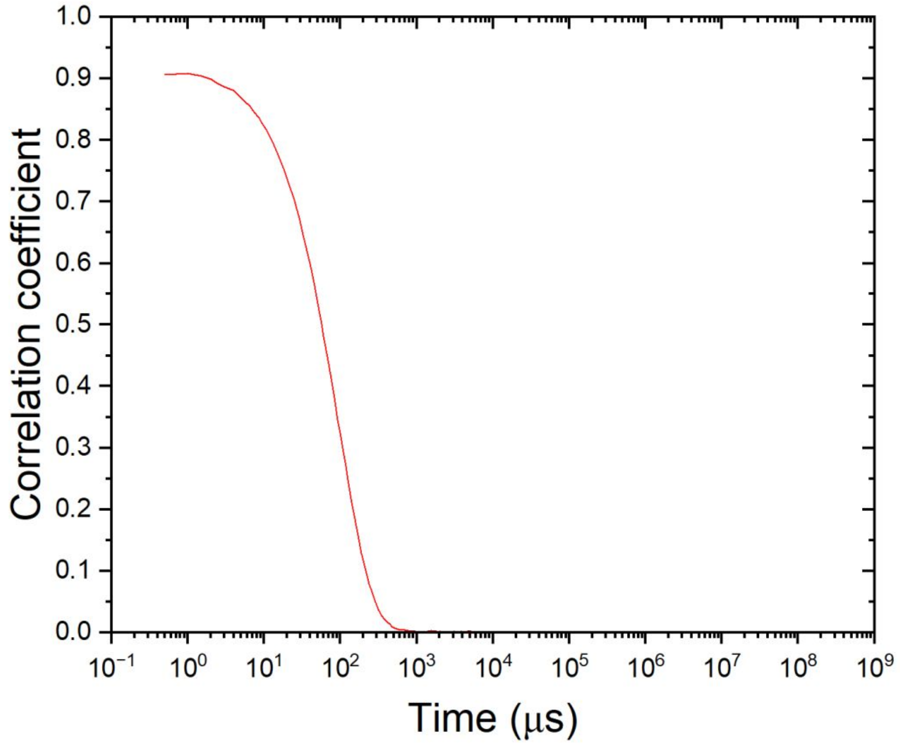

Figure S33 – DLS Correlogram for Glc-2-pHEA$_{42}$@AuNP$_{40}$

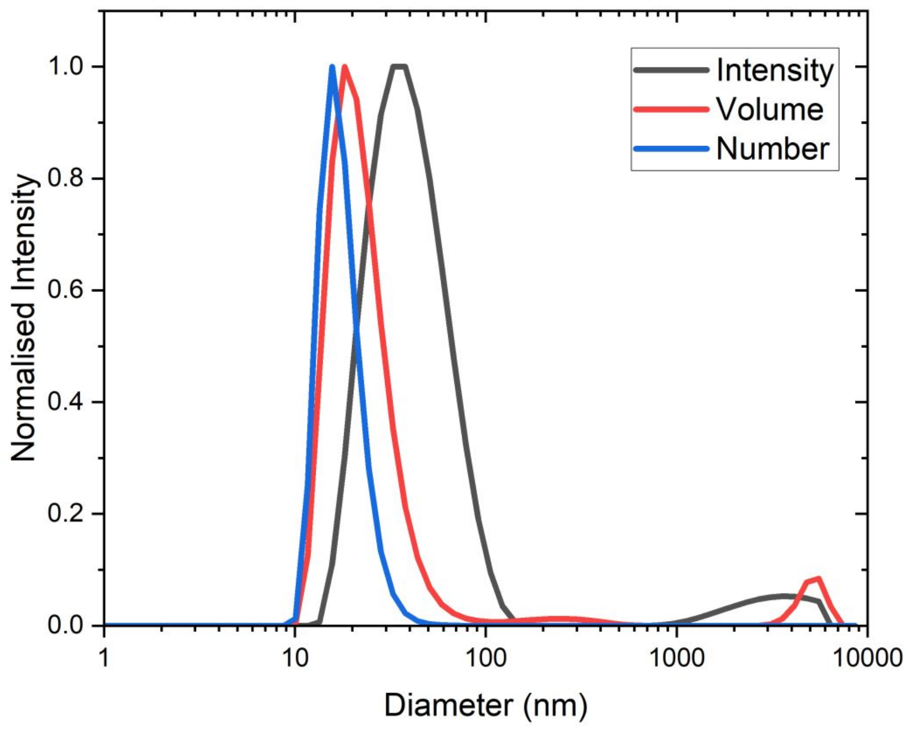

Figure S34 – DLS intensity, volume, and number distribution for Man-2-pHEA$_{42}$@AuNP$_{16}$

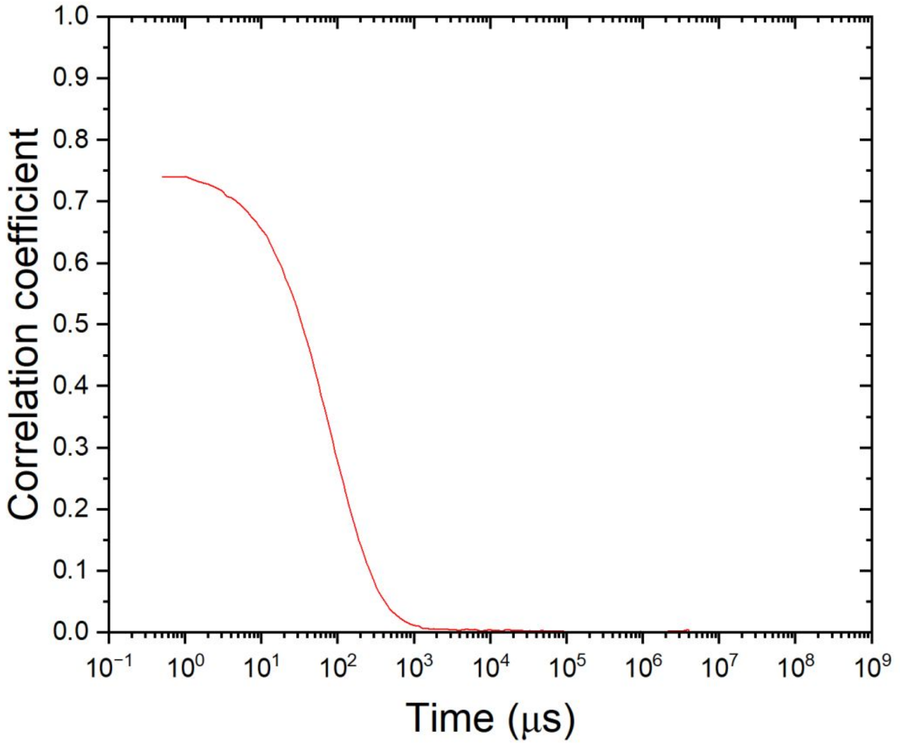

Figure S35 – DLS Correlogram for Man-2-pHEA$_{42}$@AuNP$_{16}$

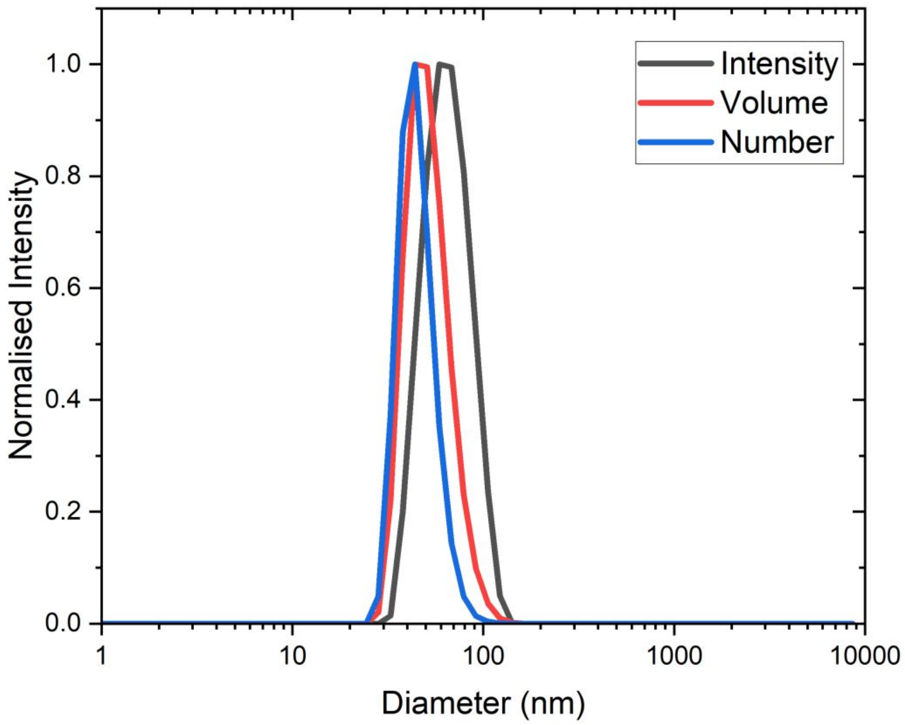

Figure S36 – DLS intensity, volume, and number distribution for Man-2-pHEA$_{42}$@AuNP$_{40}$

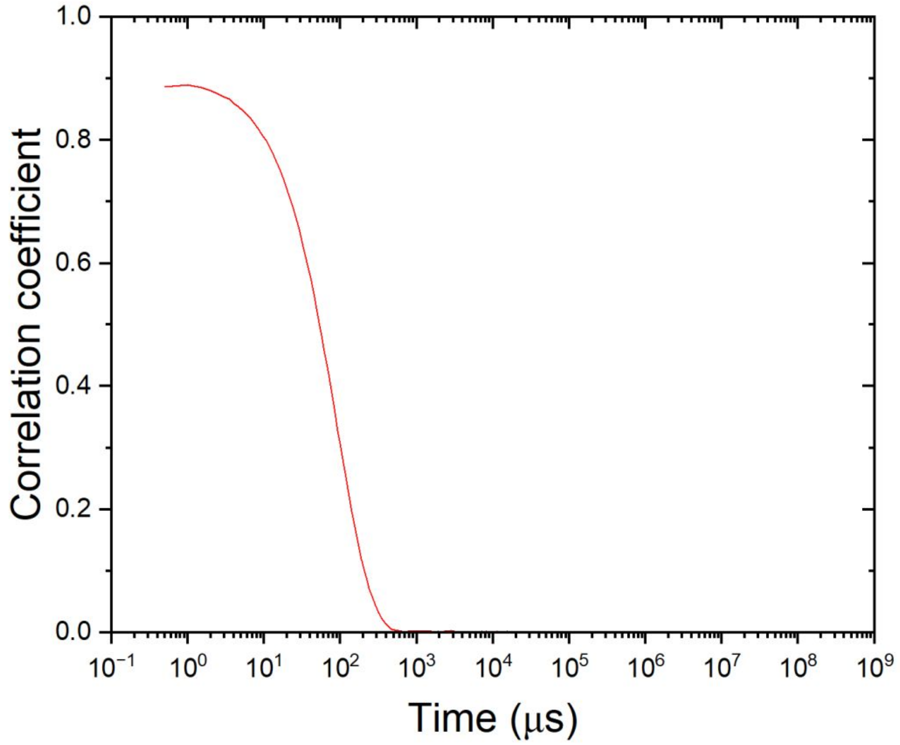

Figure S37 – DLS Correlogram for Man-2-pHEA$_{42}$@AuNP$_{40}$

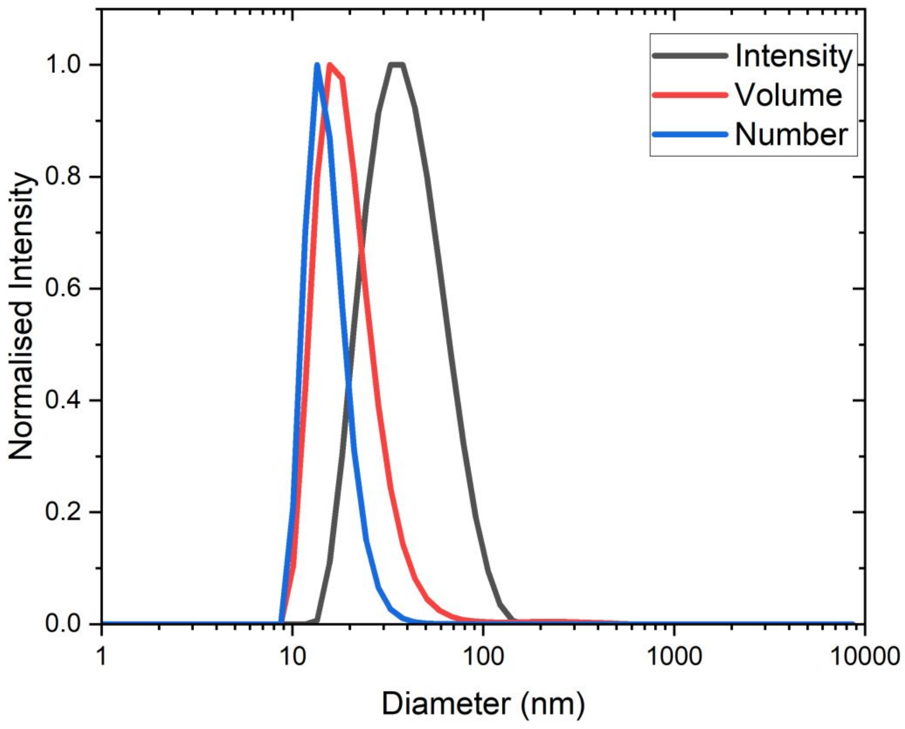

Figure S38 – DLS intensity, volume, and number distribution for Lac-1-pHEA$_{25}$@AuNP$_{16}$

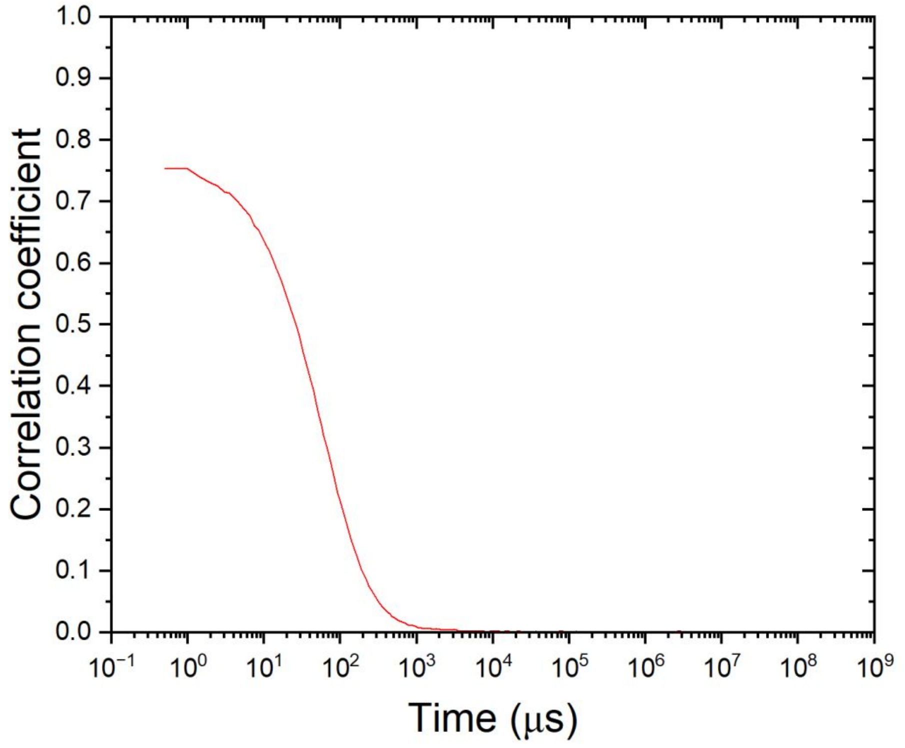

Figure S39 – DLS Correlogram for Lac-1-pHEA$_{25}$@AuNP$_{16}$

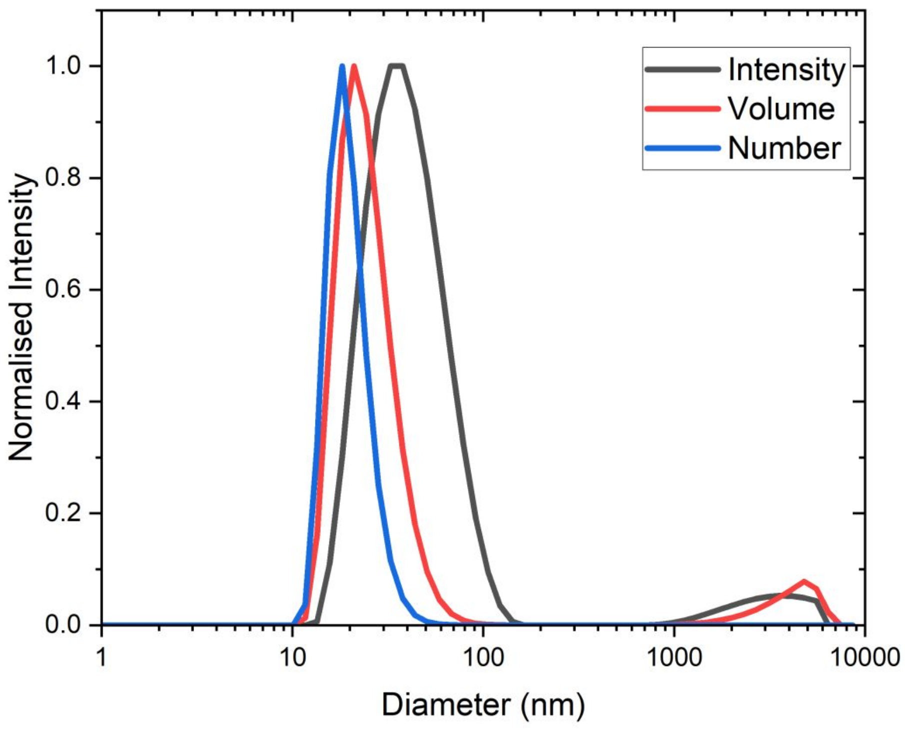

Figure S40 – DLS intensity, volume, and number distribution for Lac-1-pHEA$_{42}$@AuNP$_{16}$

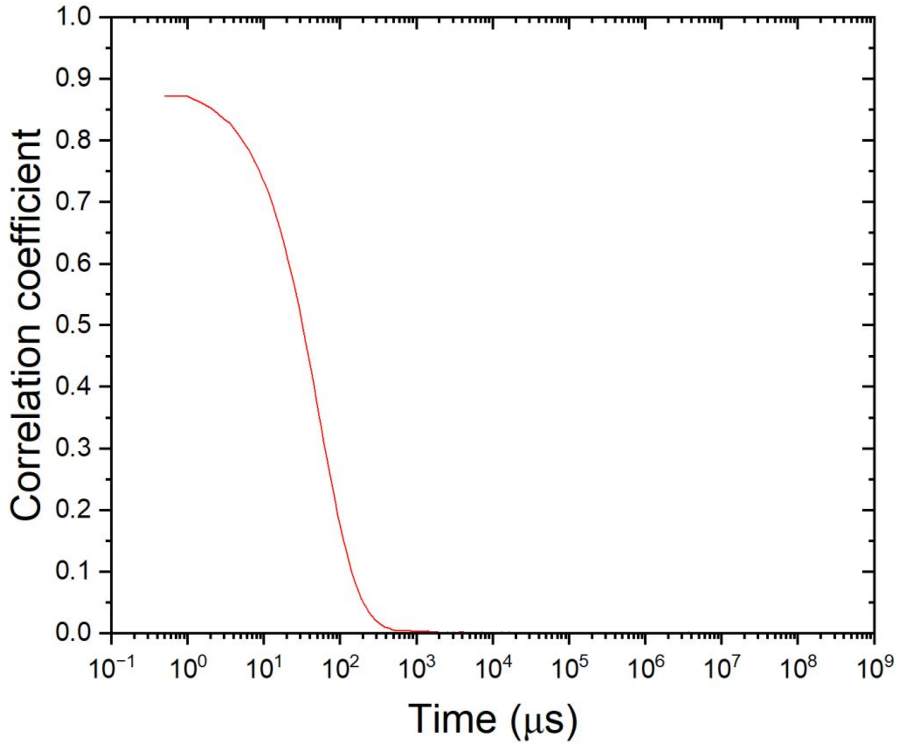

Figure S41 – DLS Correlogram for Lac-1-pHEA$_{42}$@AuNP$_{16}$

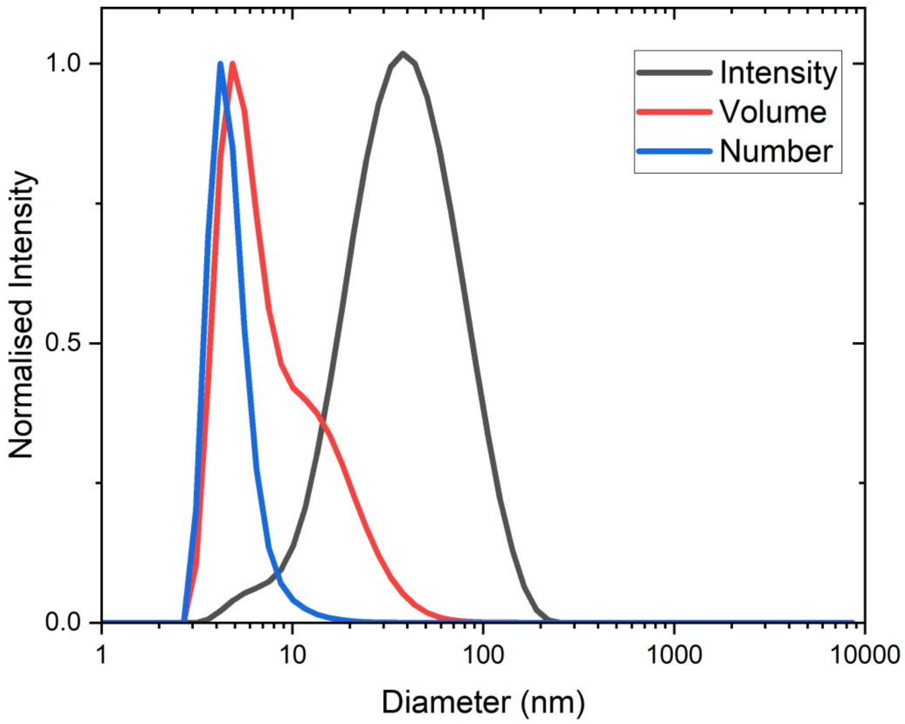

Figure S42 – DLS intensity, volume, and number distribution for Lac-1-pHEA$_{25}$@AuNP$_{30}$

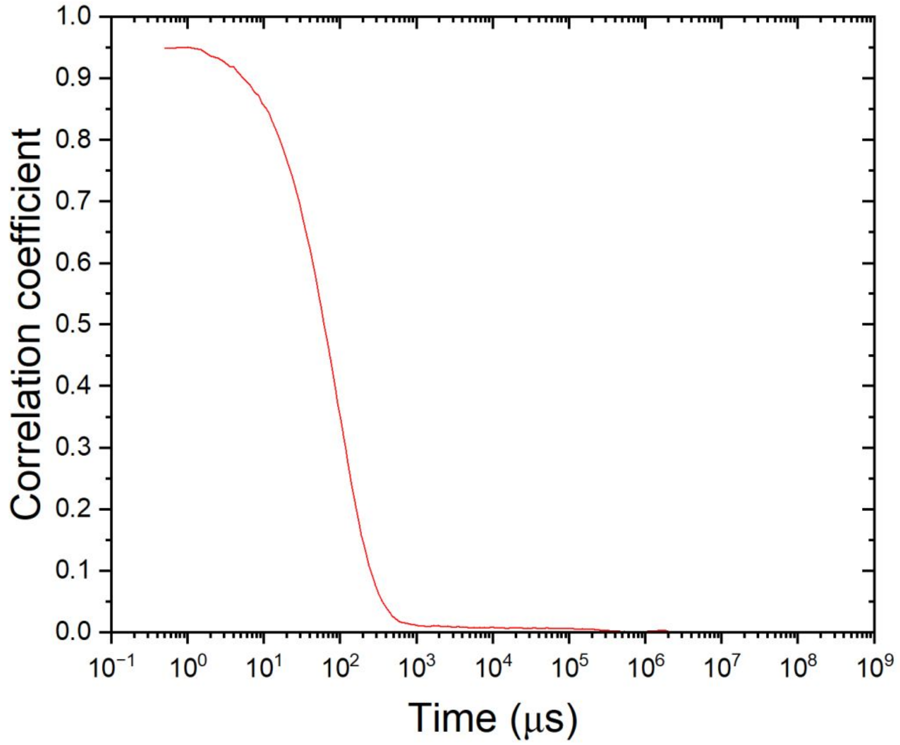

Figure S43 – DLS Correlogram for Lac-1-pHEA$_{25}$@AuNP$_{30}$

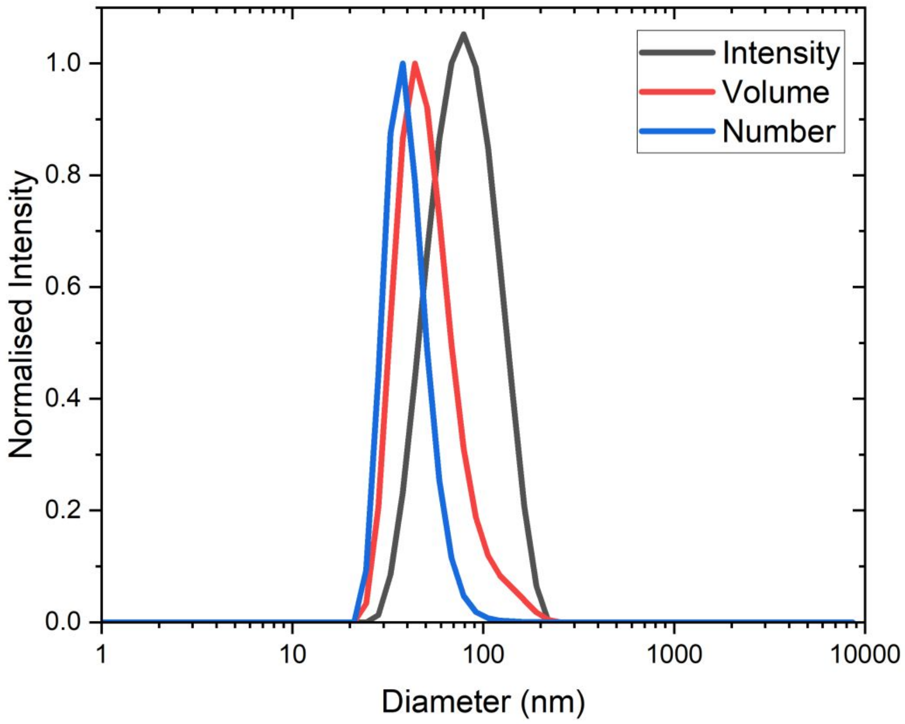

Figure S44 – DLS intensity, volume, and number distribution for Lac-1-pHEA$_{52}$@AuNP$_{40}$

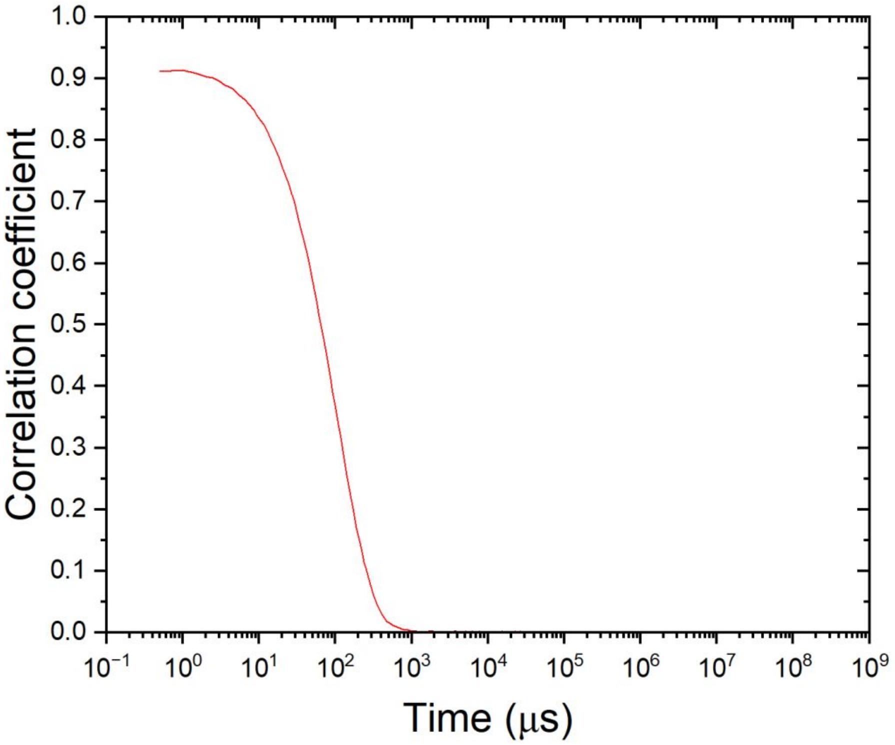

Figure S45 – DLS Correlogram for Lac-1-pHEA$_{52}$@AuNP$_{40}$

UV-vis Aggregation Assay Data

GalNAc-1-pHEA$_{42}$@AuNP$_{16}$

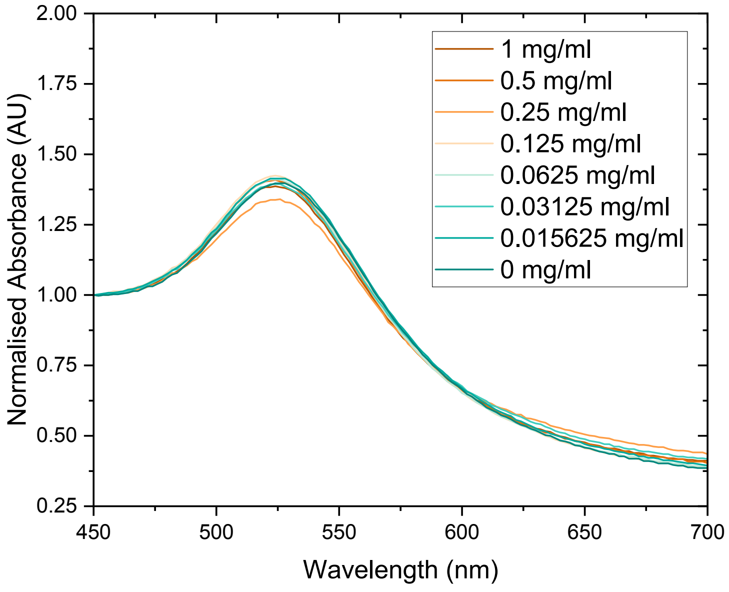

Figure S46 – Plot of normalised absorbance for GalNAc-1-pHEA$_{42}$@AuNP$_{16}$ versus varying concentrations of SBA

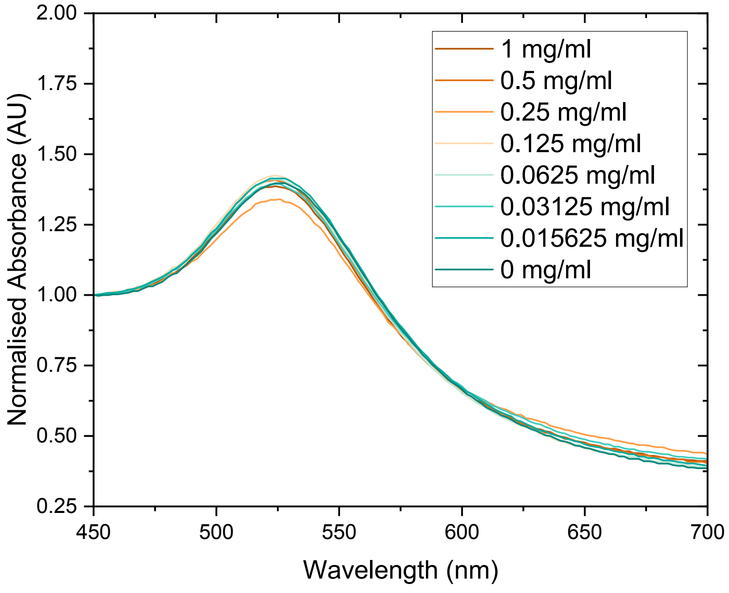

Figure S47 – Plot of normalised absorbance for GalNAc-1-pHEA$_{42}$@AuNP$_{16}$ versus varying concentrations of WGA

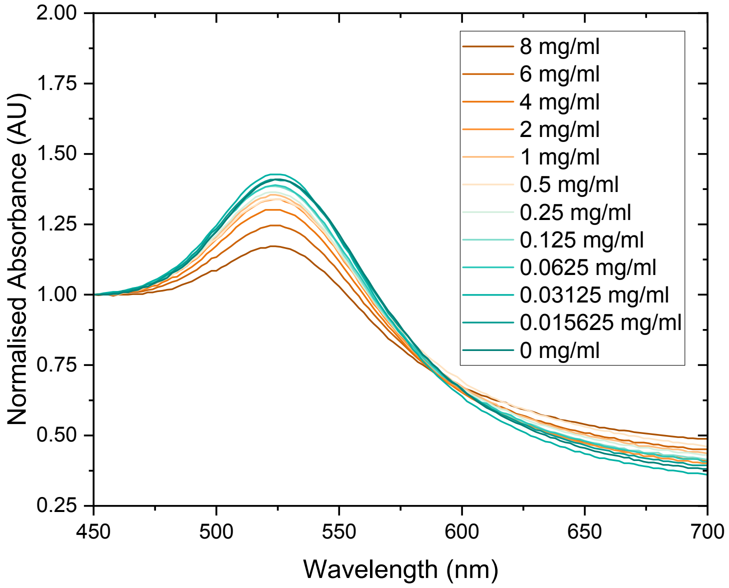

Figure S48 – Plot of normalised absorbance for GalNAc-1-pHEA$_{42}$@AuNP$_{16}$ versus varying concentrations of *C. atrox* venom

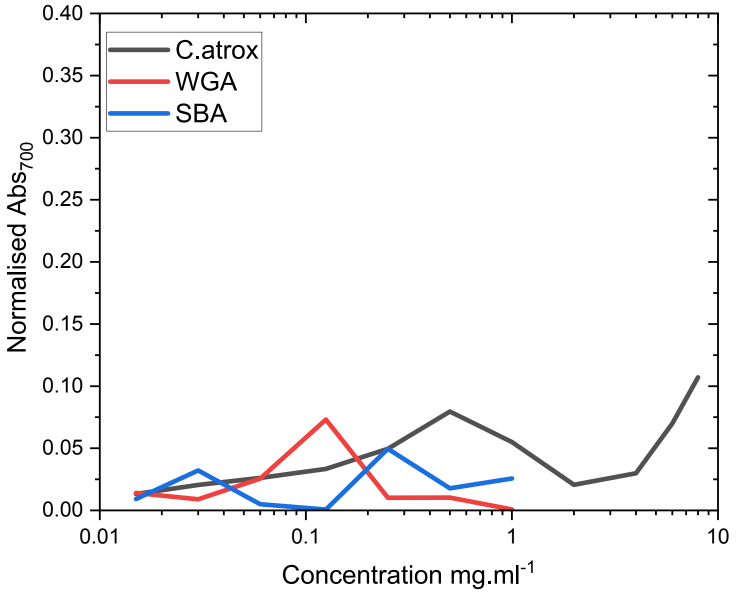

Figure S49 – Plot of normalised absorbance change versus undoped buffer for GalNAc-1-pHEA$_{42}$@AuNP$_{16}$ versus varying concentrations of SBA, WGA and *C. atrox* venom

GalNAc-1-pHEA$_{42}$@AuNP$_{40}$

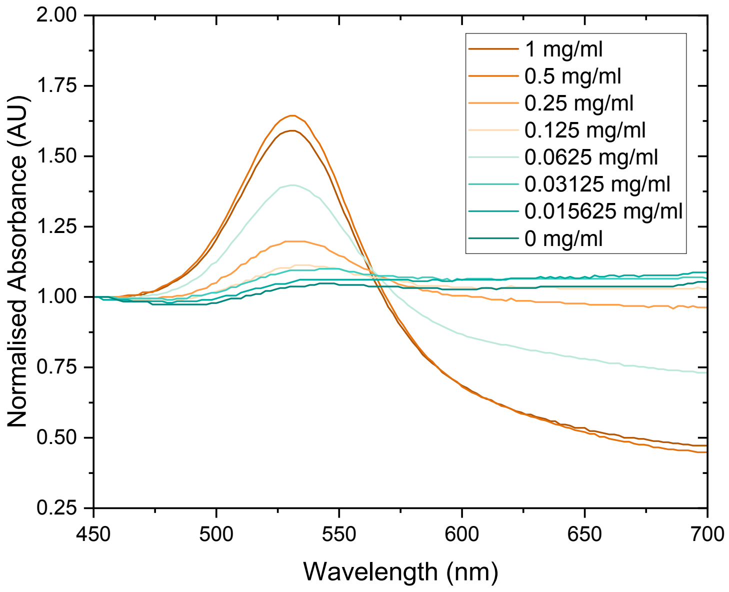

Figure S50 – Plot of normalised absorbance for GalNAc-1-pHEA$_{42}$@AuNP$_{40}$ versus varying concentrations of SBA

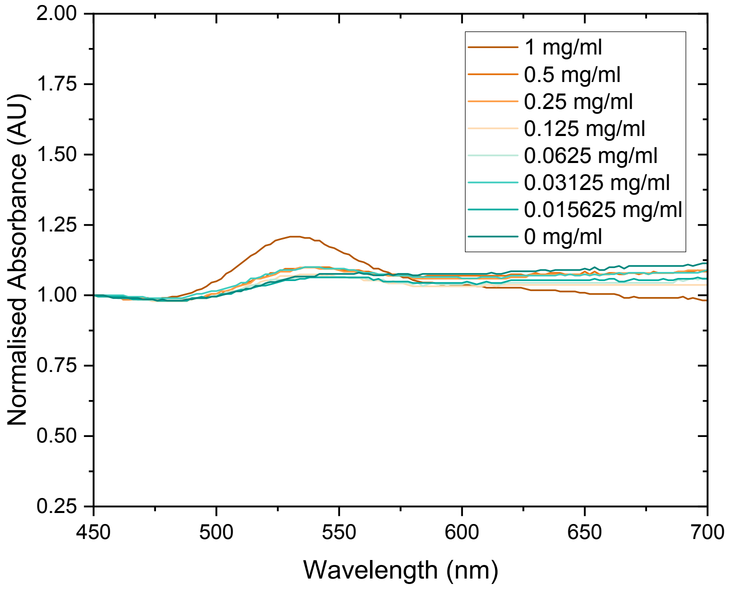

Figure S51 – Plot of normalised absorbance for GalNAc-1-pHEA$_{42}$@AuNP$_{40}$ versus varying concentrations of WGA

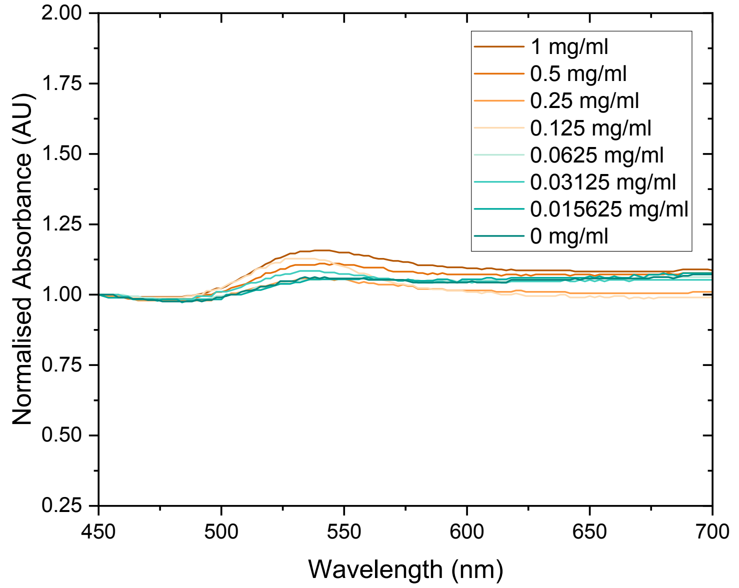

Figure S52 – Plot of normalised absorbance for GalNAc-1-pHEA$_{42}$@AuNP$_{40}$ versus varying concentrations of *C. atrox* venom

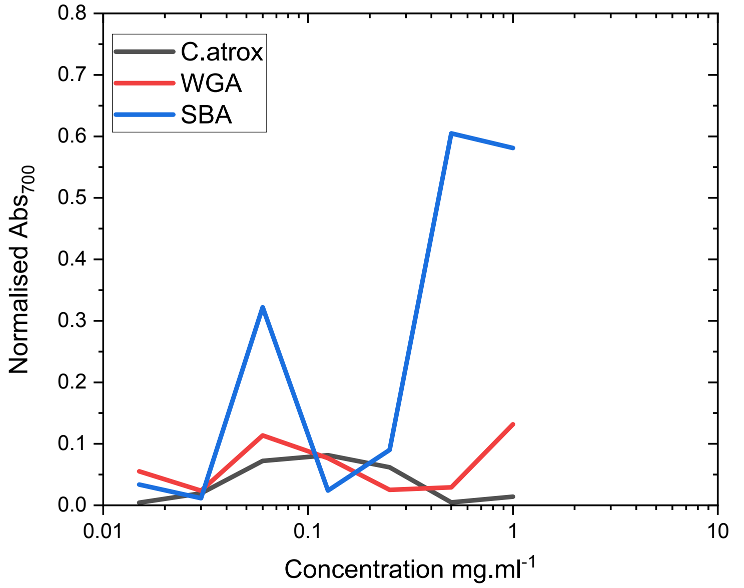

Figure S53 – Plot of normalised absorbance change versus undoped buffer for GalNAc-1-pHEA$_{42}$@AuNP$_{40}$ versus varying concentrations of SBA, WGA and *C. atrox* venom

Gal-1-pHEA$_{42}$@AuNP$_{16}$

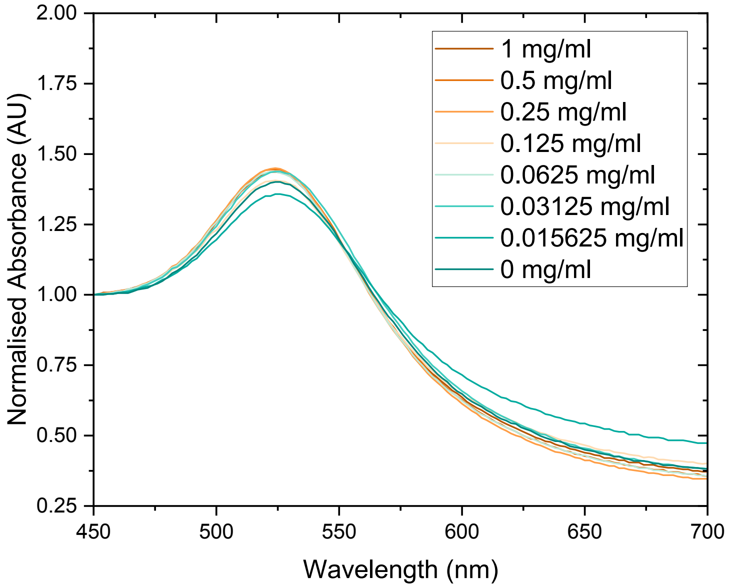

Figure S54 – Plot of normalised absorbance for Gal-1-pHEA$_{42}$@AuNP$_{16}$ versus varying concentrations of SBA

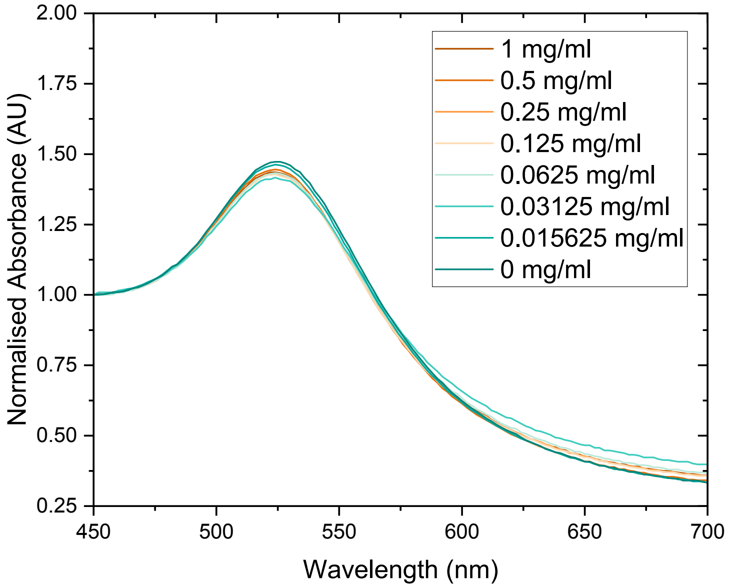

Figure S55 – Plot of normalised absorbance for Gal-1-pHEA$_{42}$@AuNP$_{16}$ versus varying concentrations of WGA

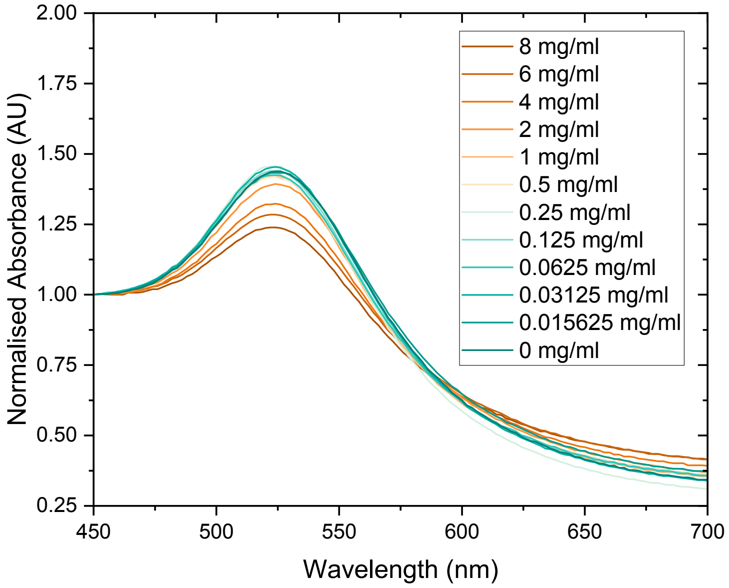

Figure S56 – Plot of normalised absorbance for Gal-1-pHEA$_{42}$@AuNP$_{16}$ versus varying concentrations of *C. atrox* venom

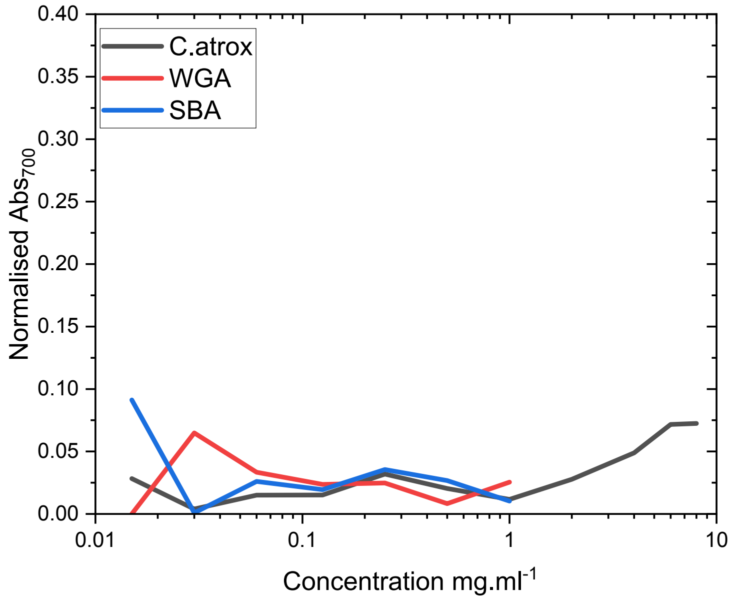

Figure S57 – Plot of normalised absorbance change versus undoped buffer for Gal-1-pHEA$_{42}$@AuNP$_{16}$ versus varying concentrations of SBA, WGA and *C. atrox* venom

Gal-1-pHEA$_{42}$@AuNP$_{40}$

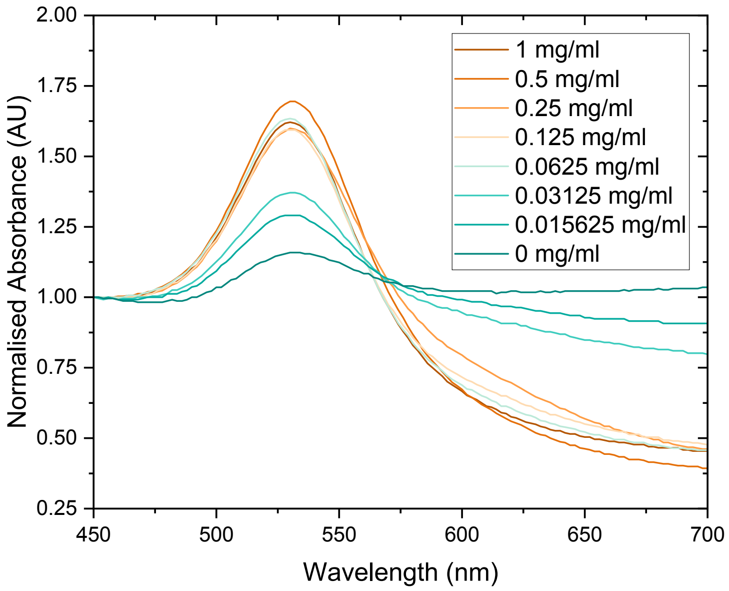

Figure S58 – Plot of normalised absorbance for Gal-1-pHEA$_{42}$@AuNP$_{40}$ versus varying concentrations of SBA

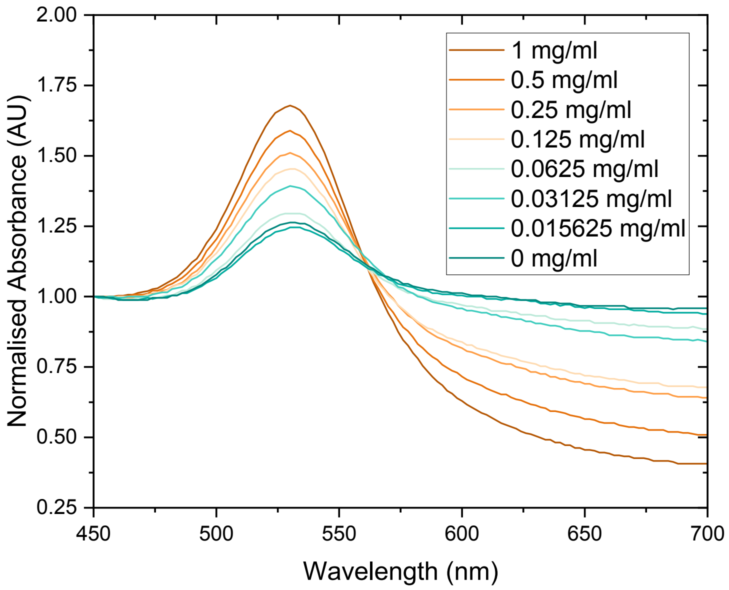

Figure S59 – Plot of normalised absorbance for Gal-1-pHEA$_{42}$@AuNP$_{40}$ versus varying concentrations of WGA

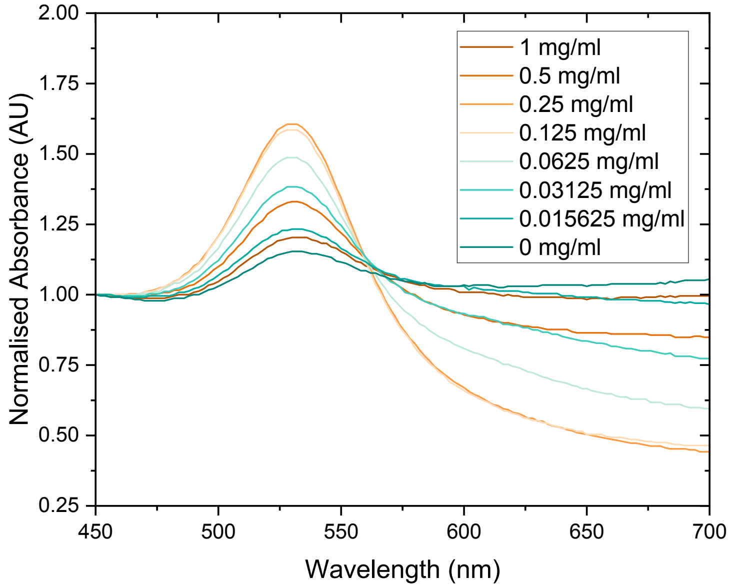

Figure S60 – Plot of normalised absorbance for Gal-1-pHEA$_{42}$@AuNP$_{40}$ versus varying concentrations of *C. atrox* venom

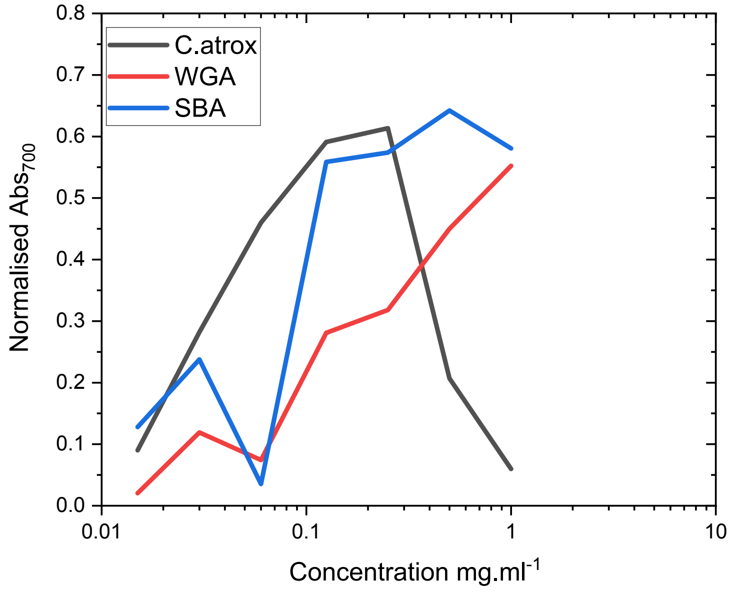

Figure S61 – Plot of normalised absorbance change versus undoped buffer for Gal-1-pHEA$_{42}$@AuNP$_{40}$ versus varying concentrations of SBA, WGA and *C. atrox* venom

Gal-2-pHEA$_{25}$@AuNP$_{16}$

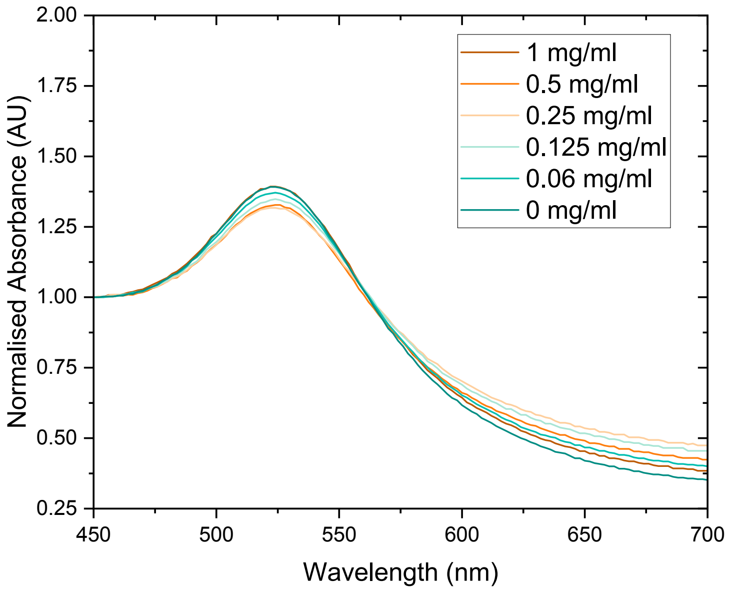

Figure S62 – Plot of normalised absorbance for Gal-2-pHEA$_{25}$@AuNP$_{16}$ versus varying concentrations of SBA

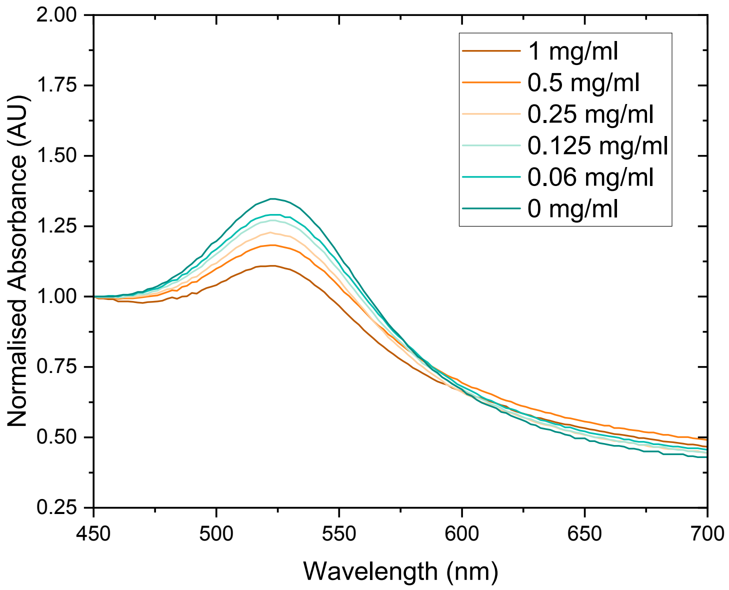

Figure S63 – Plot of normalised absorbance for Gal-2-pHEA$_{25}$@AuNP$_{16}$ versus varying concentrations of WGA

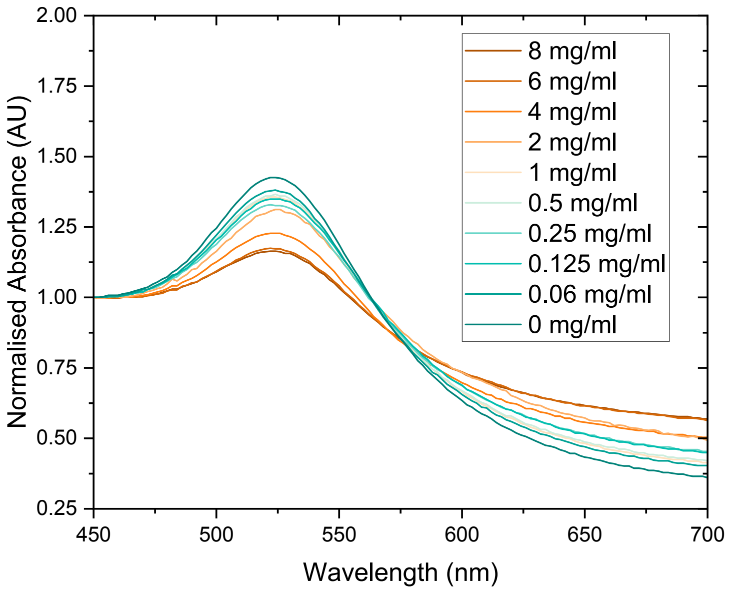

Figure S64 – Plot of normalised absorbance for Gal-2-pHEA$_{25}$@AuNP$_{16}$ versus varying concentrations of *C. atrox* venom

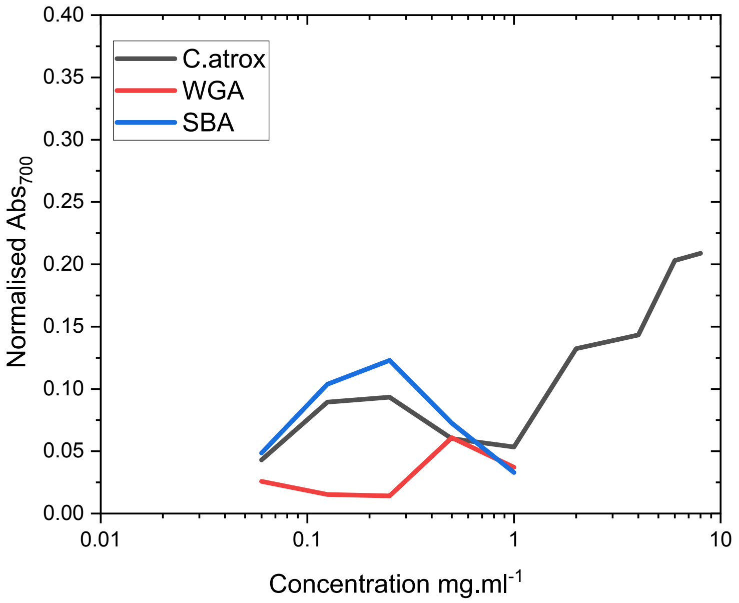

Figure S65 – Plot of normalised absorbance change versus undoped buffer for Gal-2-pHEA$_{25}$@AuNP$_{16}$ versus varying concentrations of SBA, WGA and *C. atrox* venom

Gal-2-pHEA$_{42}$@AuNP$_{16}$

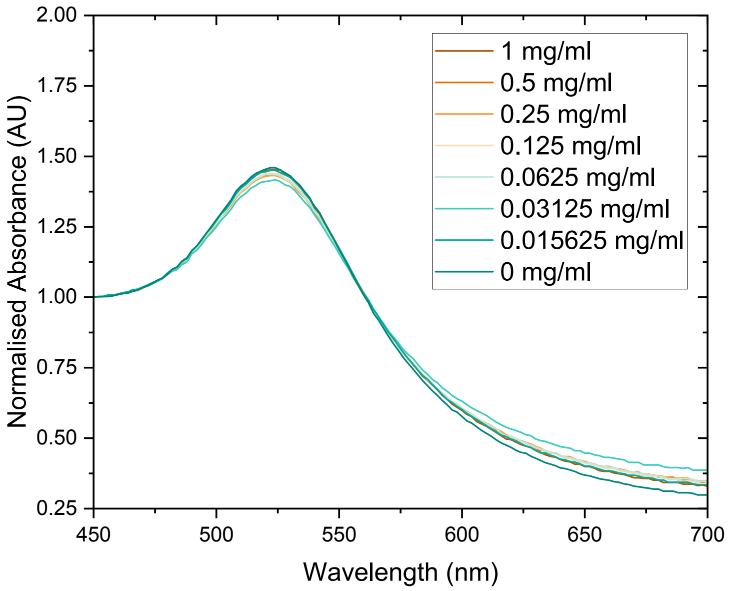

Figure S66 – Plot of normalised absorbance for Gal-2-pHEA$_{42}$@AuNP$_{16}$ versus varying concentrations of SBA

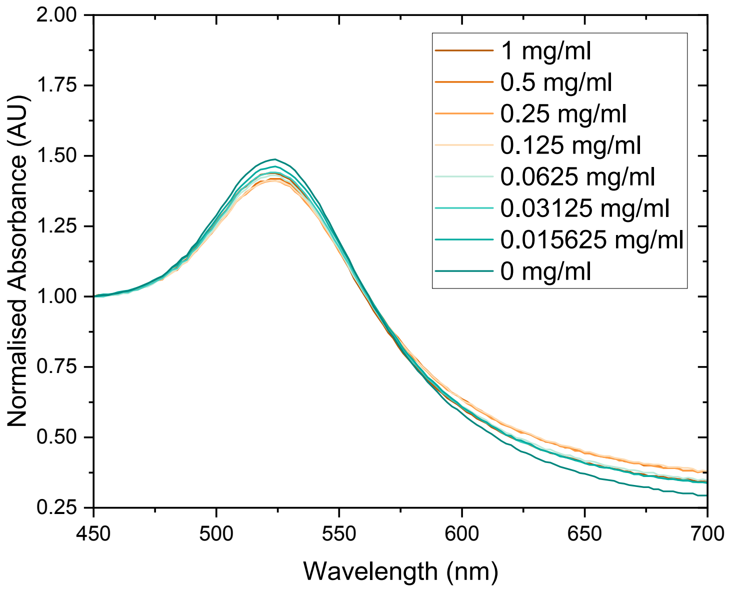

Figure S67 – Plot of normalised absorbance for Gal-2-pHEA$_{42}$@AuNP$_{16}$ versus varying concentrations of WGA

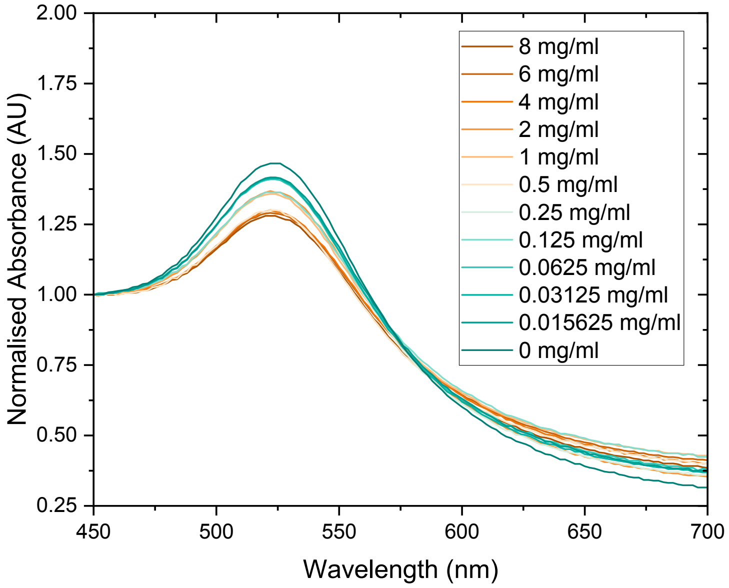

Figure S68 – Plot of normalised absorbance for Gal-2-pHEA$_{42}$@AuNP$_{16}$ versus varying concentrations of *C. atrox* venom

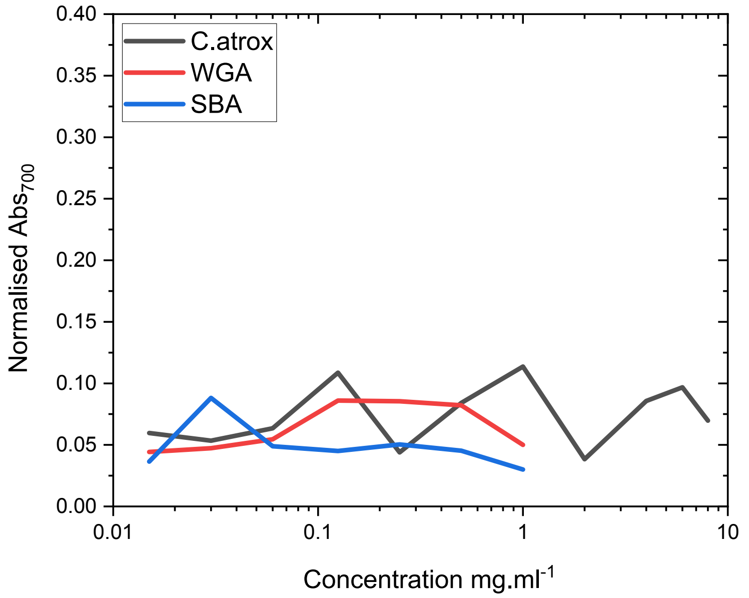

Figure S69 – Plot of normalised absorbance change versus undoped buffer for Gal-2-pHEA$_{42}$@AuNP$_{16}$ versus varying concentrations of SBA, WGA and *C. atrox* venom

Gal-2-pHEA$_{25}$@AuNP$_{30}$

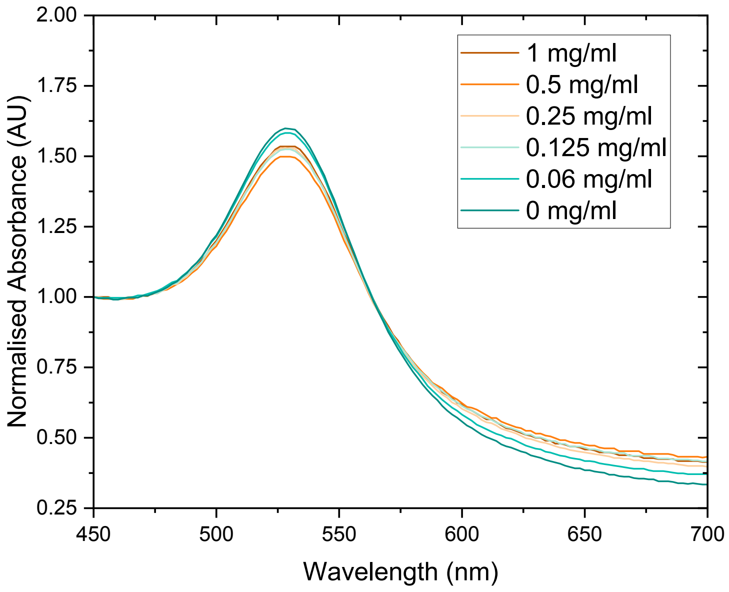

Figure S70 – Plot of normalised absorbance for Gal-2-pHEA$_{25}$@AuNP$_{30}$ versus varying concentrations of SBA

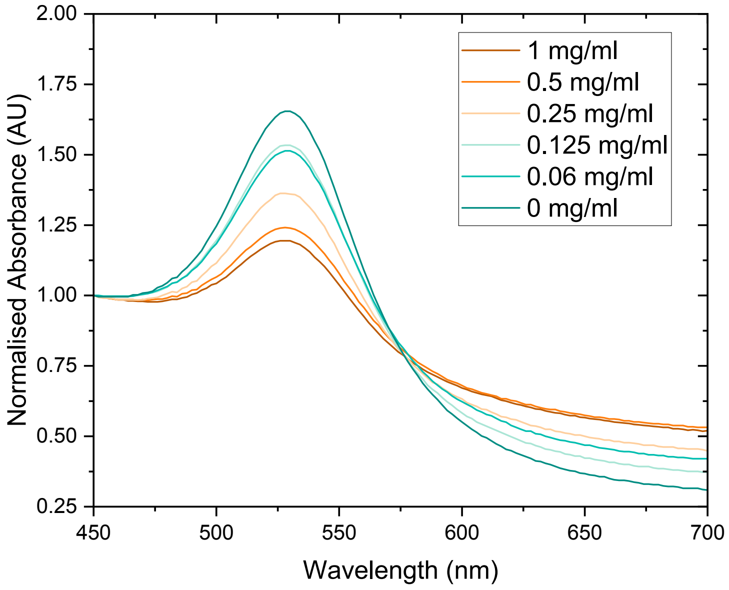

Figure S71 – Plot of normalised absorbance for Gal-2-pHEA$_{25}$@AuNP$_{30}$ versus varying concentrations of WGA

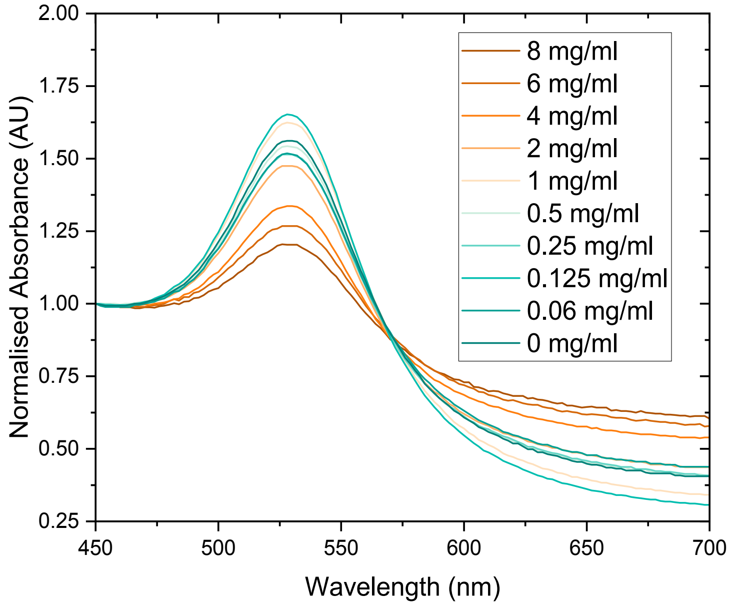

Figure S72 – Plot of normalised absorbance for Gal-2-pHEA$_{25}$@AuNP$_{30}$ versus varying concentrations of *C. atrox* venom

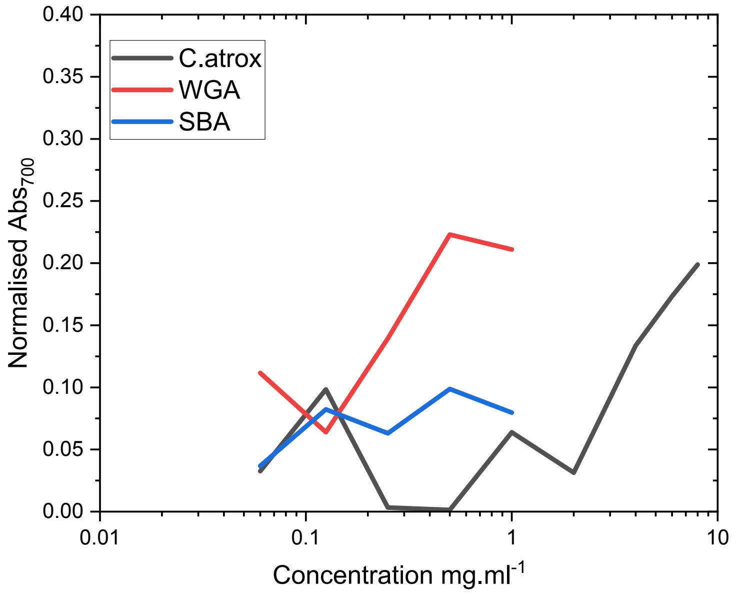

Figure S73 – Plot of normalised absorbance change versus undoped buffer for Gal-2-pHEA$_{25}$@AuNP$_{30}$ versus varying concentrations of SBA, WGA and *C. atrox* venom

Gal-2-pHEA$_{42}$@AuNP$_{30}$

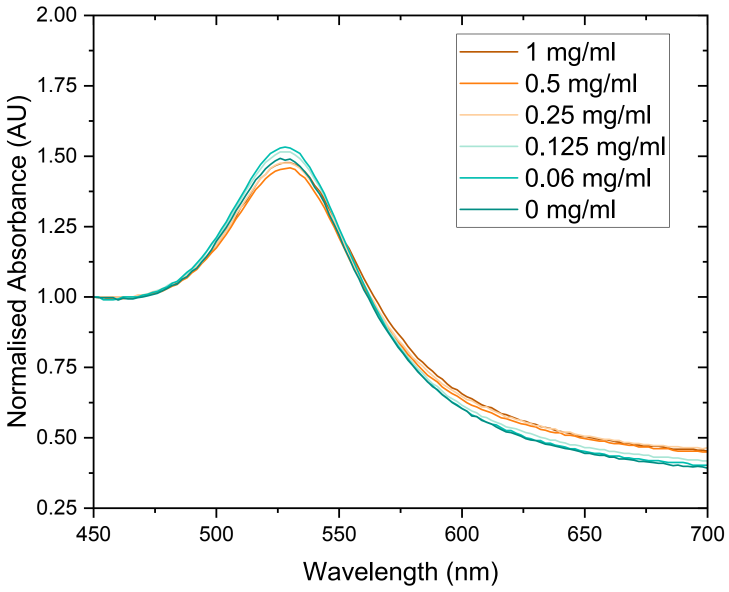

Figure S74 – Plot of normalised absorbance for Gal-2-pHEA$_{42}$@AuNP$_{30}$ versus varying concentrations of SBA

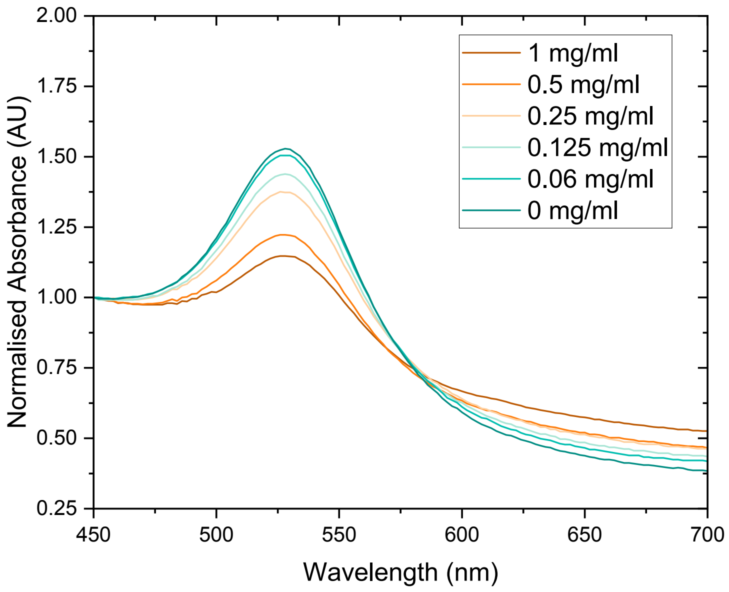

Figure S75 – Plot of normalised absorbance for Gal-2-pHEA$_{42}$@AuNP$_{30}$ versus varying concentrations of WGA

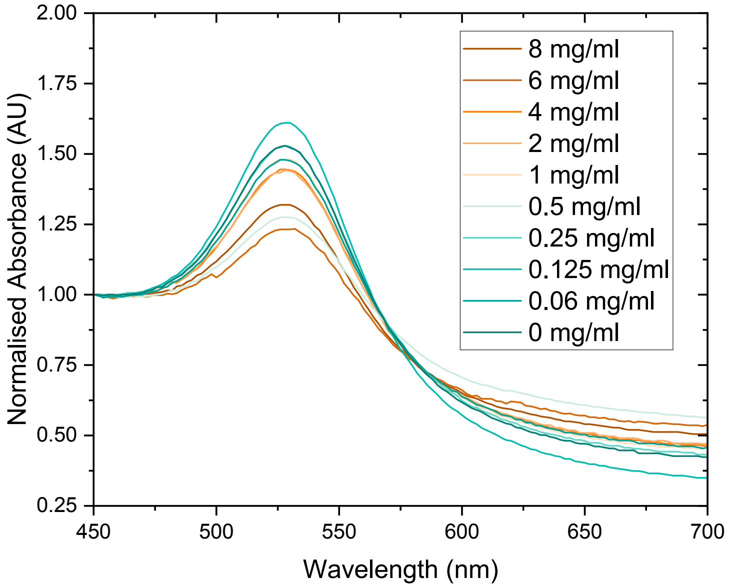

Figure S76 – Plot of normalised absorbance for Gal-2-pHEA$_{42}$@AuNP$_{30}$ versus varying concentrations of *C. atrox* venom

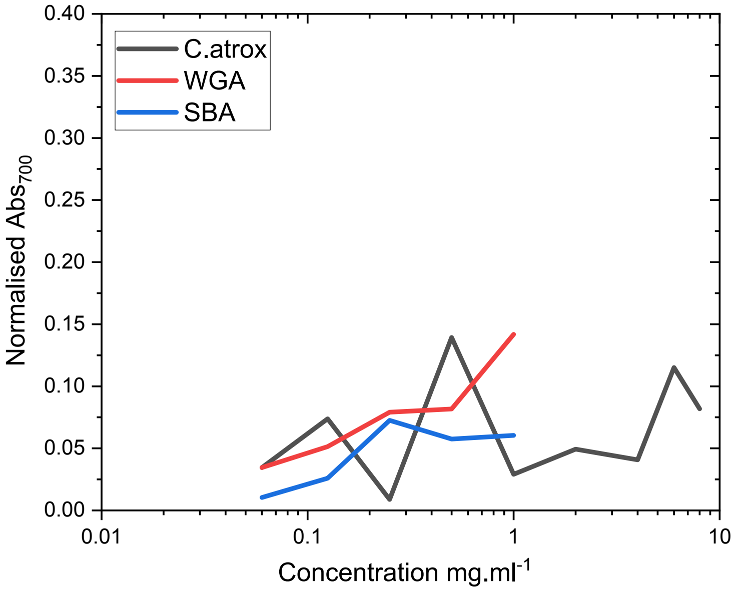

Figure S77 – Plot of normalised absorbance change versus undoped buffer for Gal-2-pHEA$_{42}$@AuNP$_{30}$ versus varying concentrations of SBA, WGA and *C. atrox* venom

Gal-2-pHEA$_{42}$@AuNP$_{40}$

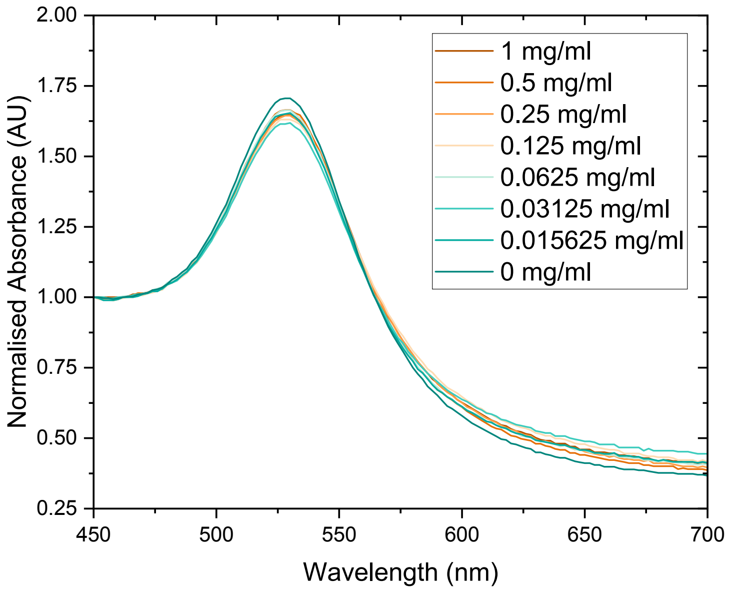

Figure S78 – Plot of normalised absorbance for Gal-2-pHEA$_{42}$@AuNP$_{40}$ versus varying concentrations of SBA

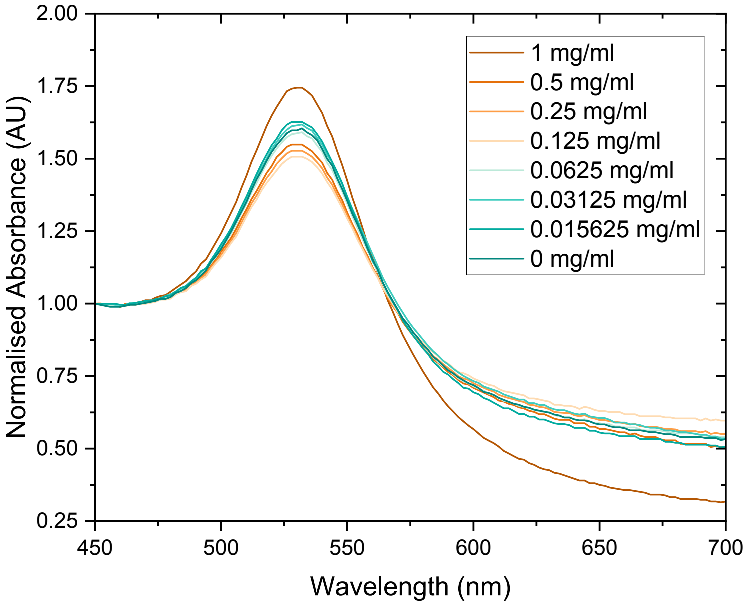

Figure S79 – Plot of normalised absorbance for Gal-2-pHEA$_{42}$@AuNP$_{40}$ versus varying concentrations of WGA

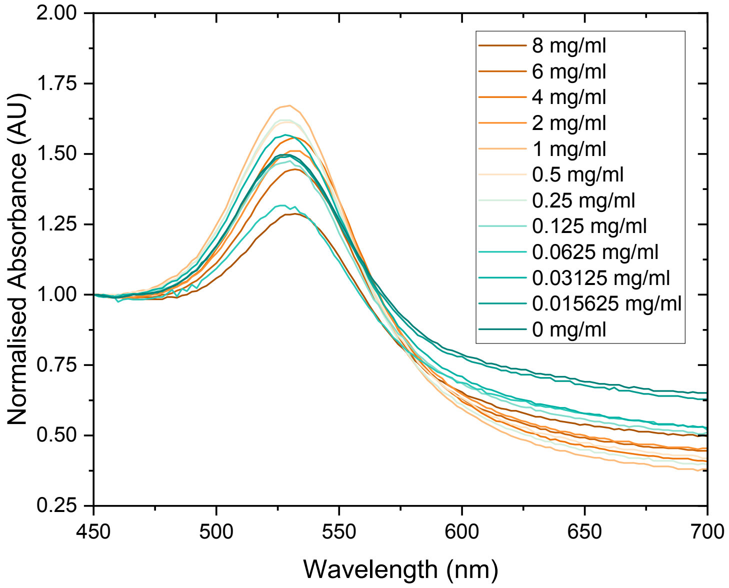

Figure S80 – Plot of normalised absorbance for Gal-2-pHEA$_{42}$@AuNP$_{40}$ versus varying concentrations of *C. atrox* venom

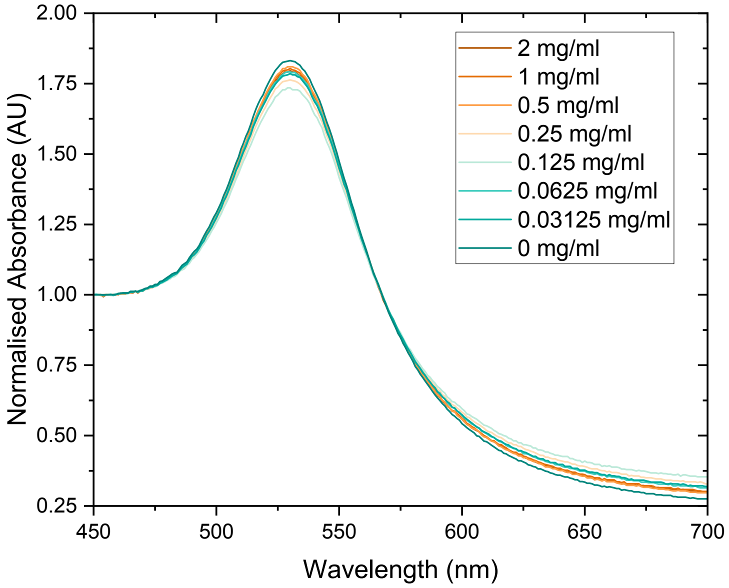

Figure S81 – Plot of normalised absorbance for Gal-2-pHEA$_{42}$@AuNP$_{40}$ versus varying concentrations of *N. naja* venom

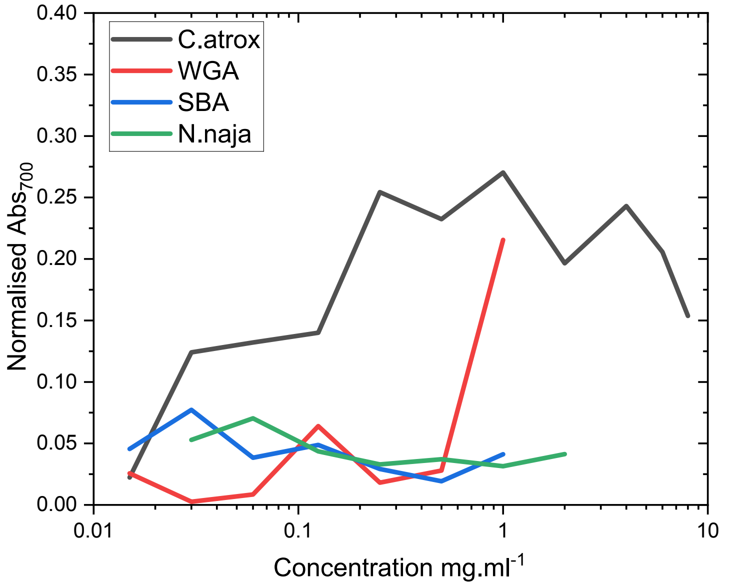

Figure S82 – Plot of normalised absorbance change versus undoped buffer for Gal-2-pHEA$_{42}$@AuNP$_{40}$ versus varying concentrations of SBA, WGA, *C. atrox* venom and *N. naja* venom

Glc-2-pHEA$_{42}$@AuNP$_{16}$

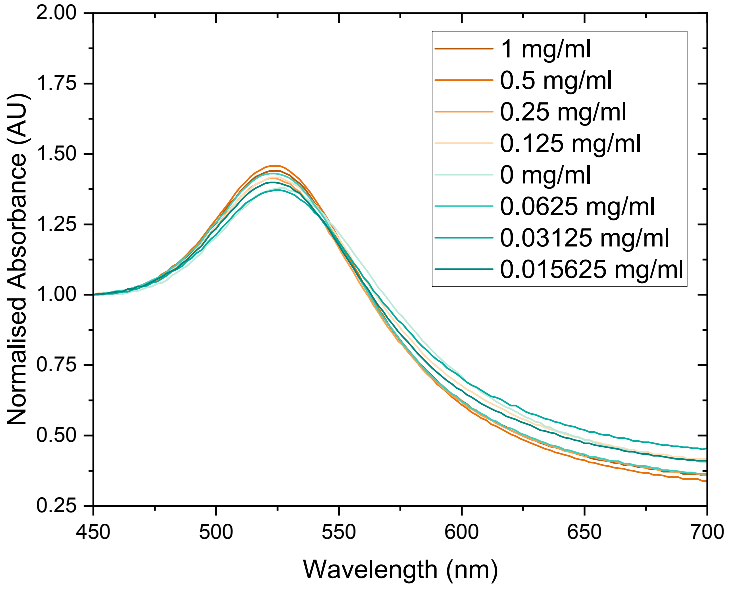

Figure S83 – Plot of normalised absorbance for Glc-2-pHEA$_{42}$@AuNP$_{16}$ versus varying concentrations of SBA

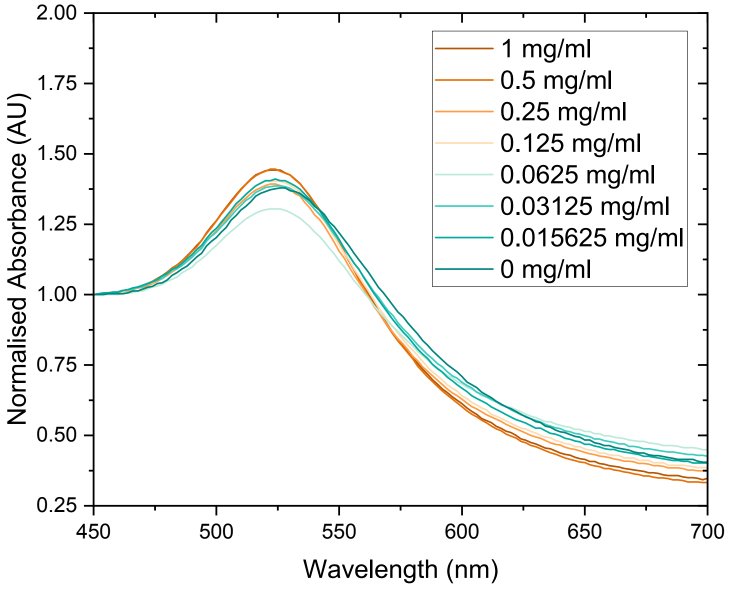

Figure S84 – Plot of normalised absorbance for Glc-2-pHEA$_{42}$@AuNP$_{16}$ versus varying concentrations of WGA

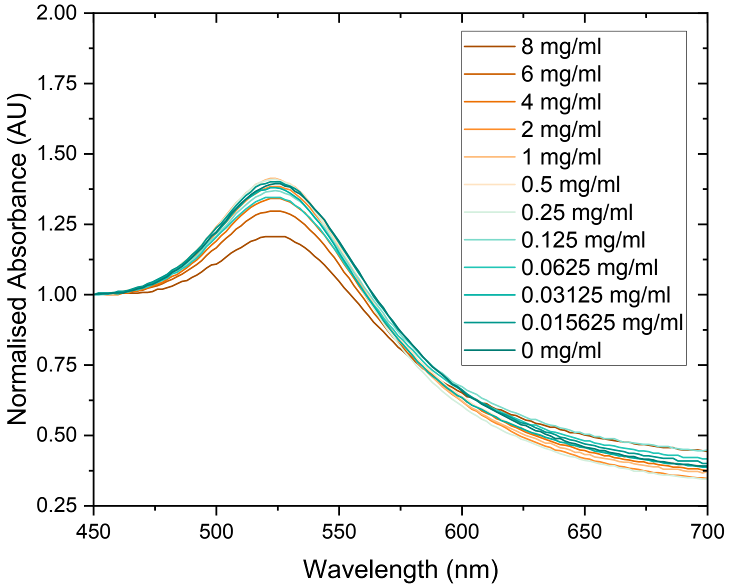

Figure S85 – Plot of normalised absorbance for Glc-2-pHEA$_{42}$@AuNP$_{16}$ versus varying concentrations of *C. atrox* venom

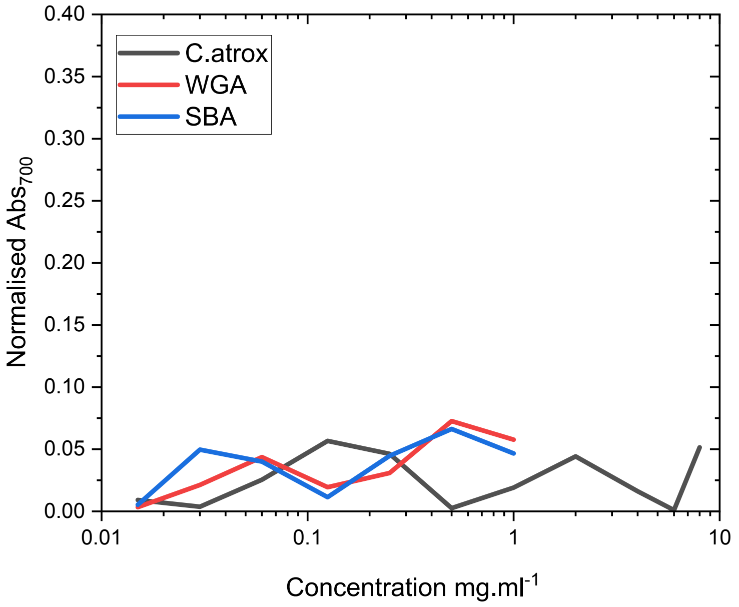

Figure S86 – Plot of normalised absorbance change versus undoped buffer for Glc-2-pHEA$_{42}$@AuNP$_{16}$ versus varying concentrations of SBA, WGA and *C. atrox* venom

Glc-2-pHEA$_{42}$@AuNP$_{40}$

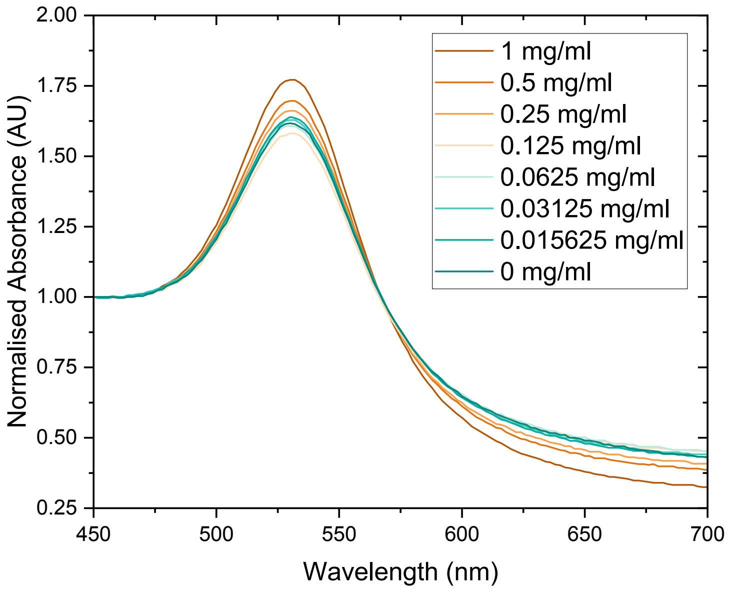

Figure S87 – Plot of normalised absorbance for Glc-2-pHEA$_{42}$@AuNP$_{40}$ versus varying concentrations of SBA

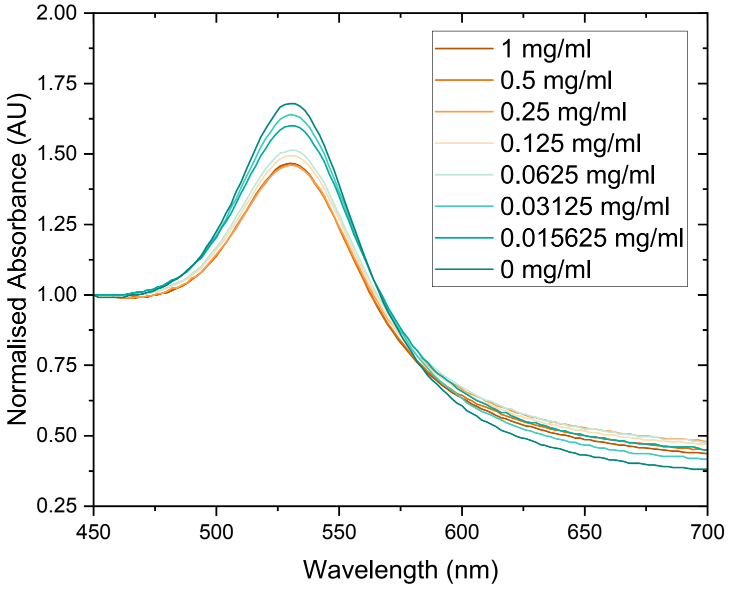

Figure S88 – Plot of normalised absorbance for Glc-2-pHEA$_{42}$@AuNP$_{40}$ versus varying concentrations of WGA

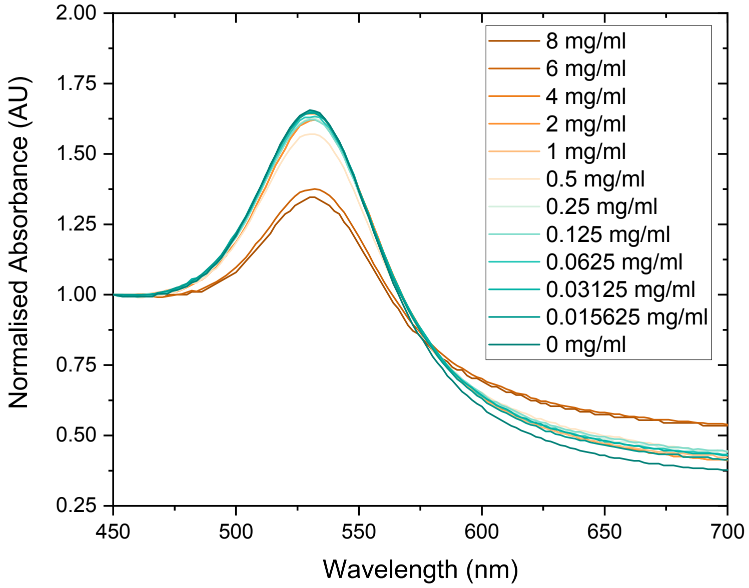

Figure S89 – Plot of normalised absorbance for Glc-2-pHEA$_{42}$@AuNP$_{40}$ versus varying concentrations of *C. atrox* venom

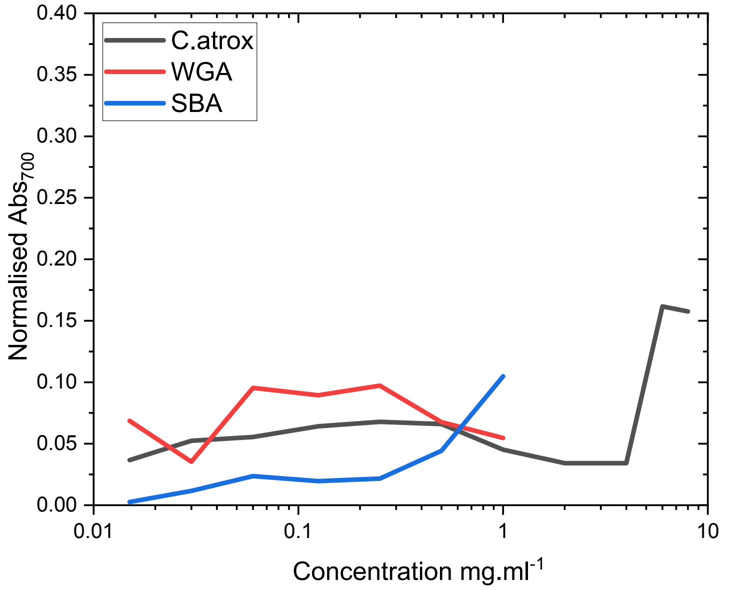

Figure S90 – Plot of normalised absorbance change versus undoped buffer for Glc-2-pHEA$_{42}$@AuNP$_{40}$ versus varying concentrations of SBA, WGA and *C. atrox* venom

Man-2-pHEA$_{42}$@AuNP$_{16}$

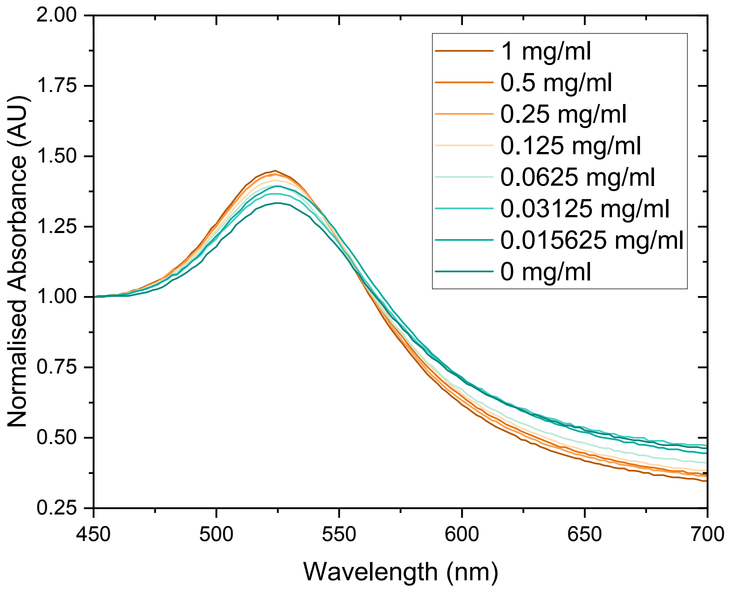

Figure S91 – Plot of normalised absorbance for Man-2-pHEA$_{42}$@AuNP$_{16}$ versus varying concentrations of SBA

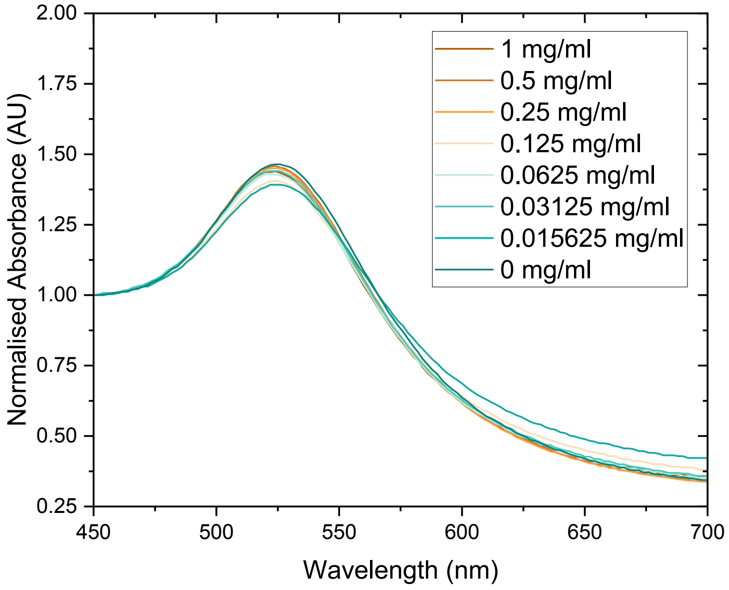

Figure S92 – Plot of normalised absorbance for Man-2-pHEA$_{42}$@AuNP$_{16}$ versus varying concentrations of WGA

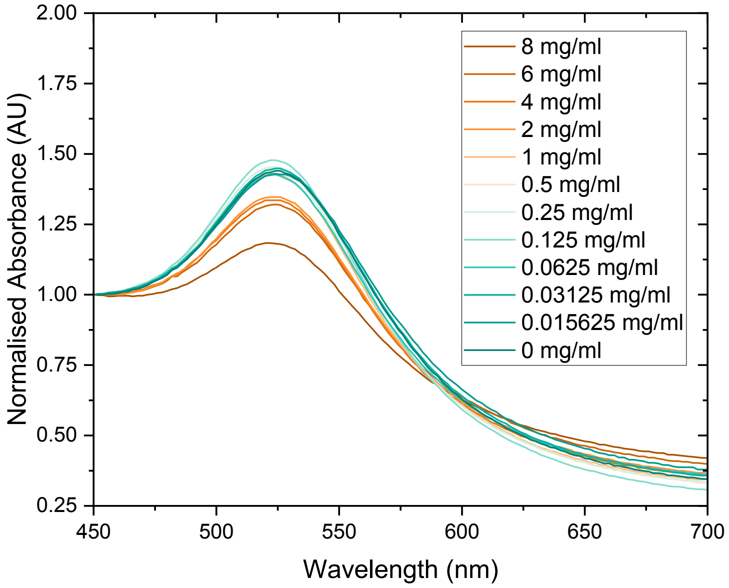

Figure S93 – Plot of normalised absorbance for Man-2-pHEA$_{42}$@AuNP$_{16}$ versus varying concentrations of *C. atrox* venom

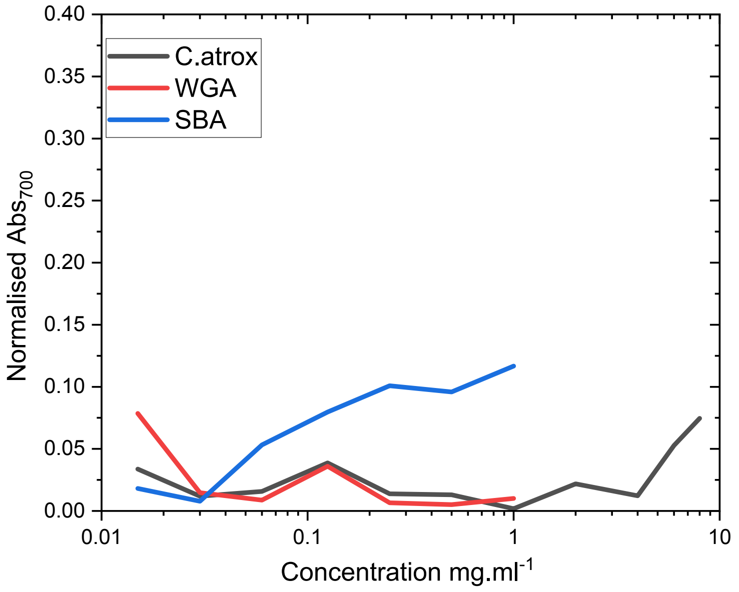

Figure S94 – Plot of normalised absorbance change versus undoped buffer for Man-2-pHEA$_{42}$@AuNP$_{16}$ versus varying concentrations of SBA, WGA and *C. atrox* venom

Man-2-pHEA$_{42}$@AuNP$_{40}$

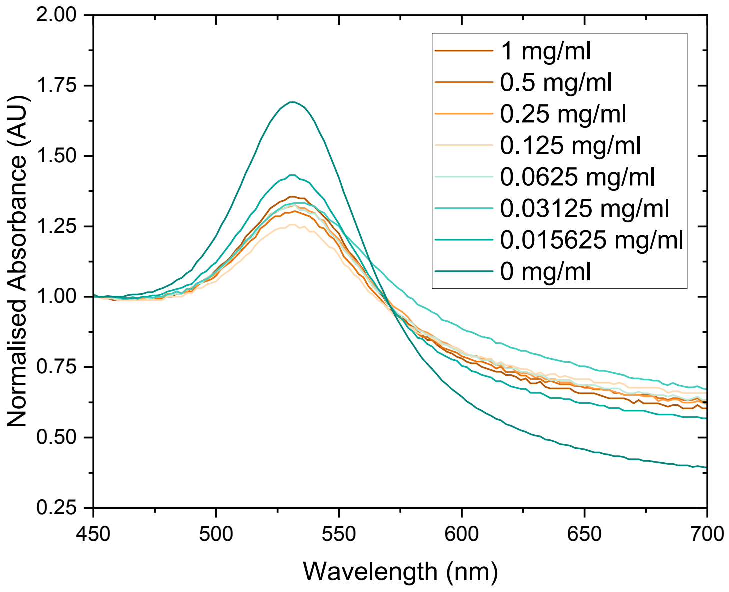

Figure S95 – Plot of normalised absorbance for Man-2-pHEA$_{42}$@AuNP$_{40}$ versus varying concentrations of SBA

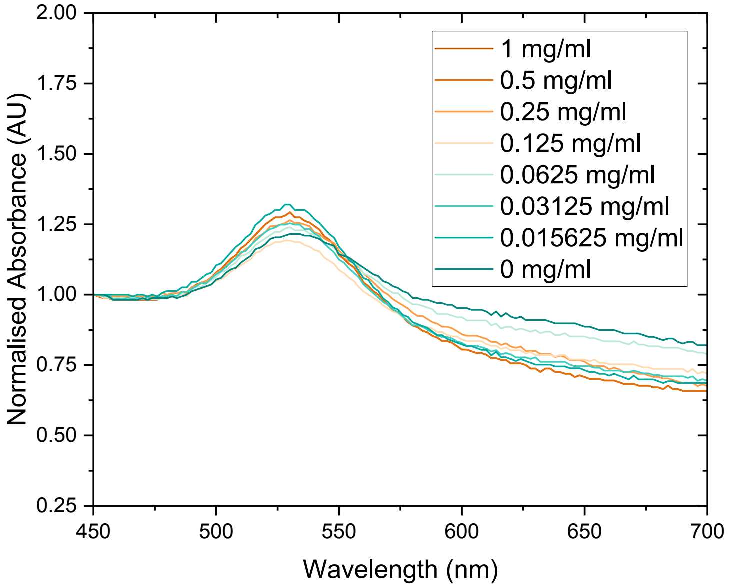

Figure S96 – Plot of normalised absorbance for Man-2-pHEA$_{42}$@AuNP$_{40}$ versus varying concentrations of WGA

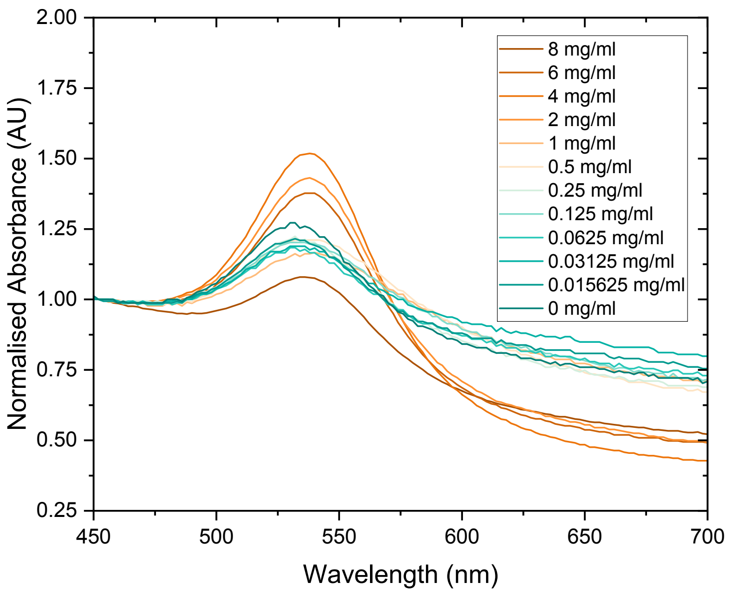

Figure S97 – Plot of normalised absorbance for Man-2-pHEA$_{42}$@AuNP$_{40}$ versus varying concentrations of *C. atrox* venom

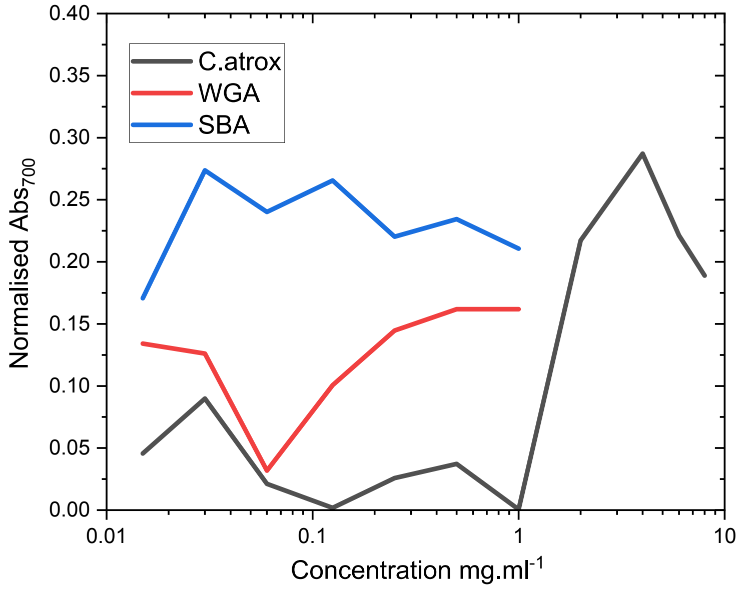

Figure S98 – Plot of normalised absorbance change versus undoped buffer for Man-2-pHEA$_{42}$@AuNP$_{40}$ versus varying concentrations of SBA, WGA and *C. atrox* venom

Lac-1-pHEA$_{25}$@AuNP$_{16}$

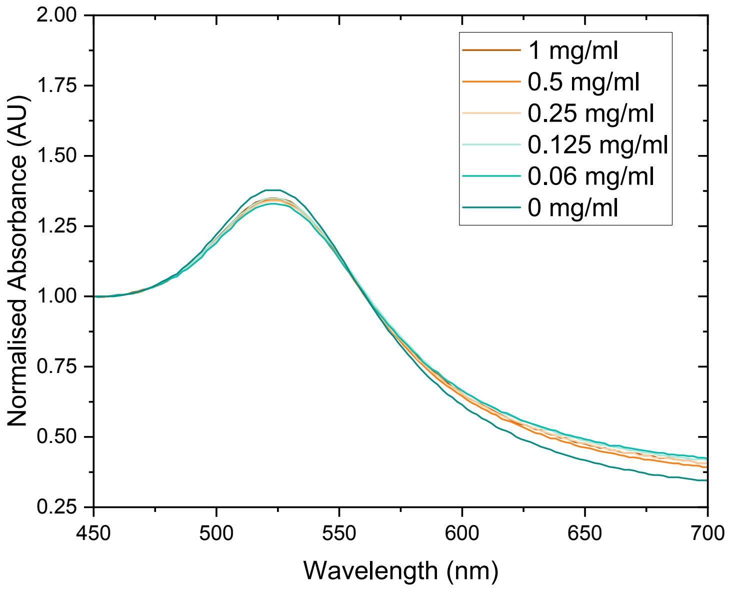

Figure S99 – Plot of normalised absorbance for Lac-1-pHEA$_{25}$@AuNP$_{16}$ versus varying concentrations of SBA

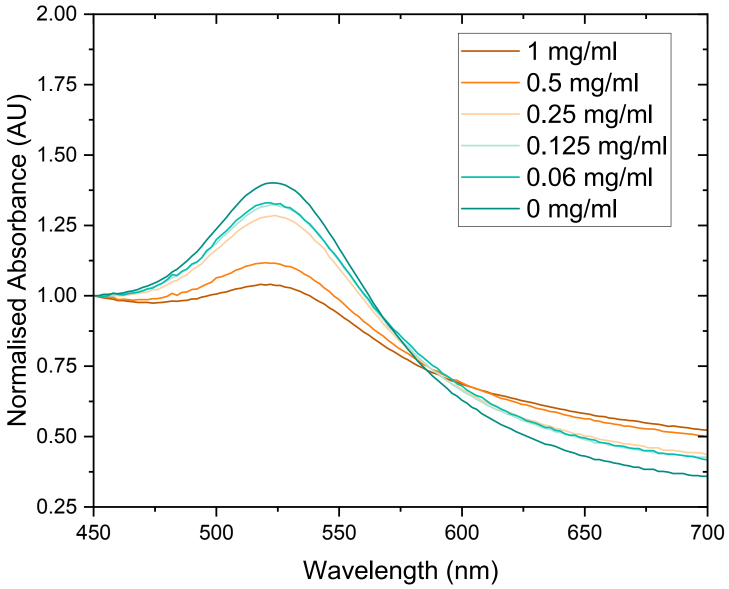

Figure S100 – Plot of normalised absorbance for Lac-1-pHEA$_{25}$@AuNP$_{16}$ versus varying concentrations of WGA

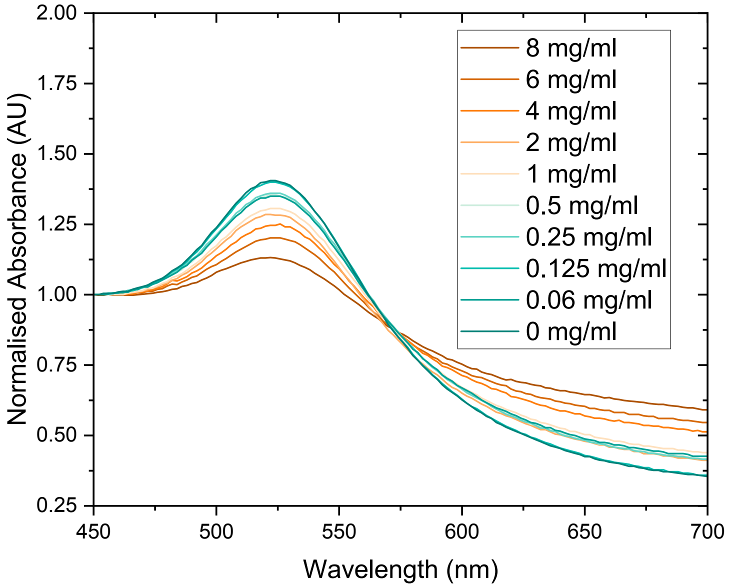

Figure S101 – Plot of normalised absorbance for Lac-1-pHEA$_{25}$@AuNP$_{16}$ versus varying concentrations of *C. atrox* venom

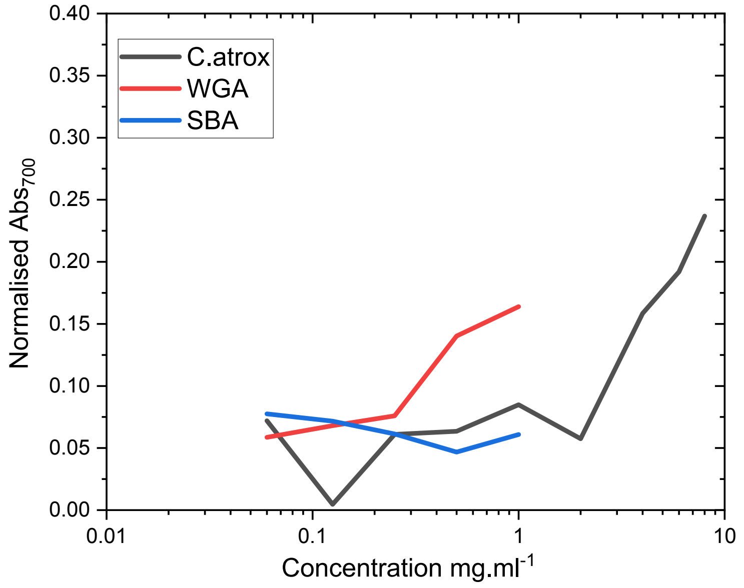

Figure S102 – Plot of normalised absorbance change versus undoped buffer for Lac-1-pHEA$_{25}$@AuNP$_{16}$ versus varying concentrations of SBA, WGA and *C. atrox* venom

Lac-1-pHEA$_{42}$@AuNP$_{16}$

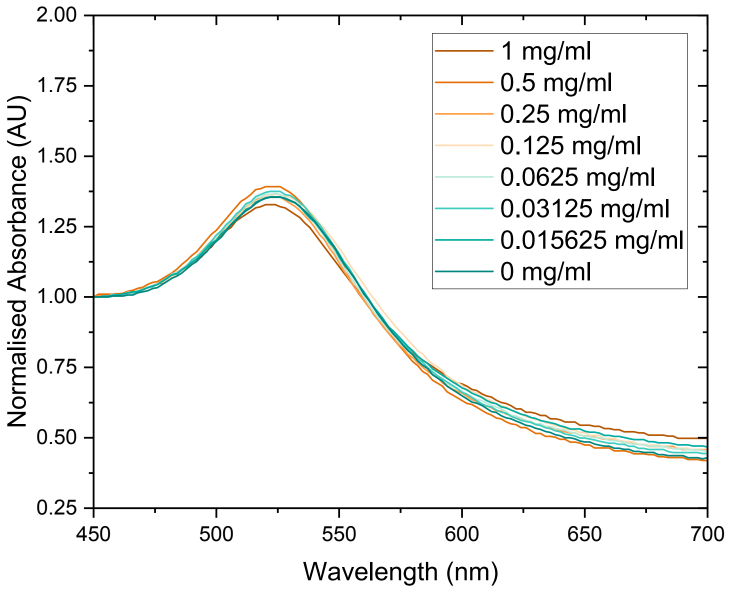

Figure S103 – Plot of normalised absorbance for Lac-1-pHEA$_{42}$@AuNP$_{16}$ versus varying concentrations of SBA

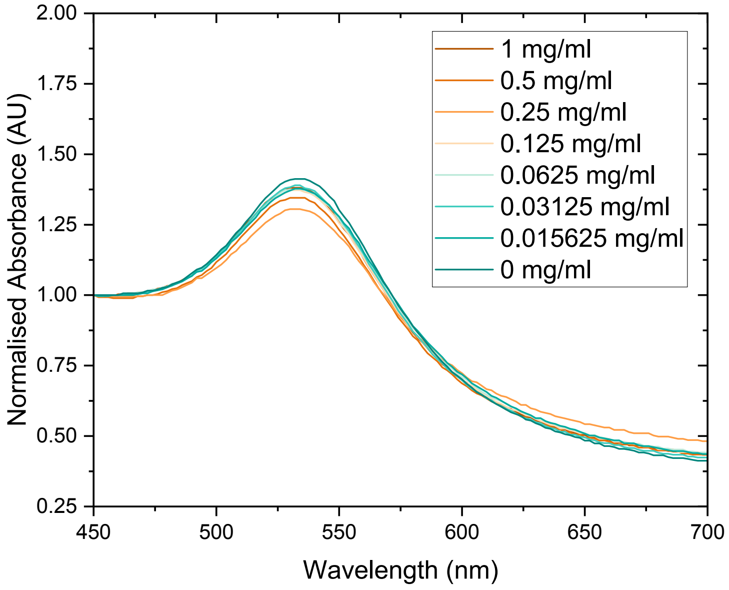

Figure S104 – Plot of normalised absorbance for Lac-1-pHEA$_{42}$@AuNP$_{16}$ versus varying concentrations of WGA

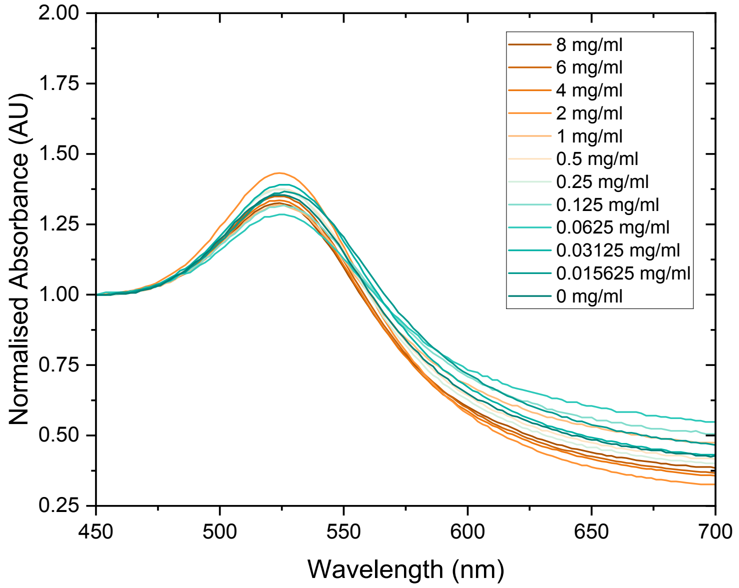

Figure S105 – Plot of normalised absorbance for Lac-1-pHEA$_{42}$@AuNP$_{16}$ versus varying concentrations of *C. atrox* venom

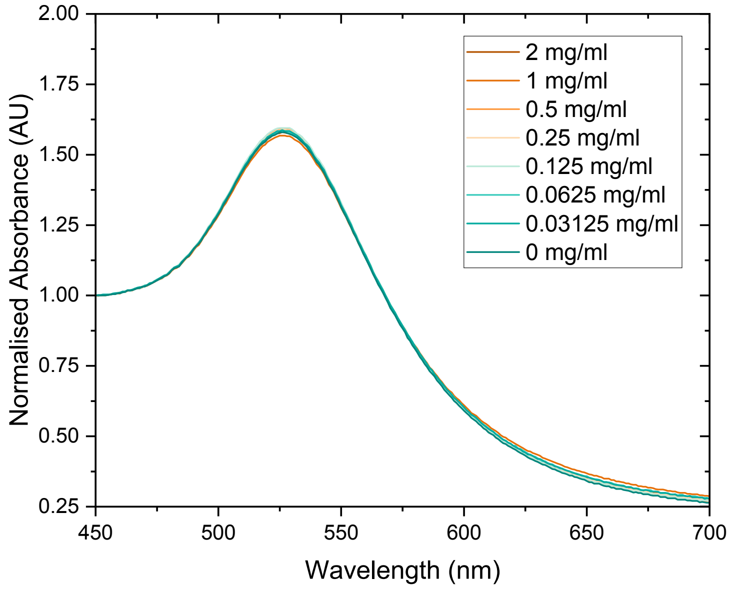

Figure S106 – Plot of normalised absorbance for Lac-1-pHEA$_{42}$@AuNP$_{16}$ versus varying concentrations of *N. naja* venom

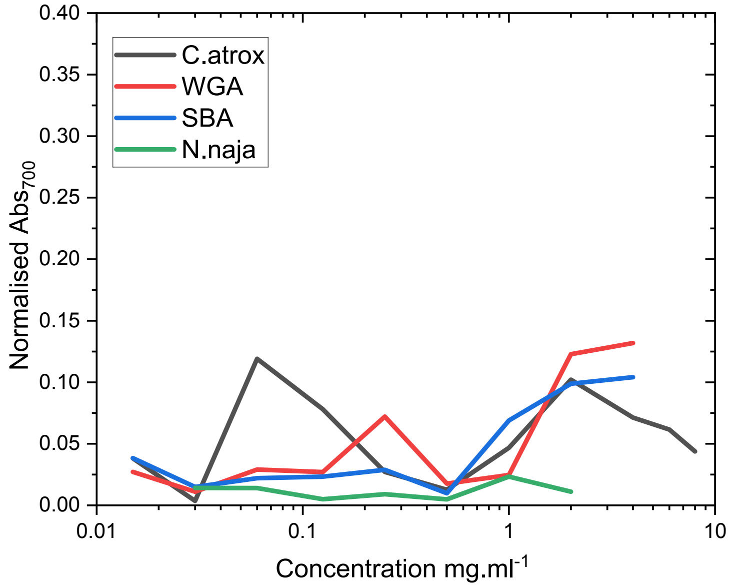

Figure S107 – Plot of normalised absorbance change versus undoped buffer for Lac-1-pHEA$_{42}$@AuNP$_{16}$ versus varying concentrations of SBA, WGA. *C. atrox* venom and *N. naja venom*

Lac-1-pHEA$_{25}$@AuNP$_{30}$

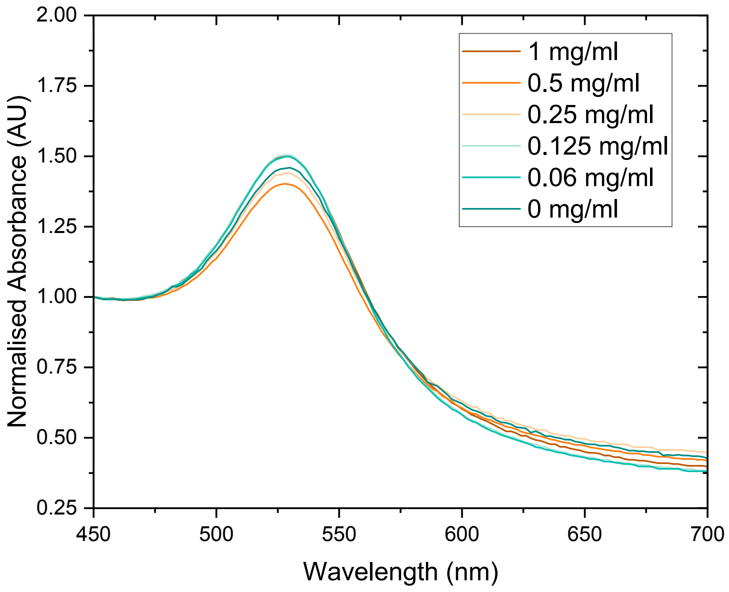

Figure S108 – Plot of normalised absorbance for Lac-1-pHEA$_{25}$@AuNP$_{30}$ versus varying concentrations of SBA

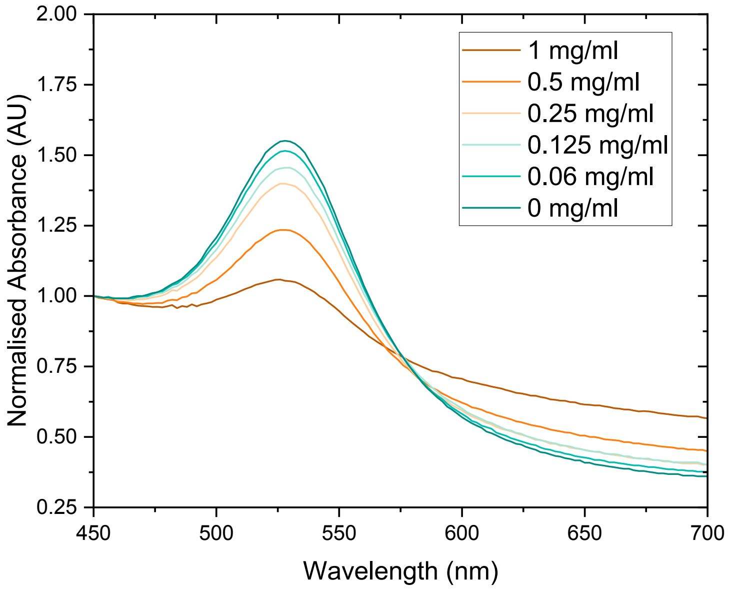

Figure S109 – Plot of normalised absorbance for Lac-1-pHEA$_{25}$@AuNP$_{30}$ versus varying concentrations of WGA

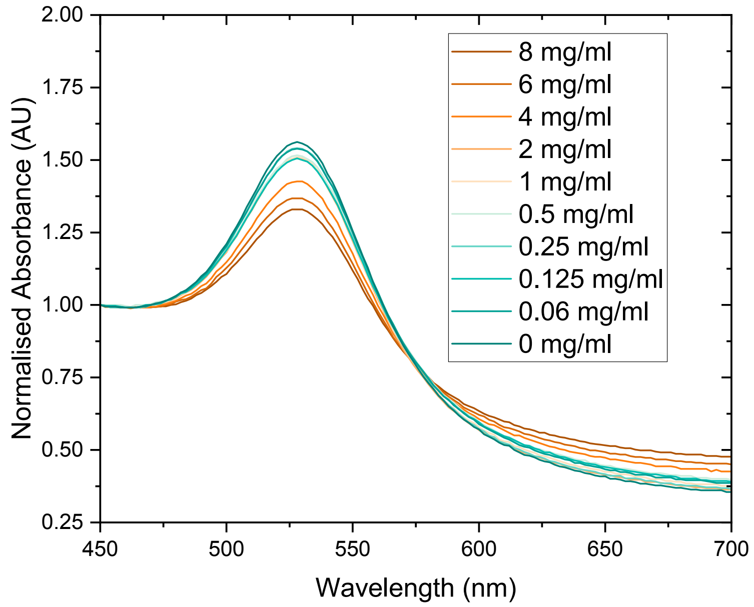

Figure S110 – Plot of normalised absorbance for Lac-1-pHEA$_{25}$@AuNP$_{30}$ versus varying concentrations of *C. atrox* venom

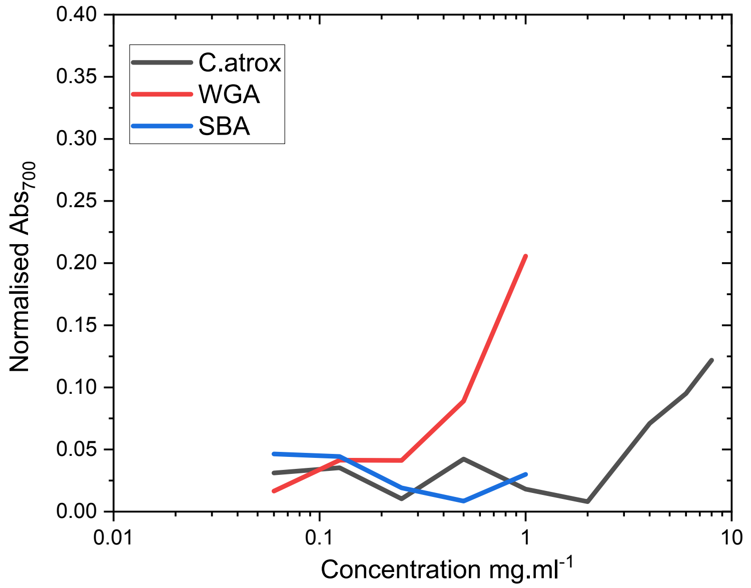

Figure S111 – Plot of normalised absorbance change versus undoped buffer for Lac-1-pHEA$_{25}$@AuNP$_{30}$ versus varying concentrations of SBA, WGA and *C. atrox* venom

Lac-1-pHEA$_{52}$@AuNP$_{40}$

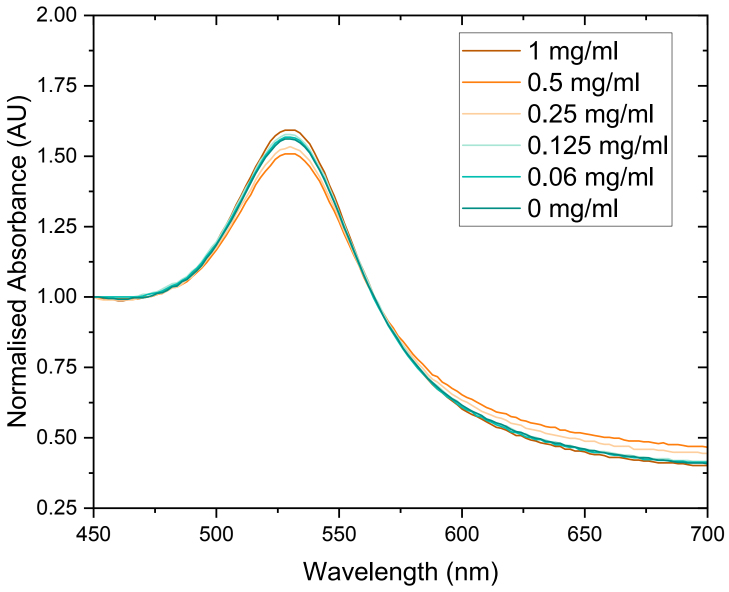

Figure S112 – Plot of normalised absorbance for Lac-1-pHEA$_{52}$@AuNP$_{40}$ versus varying concentrations of SBA

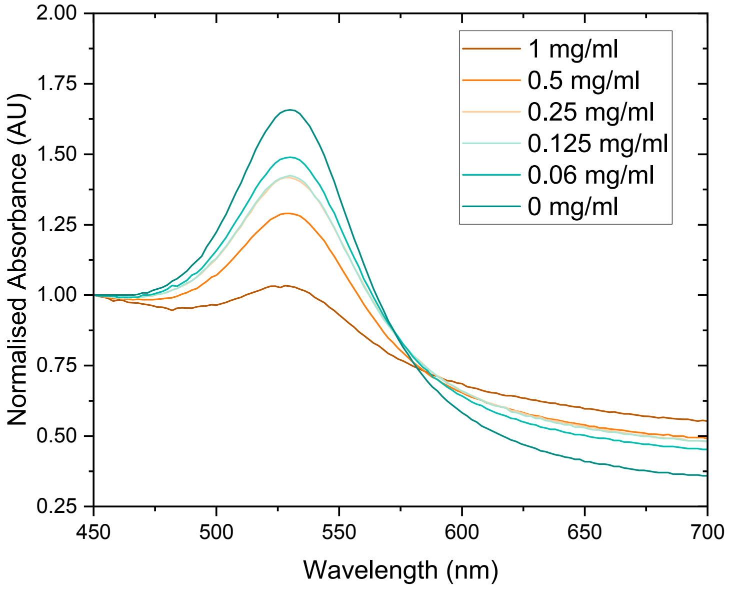

Figure S113 – Plot of normalised absorbance for Lac-1-pHEA$_{52}$@AuNP$_{40}$ versus varying concentrations of WGA

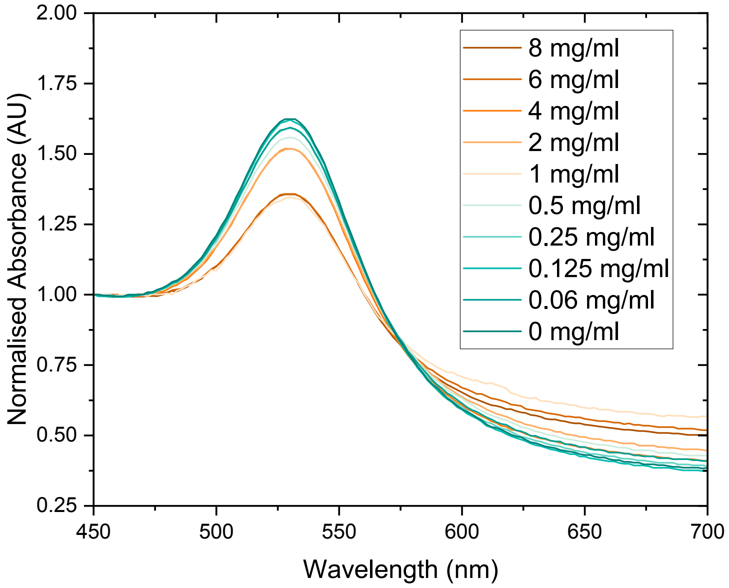

Figure S114 – Plot of normalised absorbance for Lac-1-pHEA$_{52}$@AuNP$_{40}$ versus varying concentrations of *C. atrox* venom

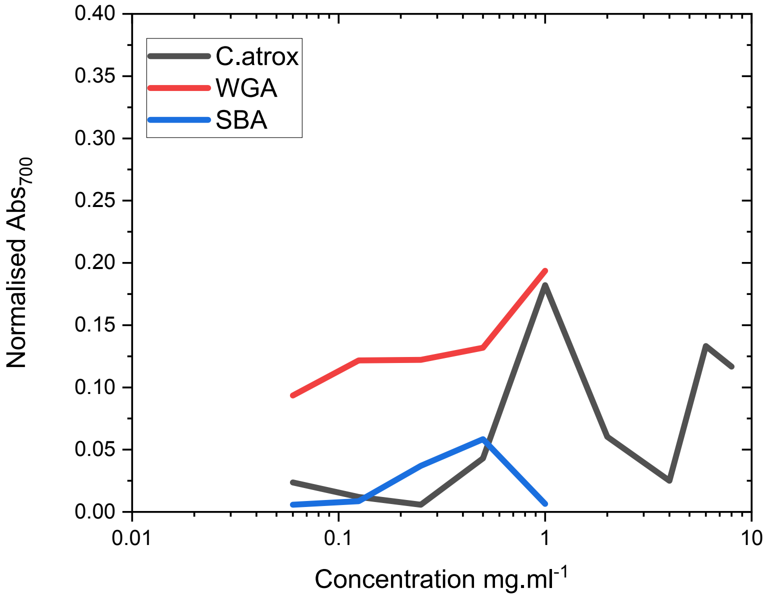

Figure S115 – Plot of normalised absorbance change versus undoped buffer for Lac-1-pHEA$_{52}$@AuNP$_{40}$ versus varying concentrations of SBA, WGA and *C. atrox* venom

XPS Data

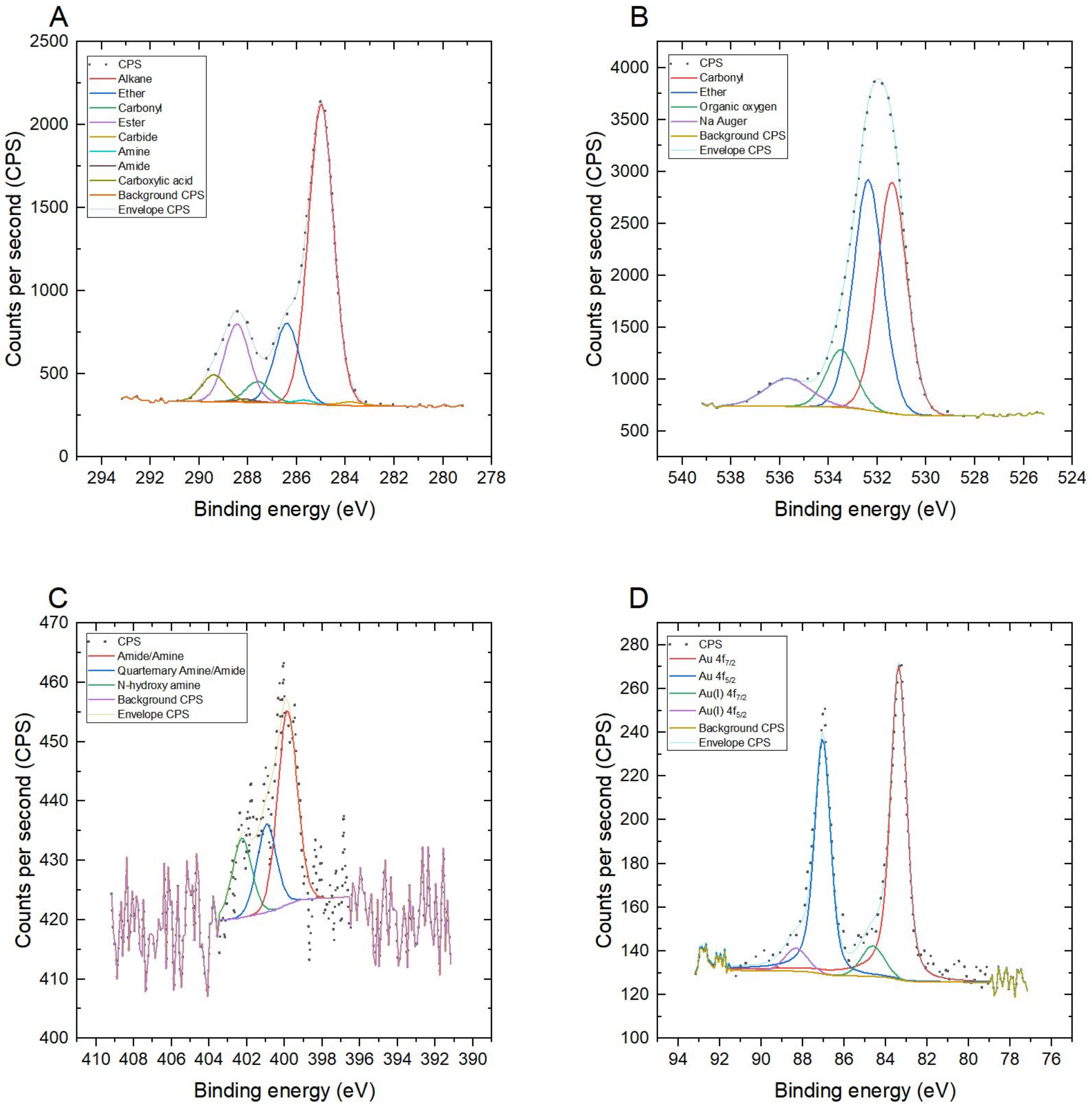

Figure S116 – XPS of $AuNP_{16}$ (unfunctionalized (citrate-stabilised) AuNP16) A) C 1*s* B) O 1*s* C) N 1*s* and D) Au 4*f*

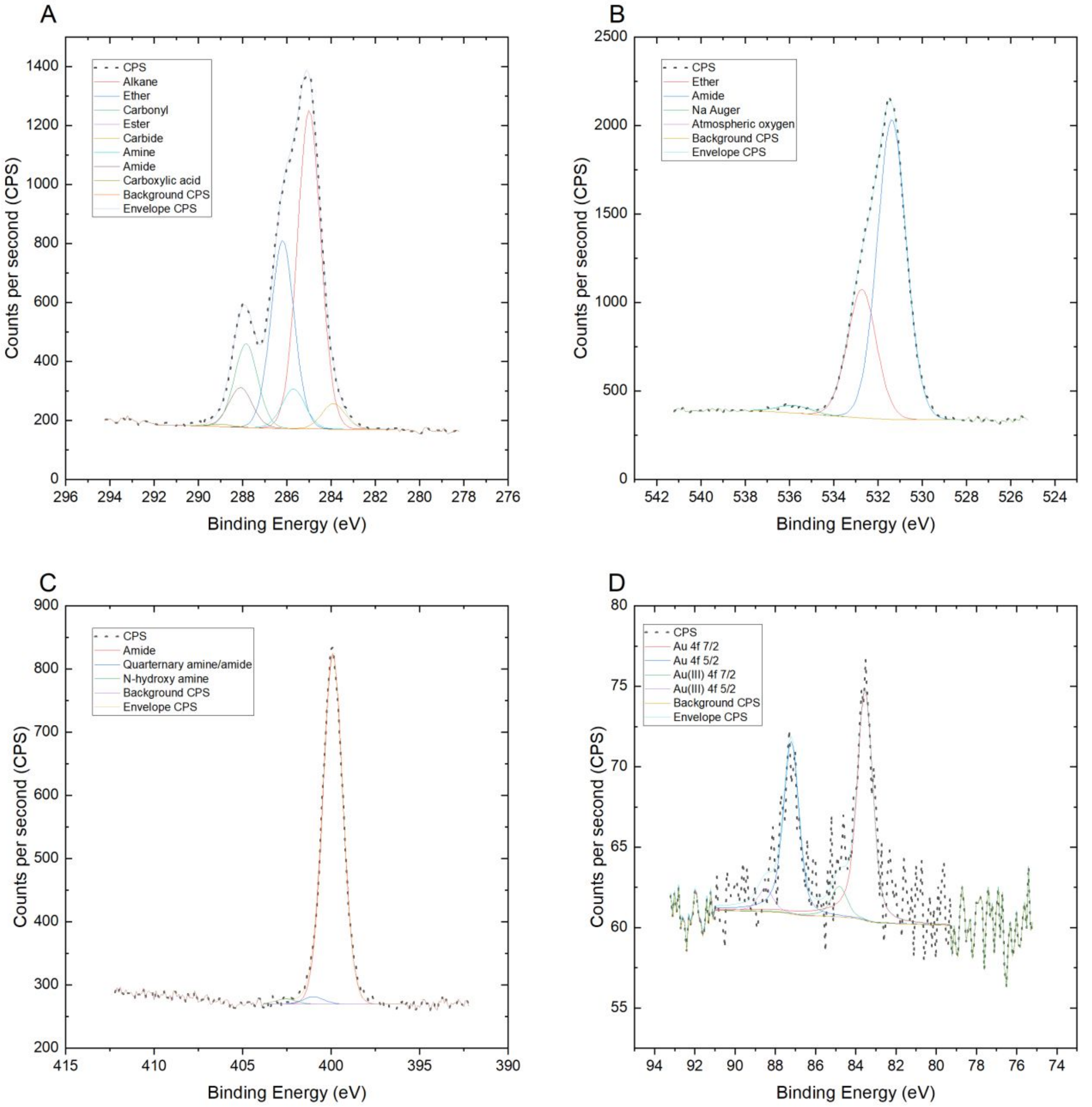

Figure S117 – XPS of GalNAc-1-pHEA42@AuNP16 A) C 1*s* B) O 1*s* C) N 1*s* and D) Au 4*f*

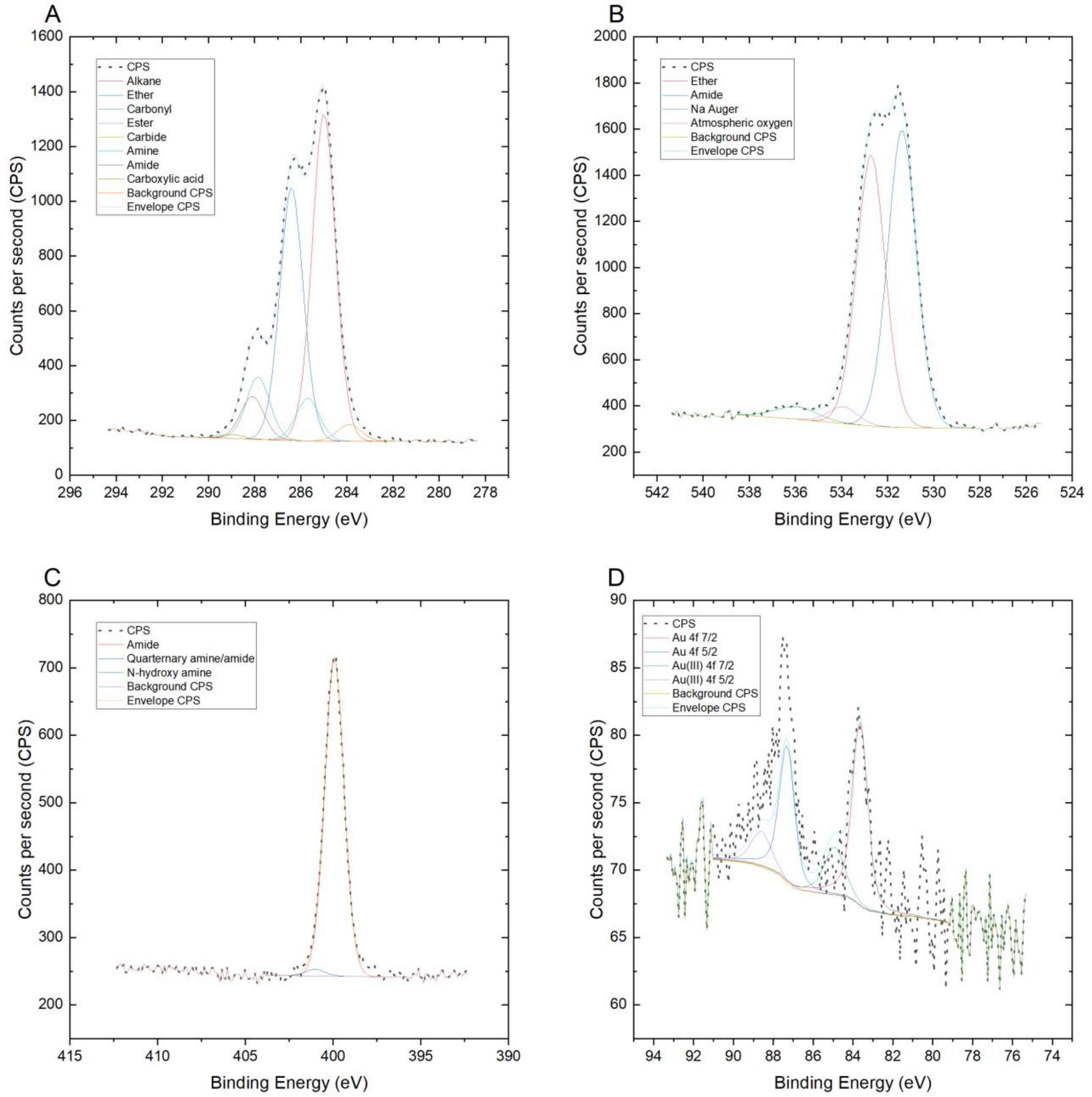

Figure S118 – XPS of GalNAc-1-pHEA42@AuNP40 A) C 1*s* B) O 1*s* C) N 1*s* and D) Au 4*f*

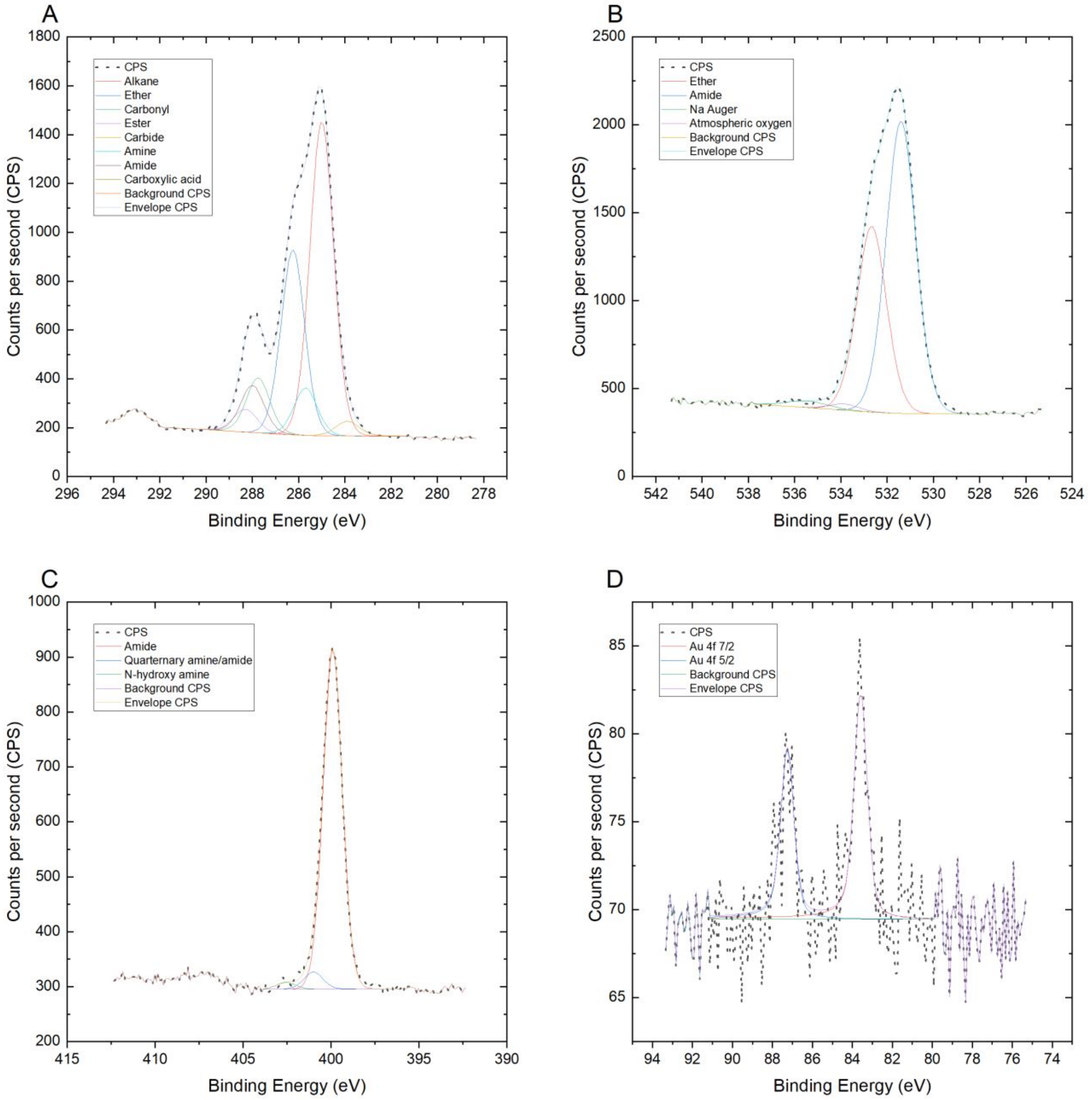

Figure S119 – XPS of Gal-1-pHEA42@AuNP16 A) C 1*s* B) O 1*s* C) N 1*s* and D) Au 4*f*

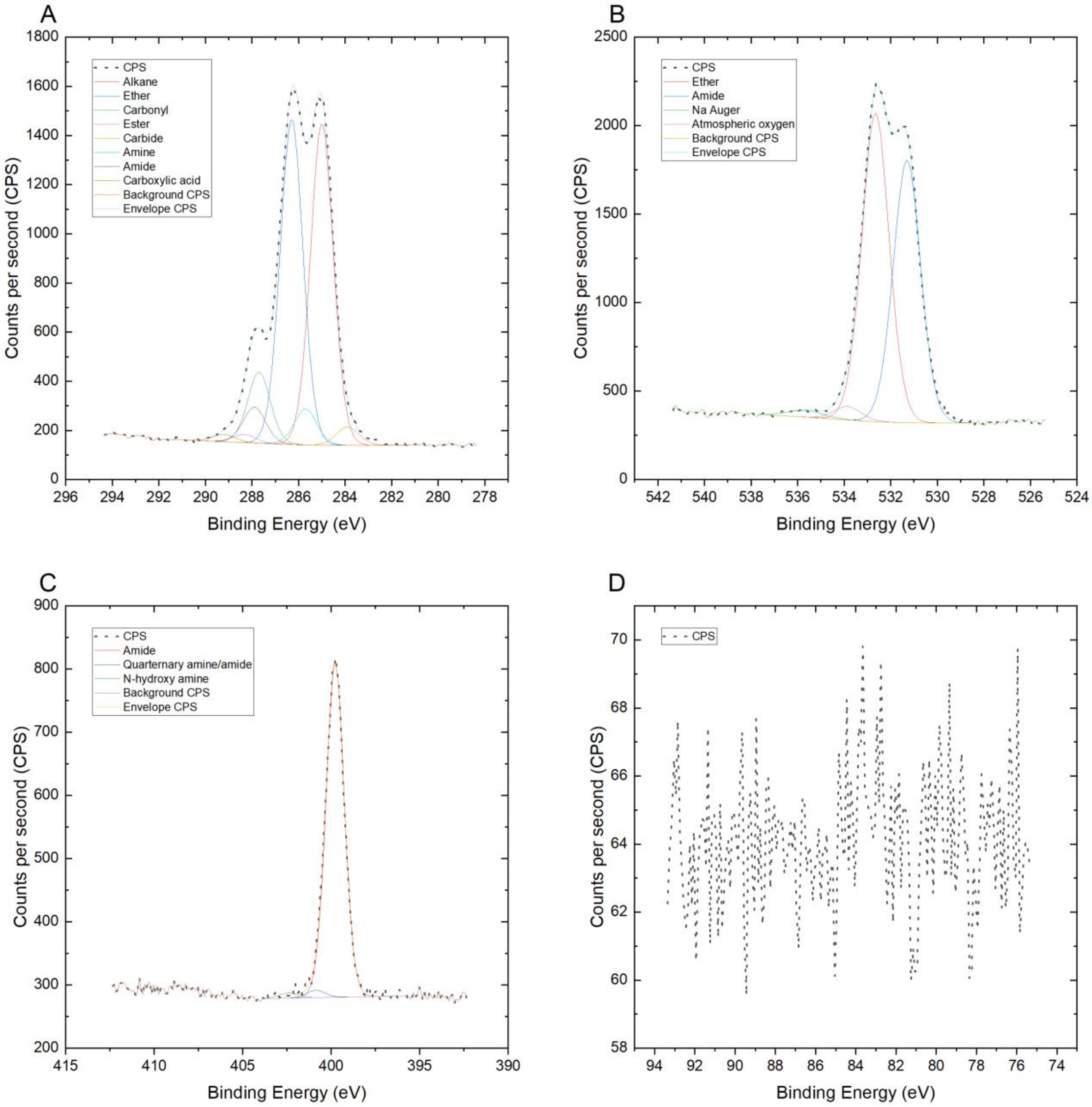

Figure S120 – XPS of Gal-1-pHEA42@AuNP40 A) C 1*s* B) O 1*s* C) N 1*s* and D) Au 4*f*

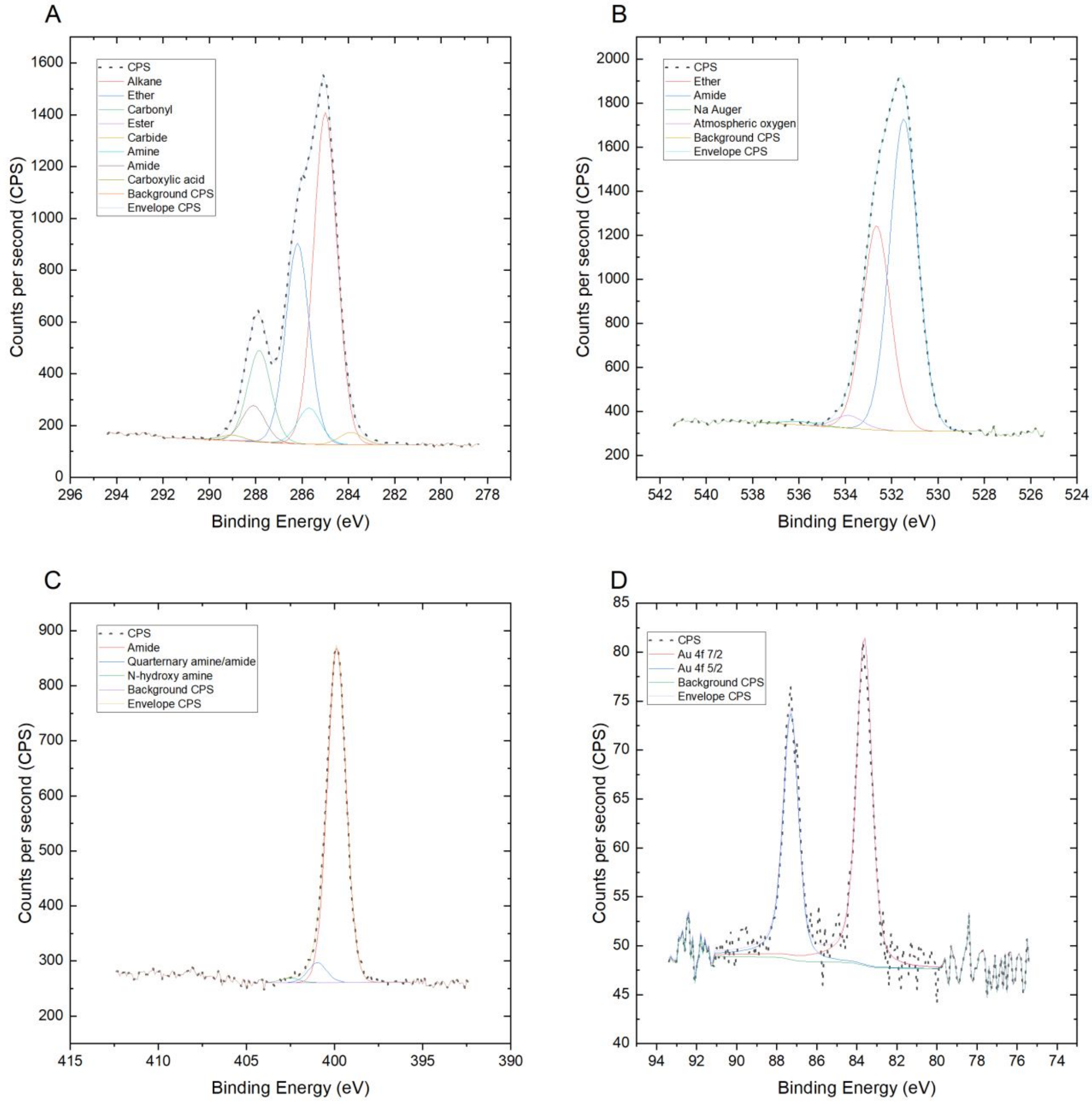

Figure S121 – XPS of Gal-2-pHEA25@AuNP16 A) C 1*s* B) O 1*s* C) N 1*s* and D) Au 4*f*

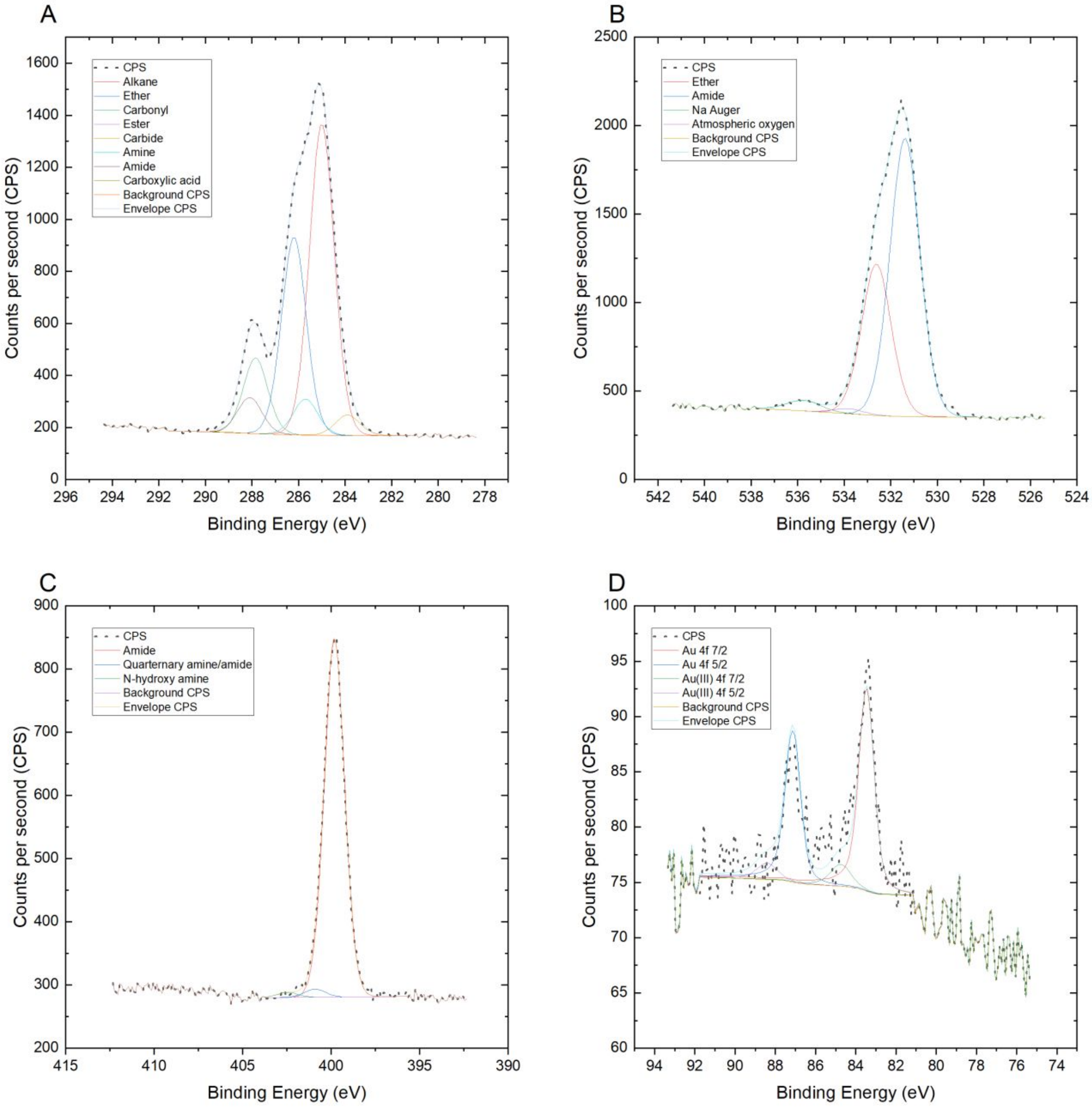

Figure S122 – XPS of Gal-2-pHEA42@AuNP16 A) C 1*s* B) O 1*s* C) N 1*s* and D) Au 4*f*

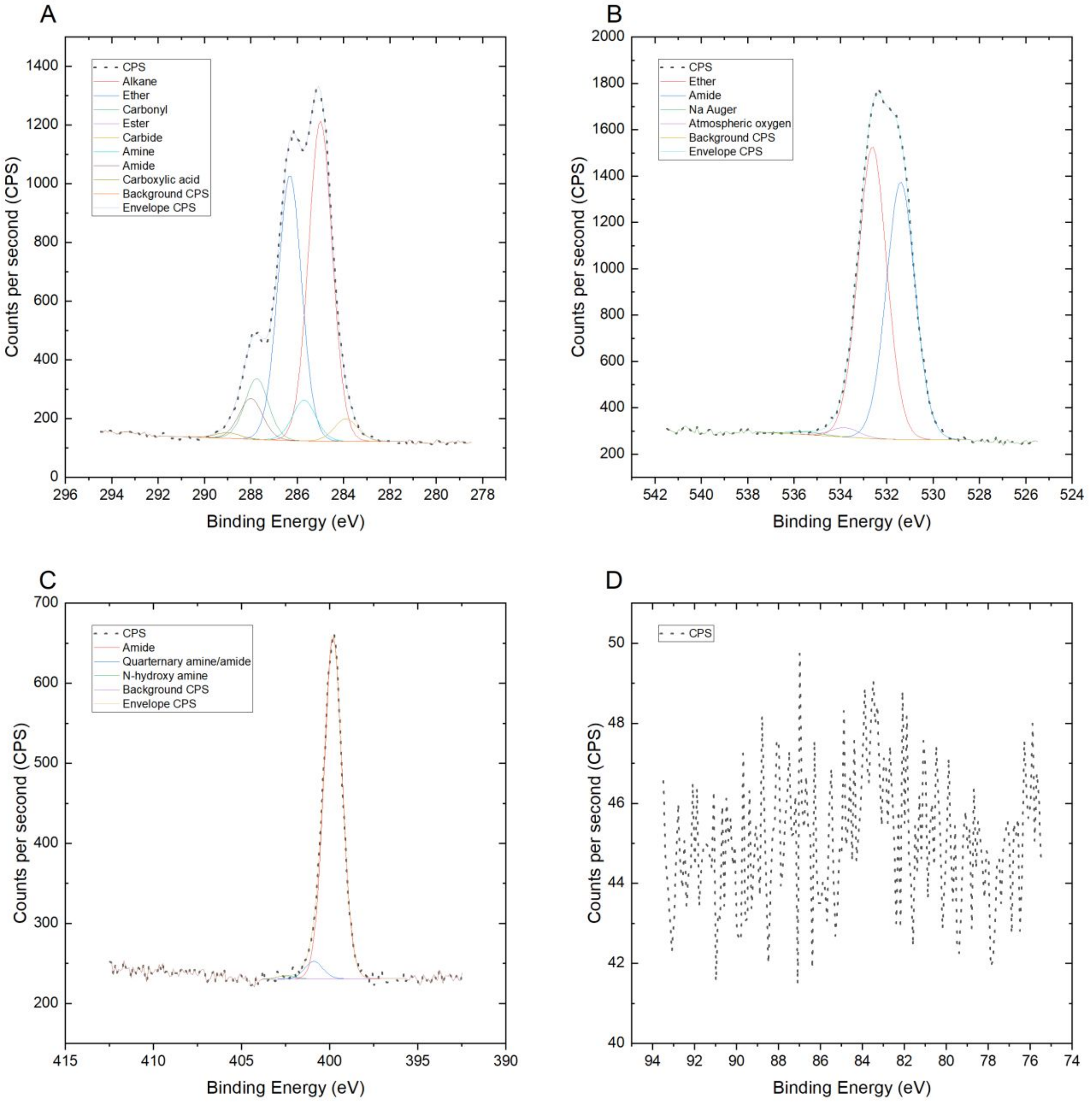

Figure S123 – XPS of Gal-2-pHEA25@AuNP30 A) C 1*s* B) O 1*s* C) N 1*s* and D) Au 4*f*

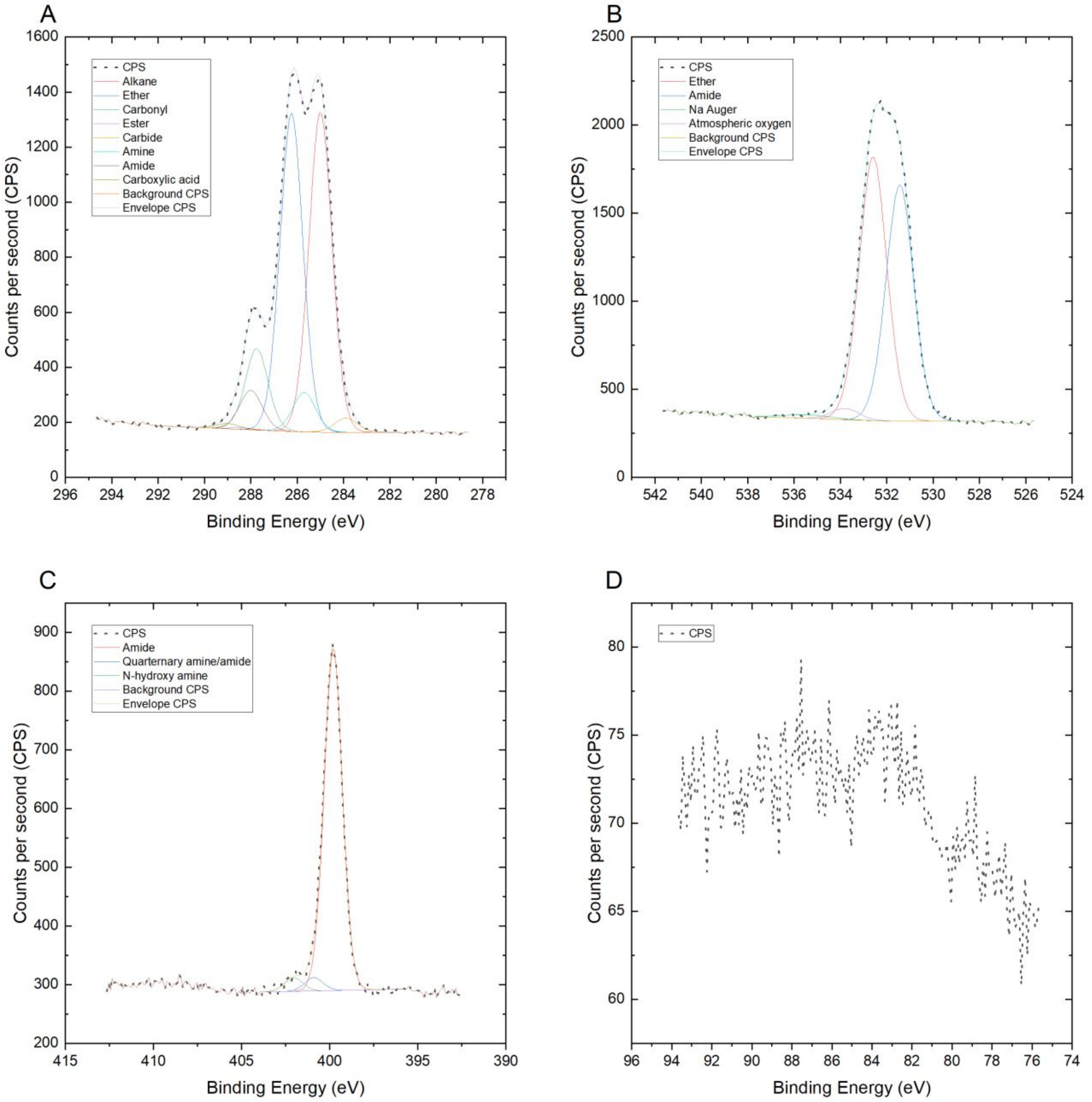

Figure S124 – XPS of Gal-2-pHEA42@AuNP40 A) C 1*s* B) O 1*s* C) N 1*s* and D) Au 4*f*

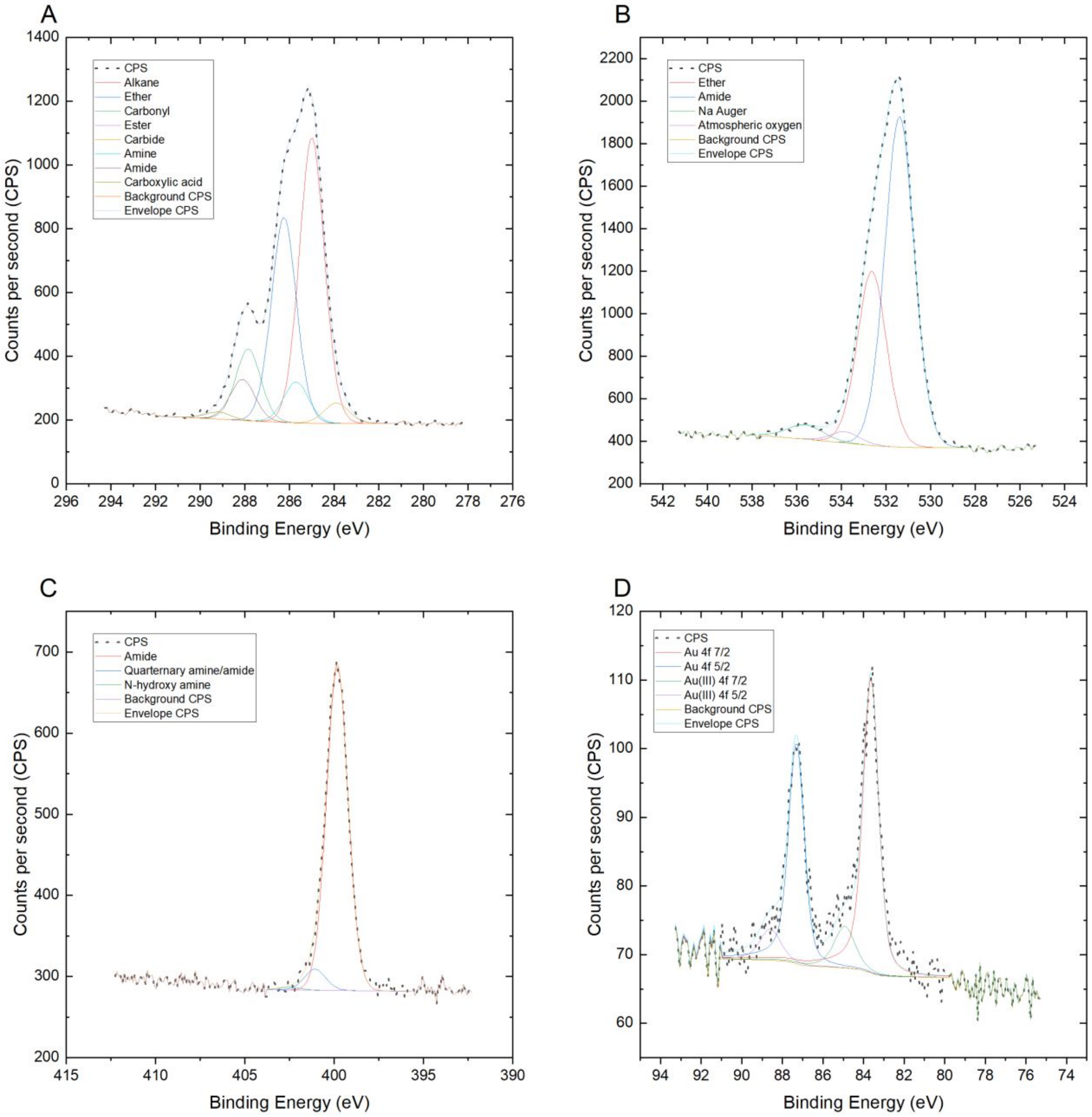

Figure S125 – XPS of Glc-2-pHEA42@AuNP16 A) C 1*s* B) O 1*s* C) N 1*s* and D) Au 4*f*

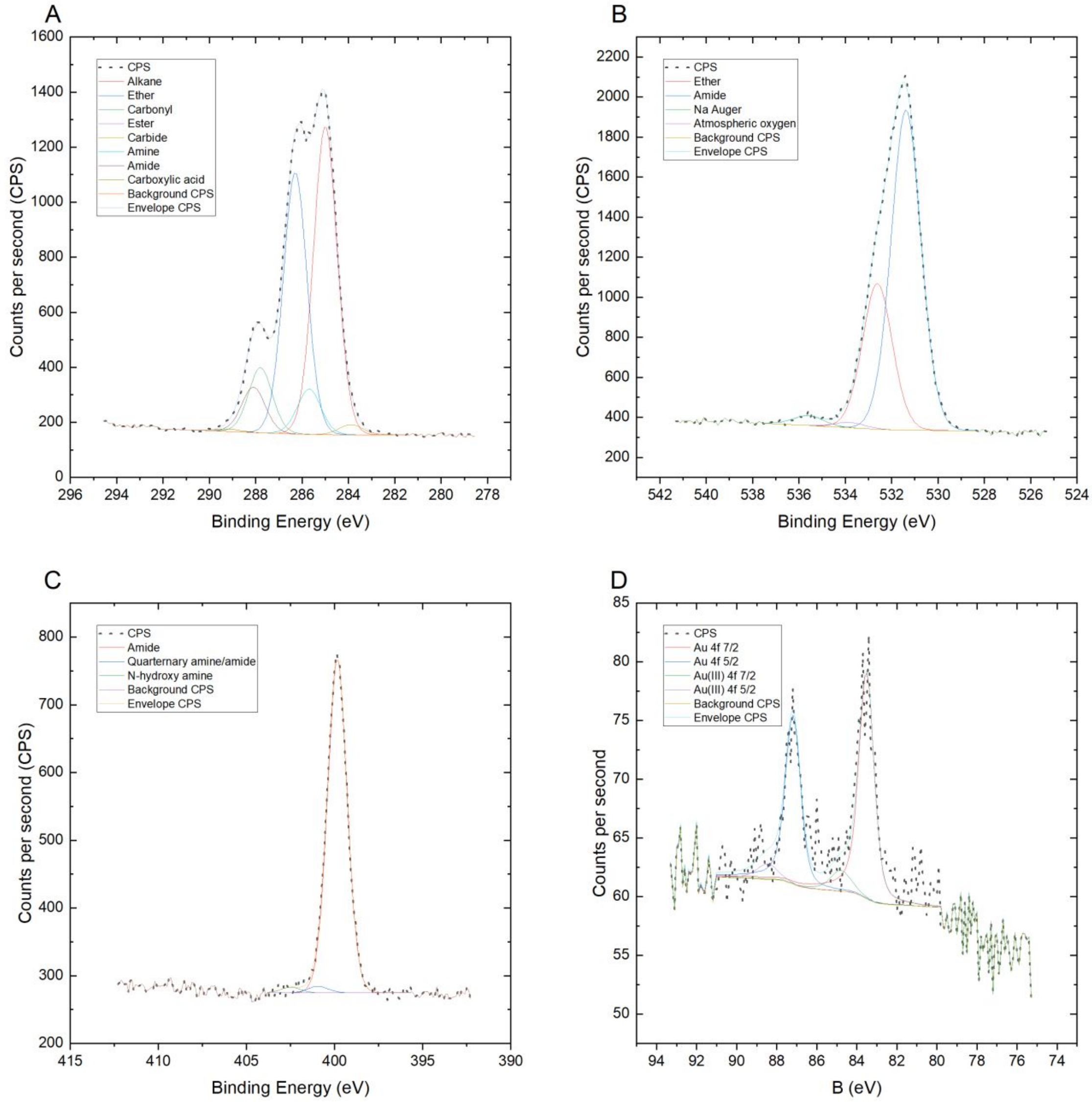

Figure S126 – XPS of Man-2-pHEA42@AuNP16 A) C 1*s* B) O 1*s* C) N 1*s* and D) Au 4*f*

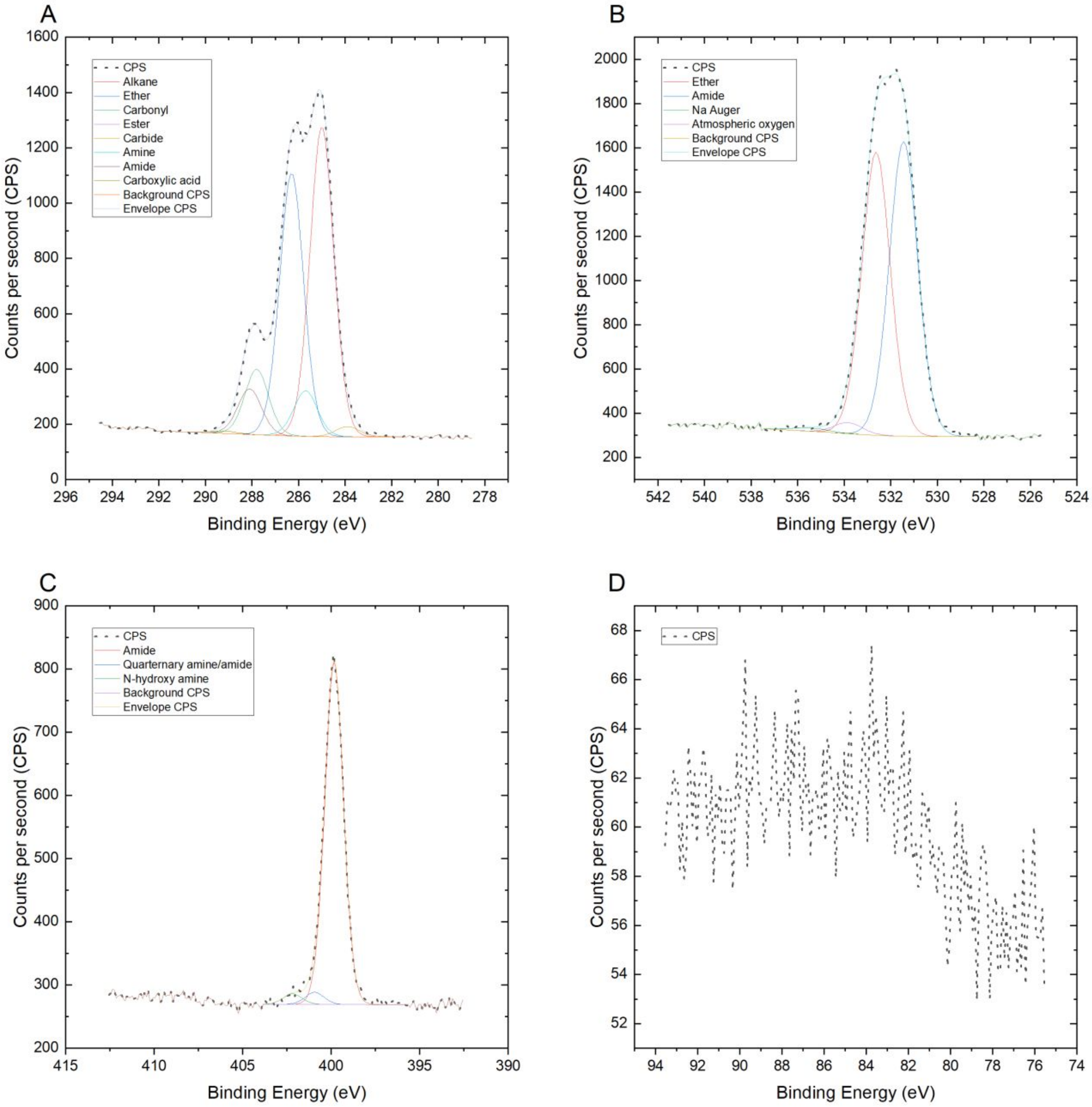

Figure S127 – XPS of Man-2-pHEA42@AuNP40 A) C 1*s* B) O 1*s* C) N 1*s* and D) Au 4*f*

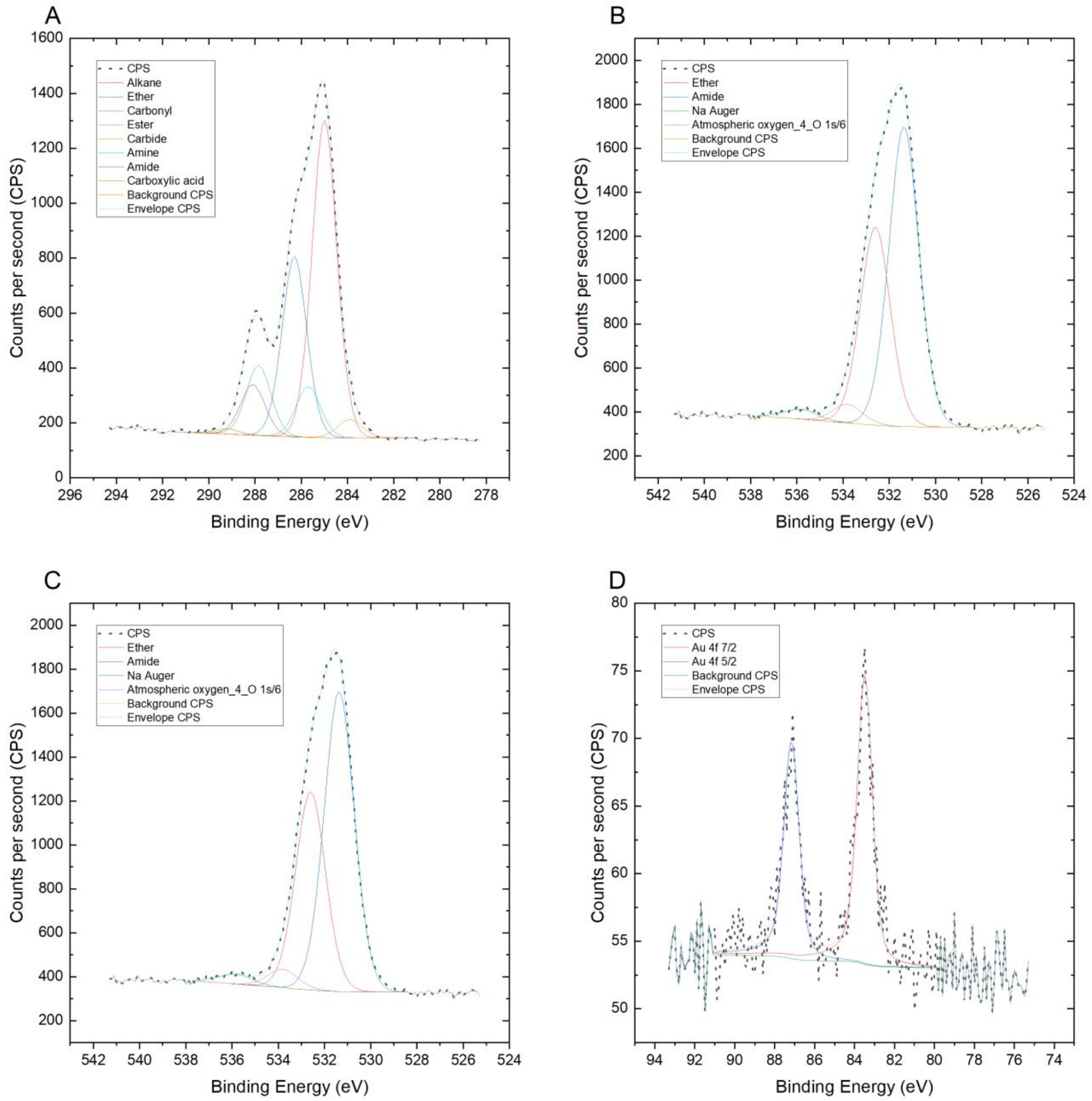

Figure S128 – XPS of Lac-1-pHEA25@AuNP16 A) C 1*s* B) O 1*s* C) N 1*s* and D) Au 4*f*

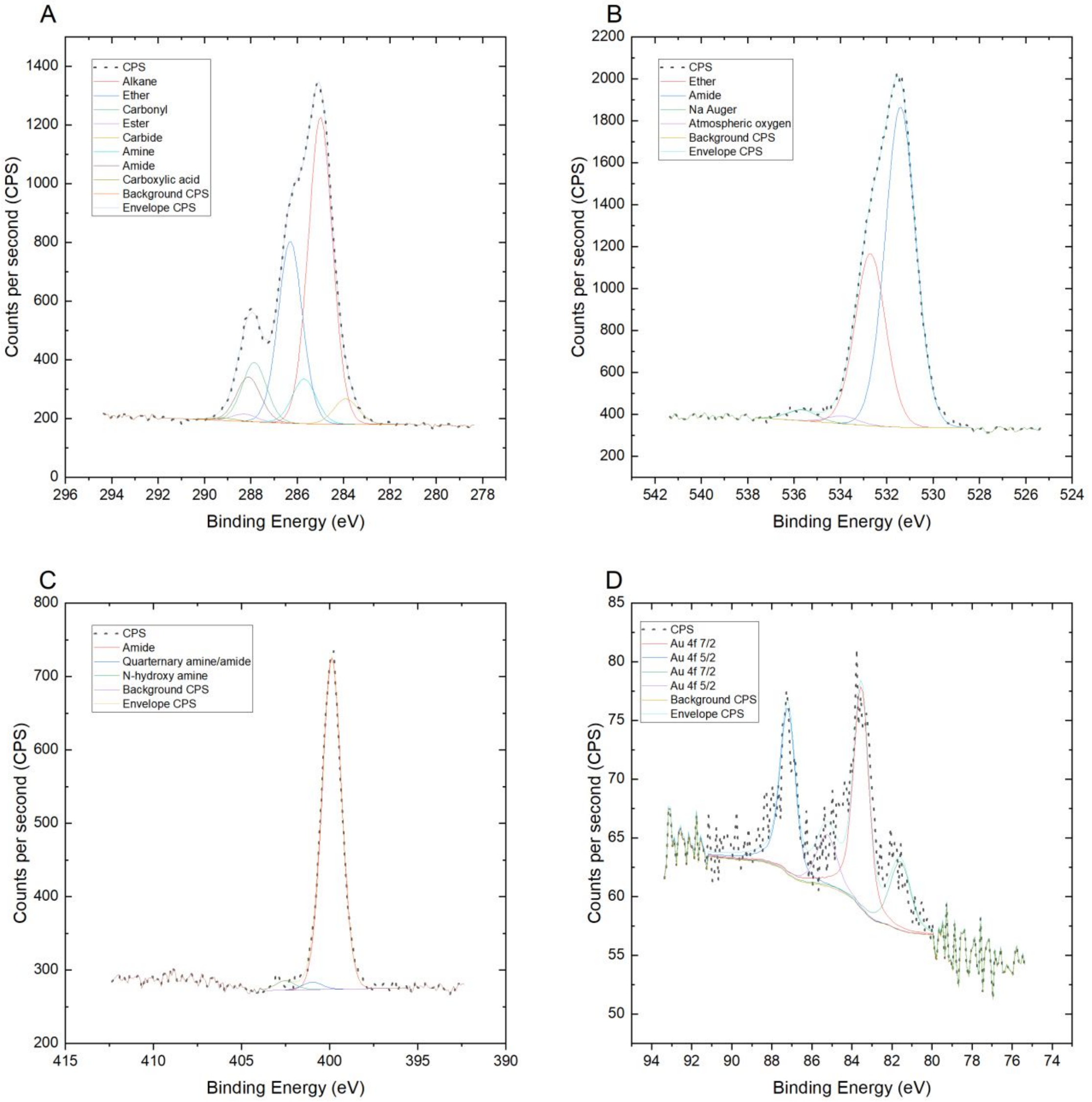

Figure S129 – XPS of Lac-1-pHEA42@AuNP16 A) C 1*s* B) O 1*s* C) N 1*s* and D) Au 4*f*

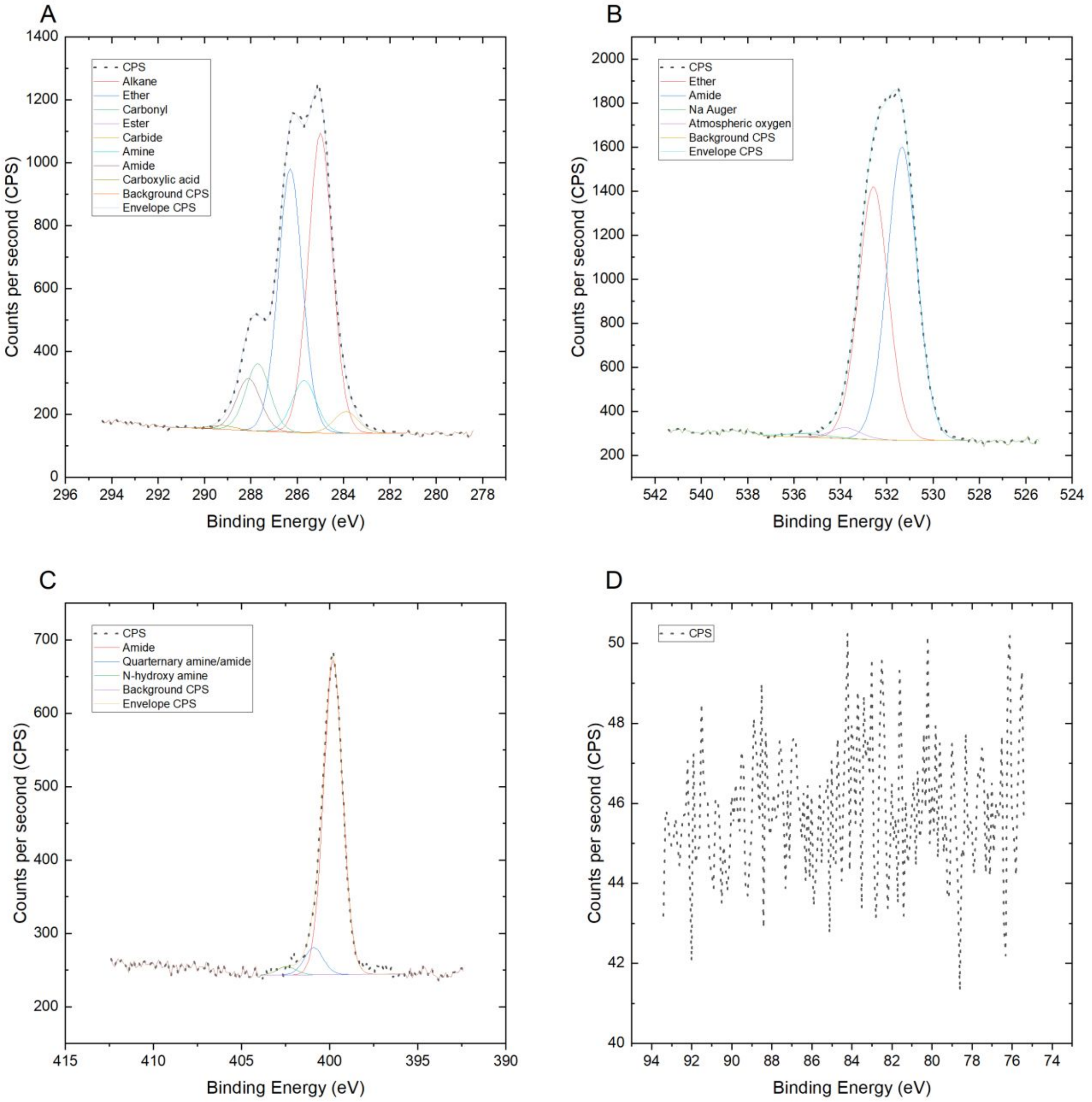

Figure S130 – XPS of Lac-1-pHEA25@AuNP30 A) C 1*s* B) O 1*s* C) N 1*s* and D) Au 4*f*

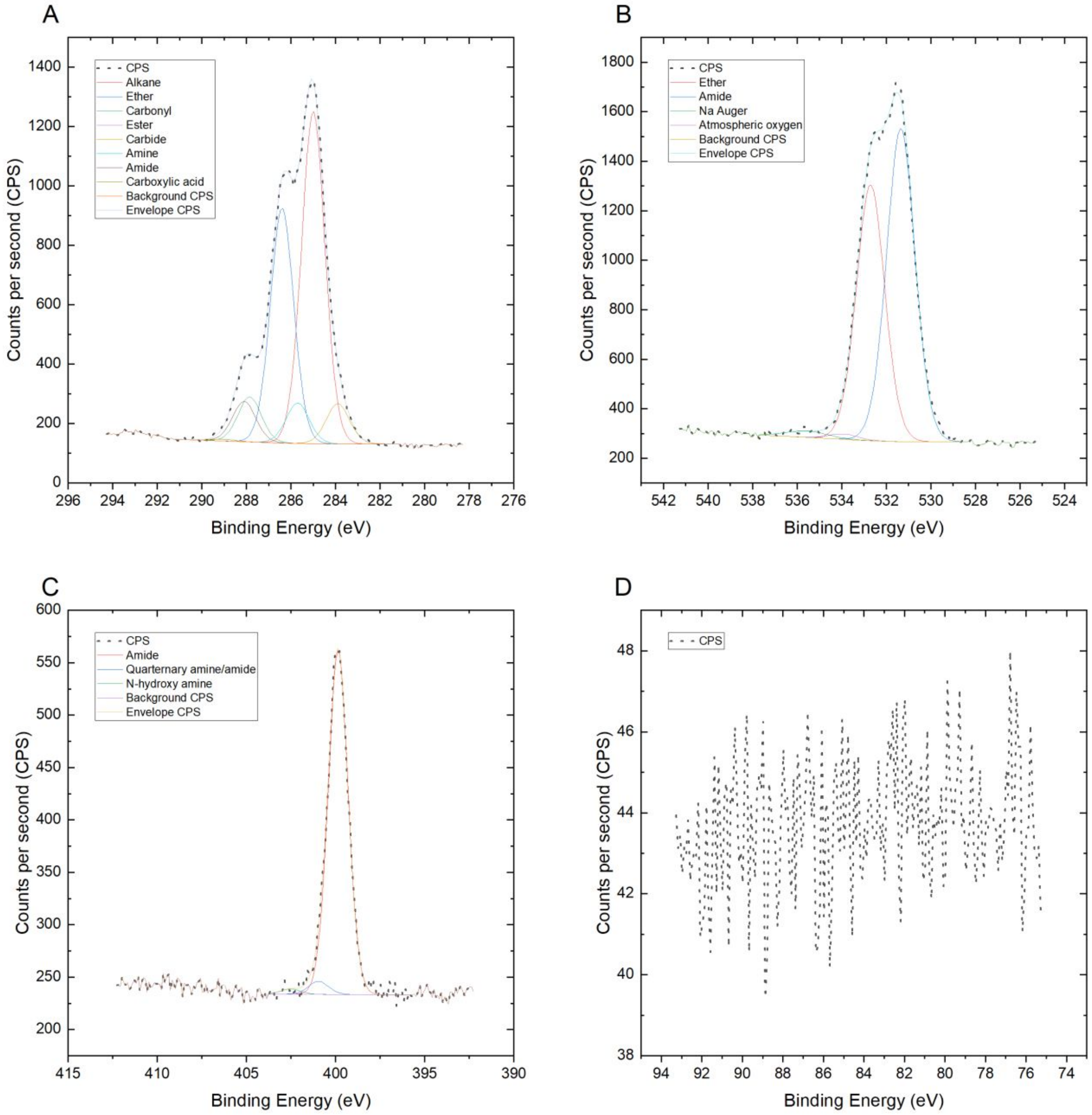

Figure S131 – XPS of Lac-1-pHEA52@AuNP40 A) C 1*s* B) O 1*s* C) N 1*s* and D) Au 4*f*

Particle	Elemental Percentage Composition (%)				Elemental Ratios	
	C 1*s*	O 1*s*	N 1*s*	Au 4*f*	N 1*s*/C 1*s*	N 1*s*/Au 4*f*
Unfunctionalized (citrate-stabilised) AuNP 16 nm	53.0	45.8	0.1	1.0	0.0	0.1
GalNAc-pHEA42@AuNP16	63.8	27.0	9.2	0.0	0.1	229.0
GalNAc-pHEA42@AuNP40	67.1	25.9	7.2	0.0	0.1	143.8
Gal-1-pHEA42@AuNP16	64.4	26.6	8.9	0.0	0.1	446.5
Gal-1-pHEA42@AuNP40	67.1	26.5	6.4	0.0	0.1	
Gal-2-pHEA25@AuNP16	67.7	23.1	9.2	0.1	0.1	130.9
Gal-2-pHEA42@AuNP16	65.8	25.2	8.9	0.0	0.1	178.8
Gal-2-pHEA25@AuNP30	67.9	25.4	6.7	0.0	0.1	
Gal-2-pHEA42@AuNP40	66.2	25.8	8.0	0.0	0.1	
Glc-2-pHEA42@AuNP16	63.1	29.0	7.8	0.1	0.1	59.6
Man-2-pHEA42@AuNP16	64.0	27.5	8.5	0.1	0.1	141.2
Man-2-pHEA42@AuNP40	65.9	25.9	8.2	0.0	0.1	
Lac-1-pHEA25@AuNP16	66.3	25.2	8.4	0.0	0.1	169.0
Lac-1-pHEA42@AuNP16	63.9	28.1	7.8	0.1	0.1	112.1
Lac-1-pHEA25@AuNP30	64.9	27.7	7.4	0.0	0.1	
Lac-1-pHEA52@AuNP40	68.3	26.0	5.6	0.0	0.1	

Table S2 – Elemental composition of nanoparticles determined by XPS

Particle	C 1*s* Bond Percentage Composition (%)								Bond Ratios	
	Alkane	Ether	Carbonyl	Ester	Carbide	Amine	Amide	Carboxylic Acid	Amide/Alkane	Amide/Ether
Unfunctionalized (citrate-stabilised) AuNP 16 nm	54.8	14.3	2.3	22.6	1.0	0.0	0.0	4.9	0.0	0.0
GalNAc-pHEA42@AuNP16	45.7	27.0	12.0	0.0	3.7	5.7	5.7	0.3	0.1	0.2
GalNAc-pHEA42@AuNP40	43.7	33.8	8.4	0.0	2.3	5.7	5.7	0.5	0.1	0.2
Gal-1-pHEA42@AuNP16	45.8	27.0	8.0	3.3	2.1	6.9	6.9	0.0	0.1	0.2
Gal-1-pHEA42@AuNP40	39.1	39.5	8.6	1.0	2.2	4.3	4.3	0.9	0.1	0.1
Gal-2-pHEA25@AuNP16	46.4	28.0	12.8	0.2	1.7	5.1	5.1	0.8	0.1	0.2
Gal-2-pHEA42@AuNP16	46.0	29.2	11.2	0.0	3.0	5.3	5.3	0.0	0.1	0.2
Gal-2-pHEA25@AuNP30	42.3	35.1	8.0	0.0	3.0	5.4	5.4	0.8	0.1	0.1
Gal-2-pHEA42@AuNP40	39.0	38.8	10.0	0.2	1.7	4.8	4.8	0.6	0.1	0.1
Glc-2-pHEA42@AuNP16	42.5	30.5	10.7	0.0	3.0	6.1	6.1	1.0	0.1	0.2
Man-2-pHEA42@AuNP16	45.4	28.8	10.8	0.0	3.0	6.0	6.0	0.0	0.1	0.2
Man-2-pHEA42@AuNP40	41.8	35.4	8.8	0.0	1.4	6.1	6.1	0.3	0.1	0.2
Lac-1-pHEA25@AuNP16	45.8	26.0	10.1	0.0	2.6	7.3	7.3	0.7	0.2	0.3
Lac-1-pHEA42@AuNP16	45.6	27.0	8.8	1.1	3.8	6.6	6.6	0.4	0.1	0.2
Lac-1-pHEA25@AuNP30	39.4	34.7	8.9	0.0	2.8	6.8	6.8	0.5	0.2	0.2
Lac-1-pHEA52@AuNP40	45.2	32.0	6.1	0.0	5.5	5.5	5.5	0.2	0.1	0.2

Table S3 – C 1*s* bonding composition nanoparticles determined by XPS

Biolayer Interferometry Data

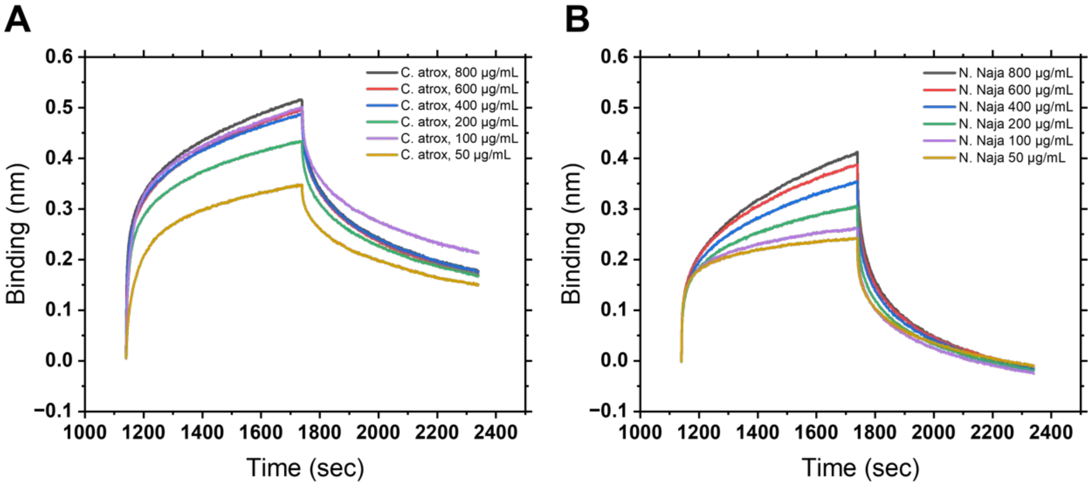

Figure S132 – BLI analysis of Gal-2-pHEA$_{42}$@AuNP$_{40}$ immobilised on aminopropylsilane sensors versus A) *C. atrox* venom, and B) *N. naja* venom in solution

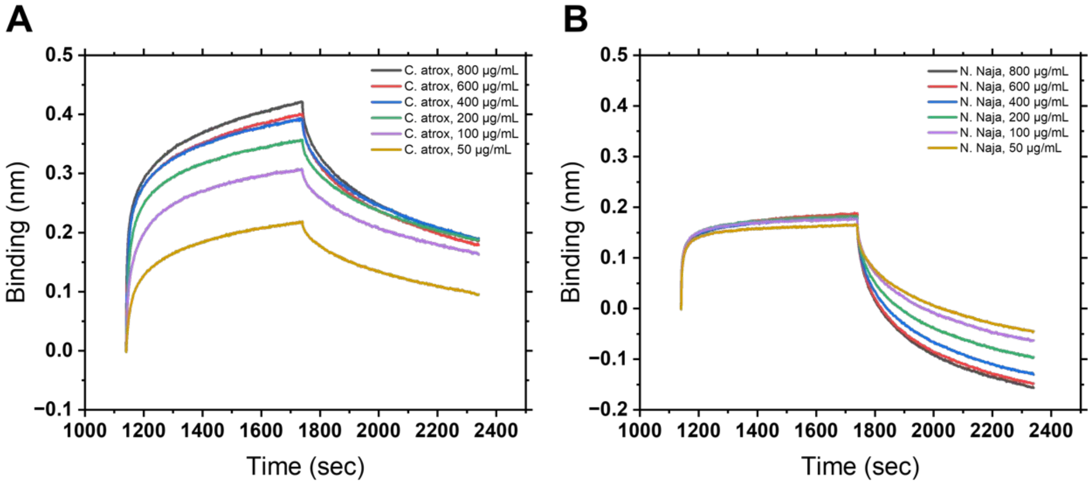

Figure S133 – BLI analysis of Glc-2-pHEA$_{42}$@AuNP$_{16}$ immobilised on aminopropylsilane sensors versus A) *C. atrox* venom, and B) *N. naja* venom in solution

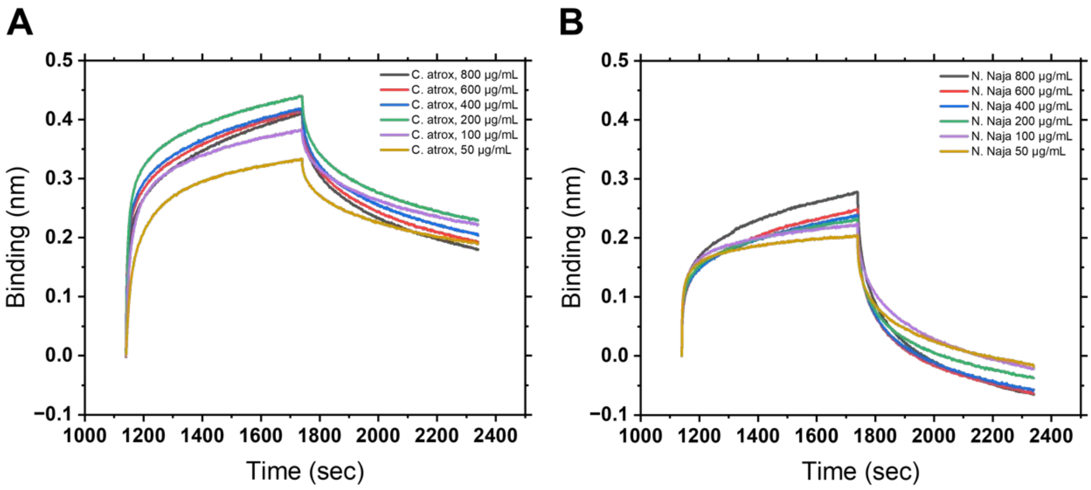

Figure S134 – BLI analysis of Man-2-pHEA$_{42}$@AuNP$_{16}$ immobilised on aminopropylsilane sensors versus A) *C. atrox* venom, and B) *N. naja* venom in solution

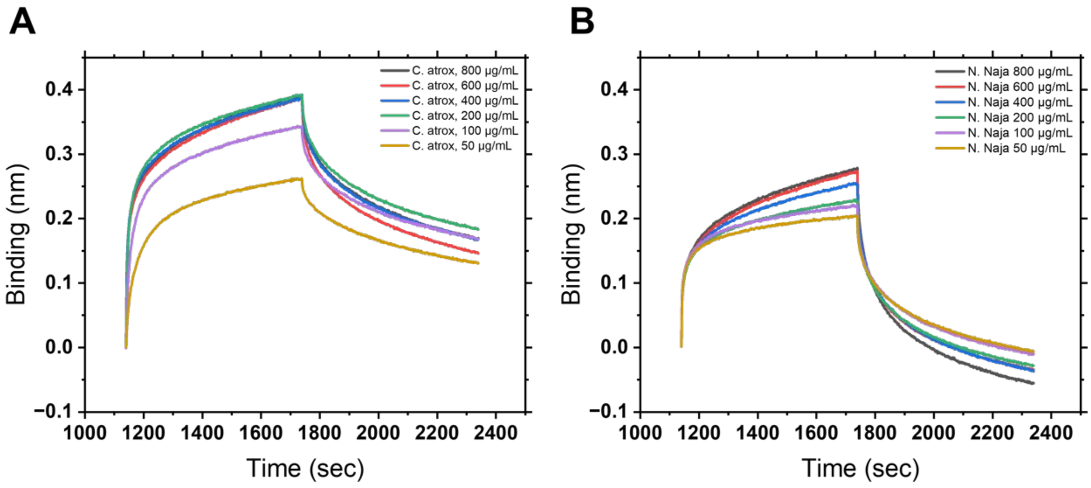

Figure S135 – BLI analysis of Lac-1-pHEA$_{42}$@AuNP$_{16}$ immobilised on aminopropylsilane sensors versus A) *C. atrox* venom, and B) *N. naja* venom in solution

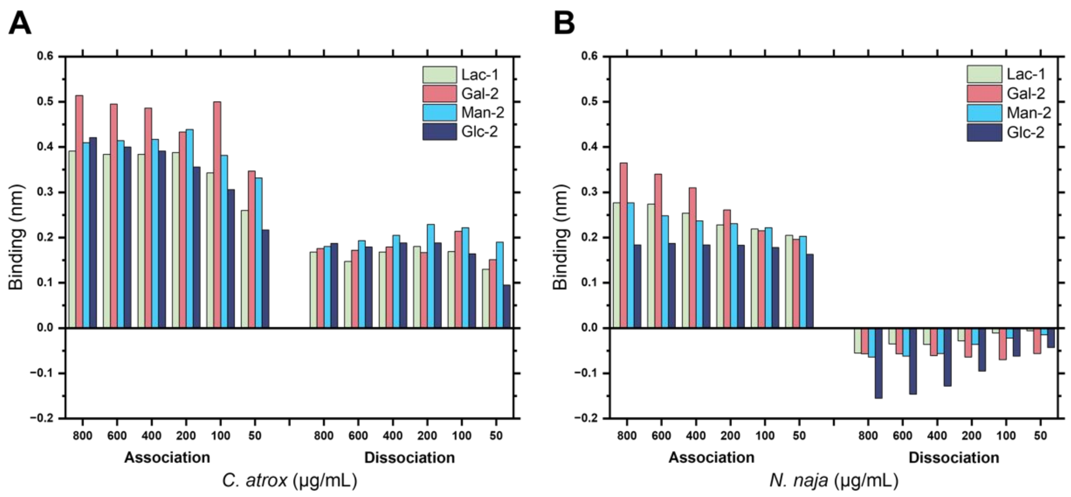

Figure S136 – Comparison of BLI analysis of nanoparticle systems immobilised on aminopropylsilane sensors versus A) *C. atrox* venom, and B) *N. naja* venom in solution. For the bar chart, the highest values from the association phase and the lowest values from the dissociation phase were used.

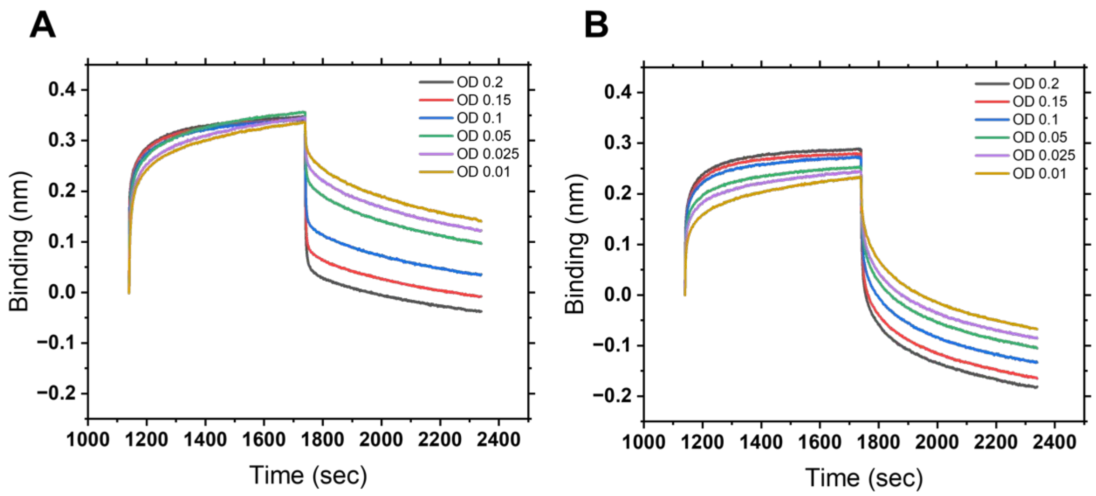

Figure S137 – BLI analysis of Gal-2-pHEA$_{42}$@AuNP$_{40}$ immobilised on aminopropylsilane sensors versus A) 400 µg/mL *C. atrox* venom, and B) 400 µg/mL *N. naja* venom in solution in competition with Gal-2-pHEA$_{42}$@AuNP$_{40}$ at varying ODs

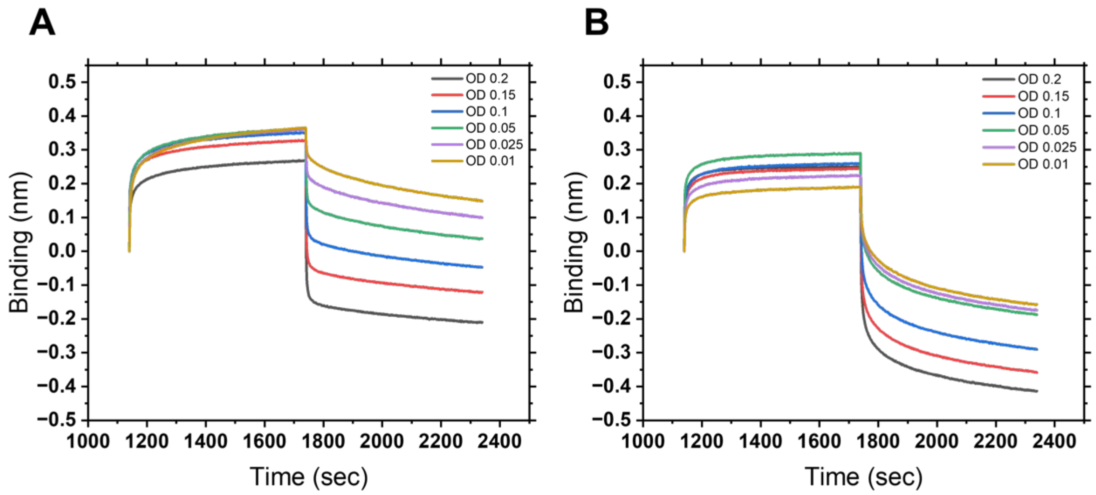

Figure S138 – BLI analysis of Glc-2-pHEA$_{42}$@AuNP$_{16}$ immobilised on aminopropylsilane sensors versus A) 400 µg/mL *C. atrox* venom, and B) 400 µg/mL *N. naja* venom in solution in competition with Glc-2-pHEA$_{42}$@AuNP$_{16}$ at varying ODs

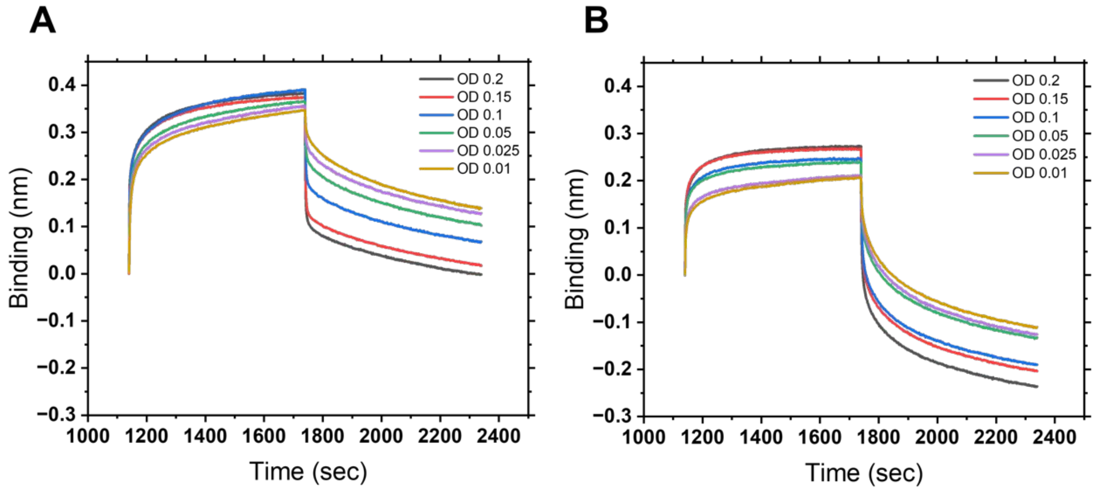

Figure S139 – BLI analysis of Man-2-pHEA$_{42}$@AuNP$_{16}$ immobilised on aminopropylsilane sensors versus A) 400 µg/mL *C. atrox* venom, and B) 400 µg/mL *N. naja* venom in solution in competition with Man-2-pHEA$_{42}$@AuNP$_{16}$ at varying ODs

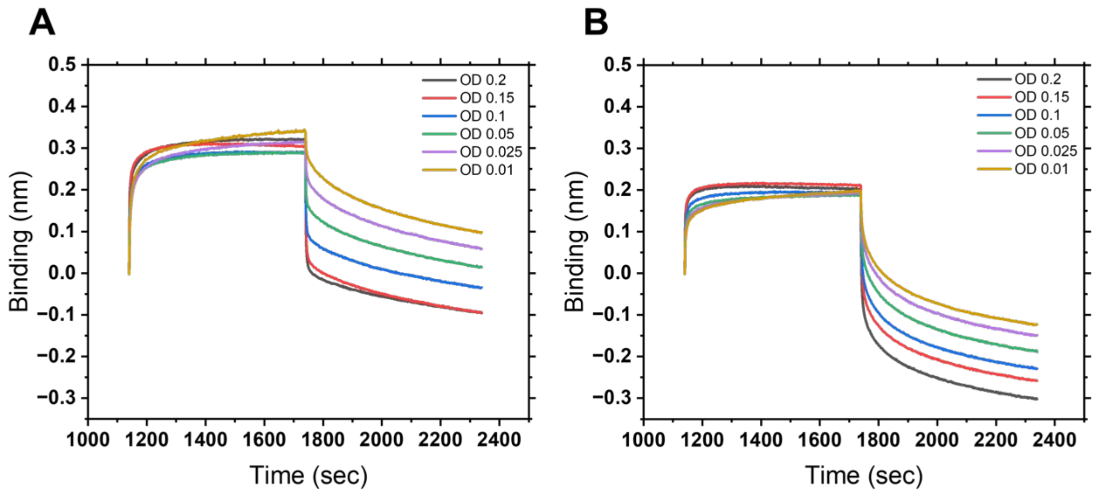

Figure S140 – BLI analysis of Lac-1-pHEA$_{42}$@AuNP$_{16}$ immobilised on aminopropylsilane sensors versus A) 400 µg/mL *C. atrox* venom, and B) 400 µg/mL *N. naja* venom in solution in competition with Lac-1-pHEA$_{42}$@AuNP$_{16}$ at varying ODs

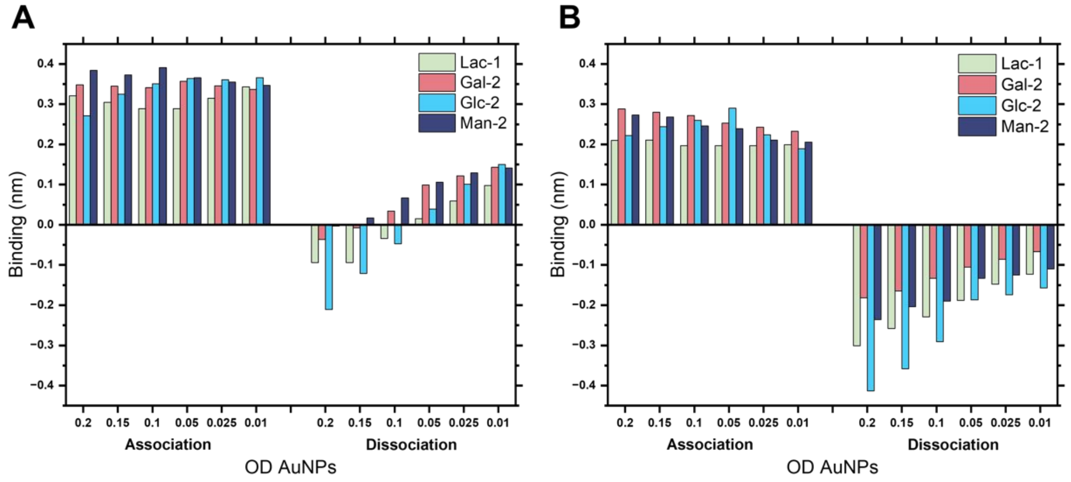

Figure S141 – Comparison of BLI analysis of nanoparticles immobilised on aminopropylsilane sensors versus A) 400 µg/mL *C. atrox* venom, and B) 400 µg/mL *N. naja* venom in solution in competition with nanoparticles at varying ODs. For the bar chart, the highest values from the association phase and the lowest values from the dissociation phase were used.

MALDI-MS

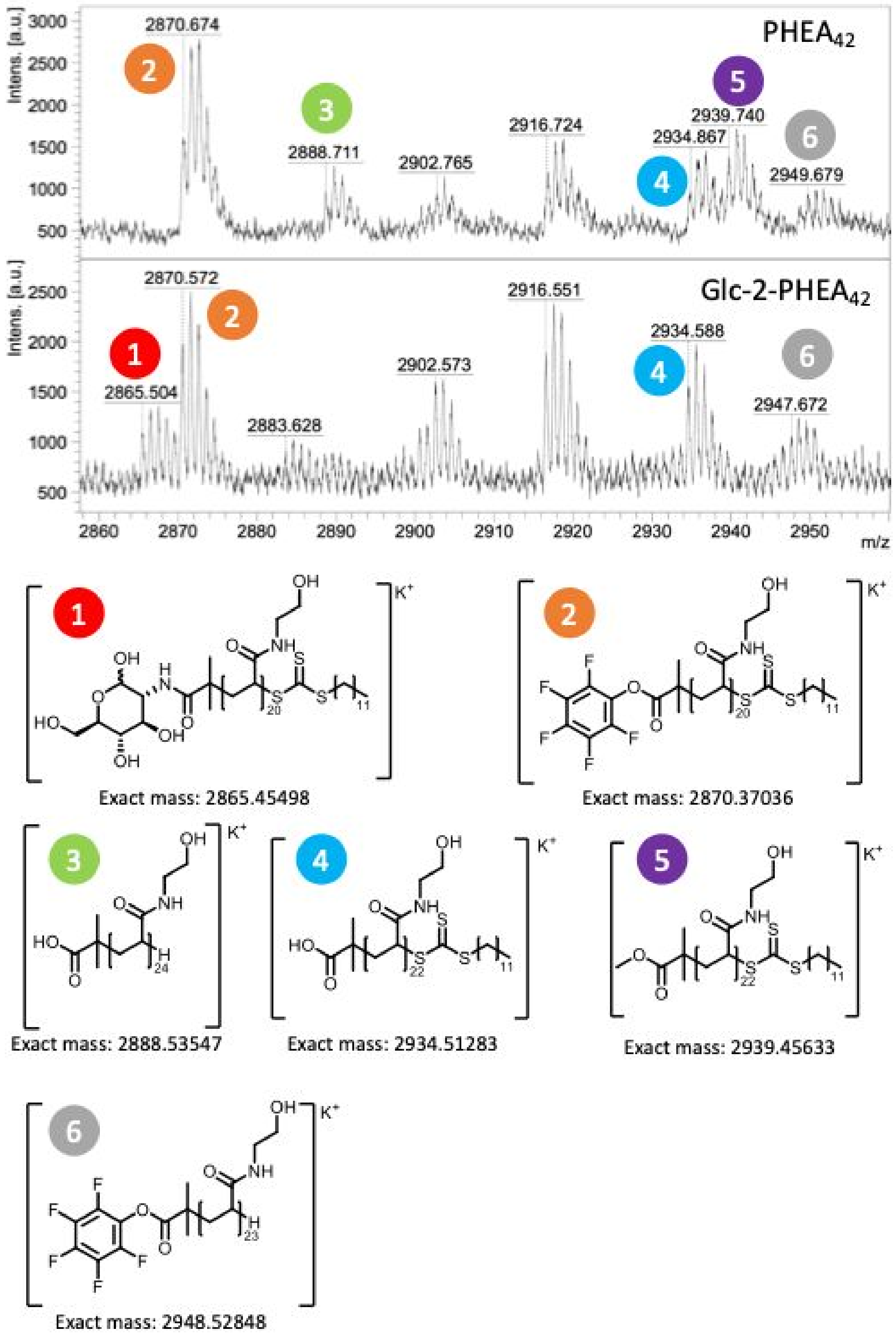

Figure S142 – MALDI-TOF mass spectra of $pHEA_{42}$ and Glc-2-$pHEA_{42}$ and predicted masses for possible polymer structures.,

References

1 S.-J. Richards and M. I. Gibson, Optimization of the polymer coating for glycosylated gold nanoparticle biosensors to ensure stability and rapid optical readouts, *ACS Macro Lett*, 2014, **3**, 1004–1008.

2 C. Campa, I. Donati, A. Vetere, A. Gamini and S. Paoletti, Synthesis of Glycosylamines: Identification and Quantification of Side products, *J Carbohydr Chem*, 2001, **20**, 263–273.

3 A. Lubineau, J. Augé and B. Drouillat, Improved synthesis of glycosylamines and a straightforward preparation of N-acylglycosylamines as carbohydrate-based detergents, *Carbohydr Res*, 1995, **266**, 211–219.

4 N. S. Ieong, K. Brebis, L. E. Daniel, R. K. O'Reilly and M. I. Gibson, The critical importance of size on thermoresponsive nanoparticle transition temperatures: Gold and micelle-based polymer nanoparticles, *Chemical Communications*, 2011, **47**, 11627–11629.
